# Vicinal difluorination as a C=C surrogate: an analog of piperine with enhanced solubility, photostability, and acetylcholinesterase inhibitory activity

**DOI:** 10.3762/bjoc.16.216

**Published:** 2020-10-28

**Authors:** Yuvixza Lizarme-Salas, Alexandra Daryl Ariawan, Ranjala Ratnayake, Hendrik Luesch, Angela Finch, Luke Hunter

**Affiliations:** 1 School of Chemistry, University of New South Wales (UNSW), Sydney NSW 2052, Australia; 2 Department of Medicinal Chemistry and Center for Natural Products, Drug Discovery and Development (CNPD3), University of Florida, Gainesville FL 32610, United States; 3 School of Medical Sciences, University of New South Wales (UNSW), Sydney NSW 2052, Australia

**Keywords:** Alzheimer's disease, bioisostere, conformational analysis, gauche effect, stereoselective synthesis

## Abstract

Piperine, a natural product derived from peppercorns, has a variety of biological activities that make it an attractive lead compound for medicinal chemistry. However, piperine has some problematic physicochemical properties including poor aqueous solubility and a susceptibility to UV-induced degradation. In this work, we designed an analog of piperine in which the central conjugated hydrocarbon chain is replaced with a vicinal difluoroalkane moiety. We show that this fluorinated analog of piperine has superior physicochemical properties, and it also has higher potency and selectivity towards one particular drug target, acetylcholinesterase. This work highlights the potential usefulness of the *threo*-difluoroalkane motif as a surrogate for *E*-alkenes in medicinal chemistry.

## Introduction

**Figure 1:**
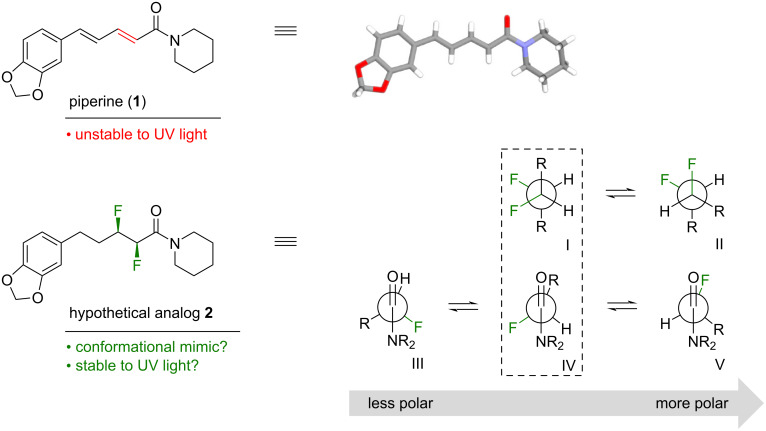
The natural product piperine (**1**) is the inspiration for this work; the crystal structure is shown [14]. In this work, a hypothetical analog **2** was designed to mimic parent compound **1**. The predicted low-energy rotamers of **2** about the F–C–C–F and F–C–C=O bonds are shown; rotamers I and IV give the best mimicry of **1**.

Piperine (**1**, Figure 1) is a well-known natural product that is derived from peppercorns [1–3]. Many biological studies of **1** have been carried out, and these studies have led to a diverse array of biological activities being claimed for this compound [4–9]. For example, **1** is reported to exhibit inhibitory activity towards both acetylcholinesterase (AChE) and β-secretase (BACE-1), which suggests that **1** could hold promise as a dual mechanism-of-action treatment for Alzheimer’s disease [4,10–13].

However, piperine (**1**) has some limitations as a drug lead. For example, it has poor solubility, and it is susceptible to photoisomerization of the conjugated system [15–17]. This prompted us to consider whether there was another structural motif that could (i) mimic the extended geometry of an *E-*alkene, (ii) impart better solubility, and (iii) be more stable in the presence of UV light. Such a C=C surrogate might offer an improved lead compound for the development of drugs to treat Alzheimer’s disease.

Stereoselective fluorination is an emerging strategy for controlling the conformations of organic molecules. The highly polarized C–F bond tends to align in predictable ways with adjacent functional groups, due to a combination of hyperconjugative and/or dipolar interactions [18–21]. This knowledge led us to propose the hypothetical analog **2** (Figure 1) as a potential mimic of **1**. The analog **2** contains a saturated alkyl chain with two vicinal C–F bonds in the α/β positions relative to the amide moiety. The presence of a vicinal difluoride moiety within an alkyl chain is known to favour rotamers in which the C–F bonds align *gauche* (I and II, Figure 1) [22]. Separately, the presence of fluorine on the α-carbon of a tertiary amide is known to restrict the C_α_–C(O) bond to a small set of low-energy rotamers (III–V, Figure 1) [23]. Intriguingly, studies of simple molecules containing either F–C–C–F or F–C–C=O motifs have shown that the rotamer populations can change, depending on the polarity of the solvent [22–23]. The rotamers I and IV (Figure 1, boxed) would appear to provide the closest structural match with compound **1**, but in a highly polar medium the more polar rotamers II and V might predominate. Of course, the contiguous positioning of the F–C–C–F and F–C–C=O moieties within the same molecule (**2**) means that these moieties would not function independently, but rather would likely influence one another, adding another layer of complexity. Finally, it is interesting to consider whether the microenvironment of a protein binding site could also change the relative energies of the various F–C–C–F and F–C–C=O rotamers, offering the possibility that analog **2** might be an effective conformational mimic of **1** in some environments but not in others.

Herein, we describe the optimisation of a synthetic route to compound **2**, the conformational analysis of this molecule by NMR and molecular modelling studies, a comparison of the photostabilities of parent compound **1** vs derivative **2**, and a comparison of the inhibitory activities of **1** vs **2** towards both AChE and BACE-1.

## Results and Discussion

### Synthesis

**Scheme 1:**
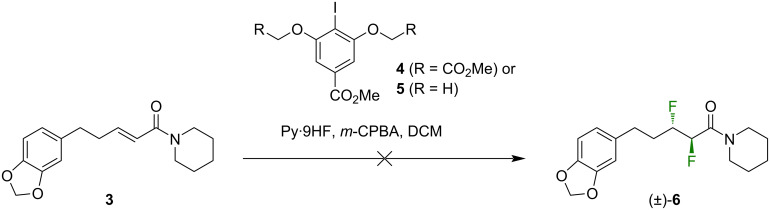
The attempted synthesis of **6** (a diastereoisomer of **2**) via a one-step 1,2-difluorination reaction [24]. Py = pyridine; *m*-CPBA = *m*-chloroperbenzoic acid, DCM = dichloromethane.

Jacobsen and co-workers have recently described a method for the one-step, diastereoselective 1,2-difluorination of alkenes, mediated by a hypervalent iodine catalyst [24]. The substrate scope of the Jacobsen method has certain constraints but their original report did include some examples of α,β-unsaturated amides, and so we were motivated to investigate the method in this work, using compound **3** [25] as the substrate (Scheme 1). We recognized that the product of this reaction would most likely be the unwanted *erythro*-isomer **6** but we nevertheless deemed it a worthwhile preliminary investigation. Disappointingly, the treatment of the precursor **3** with pyridine·HF and either catalyst **4** or **5** at room temperature for two days gave no discernible reaction, and the starting material **3** was recovered intact. Warming the reaction mixture to 50 °C caused the disappearance of **3** but the formation of an intractable mixture of products. We were forced to conclude that the rather complex structure of **3** was incompatible with the Jacobsen catalytic system.

**Scheme 2:**
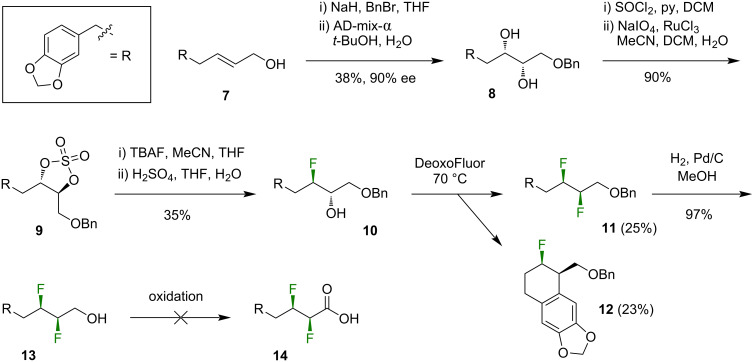
The attempted synthesis of **2** via a stepwise fluorination approach (ether series). THF = tetrahydrofuran, AD-mix-α = commercially available asymmetric dihydroxylation reagent, TBAF = tetrabutylammonium fluoride, DeoxoFluor = bis(2-methoxyethyl)aminosulfur trifluoride.

Since the one-step difluorination method (Scheme 1) was unsuccessful, we decided to pursue a stepwise fluorination approach [26–27] (Scheme 2). Thus, the allylic alcohol **7** [28] was protected as the benzyl ether then subjected to a Sharpless asymmetric dihydroxylation reaction to furnish the diol **8** in modest yield. The diol **8** was then converted into the cyclic sulfate **9**, which was ring-opened using TBAF to furnish the fluorohydrin **10**. A Mosher ester analysis of the fluorohydrin **10** suggested that the earlier dihydroxylation reaction had proceeded with 90% ee. The deoxyfluorination of **10** was then attempted using several reagents including DeoxoFluor, DeoxoFluor in combination with TMS-morpholine [29], and PyFluor [30]. The optimal yield of the *threo*-difluoroalkane **11** was obtained with DeoxoFluor at elevated temperature (Scheme 2). A side-product in this fluorination reaction was the tricyclic compound **12**, which presumably formed through an electrophilic aromatic substitution reaction of the activated alcohol intermediate. The inclusion of TMS-morpholine [29] reduced the formation of this side-product but did not lead to an overall increase in the yield of **11**. Despite the modest optimised yield of the difluoroalkane **11**, a sufficient quantity of this material was secured to continue with the synthesis. The hydrogenolysis of the benzyl ether of **11** provided the primary alcohol **13**, which only needed to be oxidised to the carboxylic acid **14** then coupled to piperidine in order to deliver the target compound, **2**. However, the oxidation of **13** proved to be unexpectedly troublesome. Mild oxidising agents caused incomplete consumption of **13**, while vigorous conditions led to the oxidation not only of the primary alcohol of **13** but also of the electron-rich aryl moiety (see Supporting Information File 1).

**Scheme 3:**
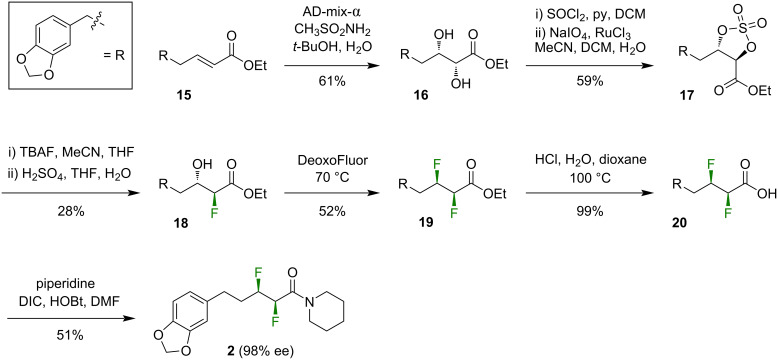
Synthesis of compound **2** via a stepwise fluorination approach (ester series). DIC = diisopropylcarbodiimide, HOBt = hydroxybenzotriazole, DMF = *N*,*N*-dimethylformamide.

In order to circumvent the troublesome oxidation reaction (i.e., **13→14**, Scheme 2), and to seek higher enantiopurity of the target, we modified the synthetic plan to include an ester moiety throughout (Scheme 3). Thus, the α,β-unsaturated ester **15** [25] was carried through a similar sequence to that previously described, i.e., dihydroxylation, cyclic sulfate formation, ring-opening with TBAF (although note the regioselectivity [31]), deoxyfluorination, and deprotection to deliver the difluorinated acid **20**. Finally, coupling of the acid **20** with piperidine afforded the target compound **2** in moderate yield (Scheme 3). The enantiopurity of **2** was investigated using chiral HPLC. From a spectroscopically pure sample of **2**, two HPLC peaks were observed, with an integral ratio of 99:1. It is assumed that the two peaks correspond to the two enantiomers of **2** and, if that assumption is true, then the optical purity of this sample is 98% ee.

### Conformational analysis

Having completed the synthesis of the target molecule **2**, the next task was to investigate the conformational behavior of this molecule. This was achieved by performing a DFT study in the Gaussian software, using the M06-2X level of theory with the 6-311+G(d,p) basis set, parameters similar to those employed by Linclau and co-workers for their studies of vicinal difluoro systems [22]. A set of starting structures of **2** was generated by systematically rotating three bonds (i.e., F–C–C–F, F–C–C=O, and O=C–N–C) in 120° increments and the starting structures were then geometry optimised and their energies calculated. To enable benchmarking against experiment, NMR spin–spin coupling constants were calculated for the lowest-energy final structures of **2** using the GIAO method with the B3LYP/6-311+G(d,p) level of theory. Chloroform was used as the solvent for both the NMR experiments and the SMD calculations.

**Figure 2:**
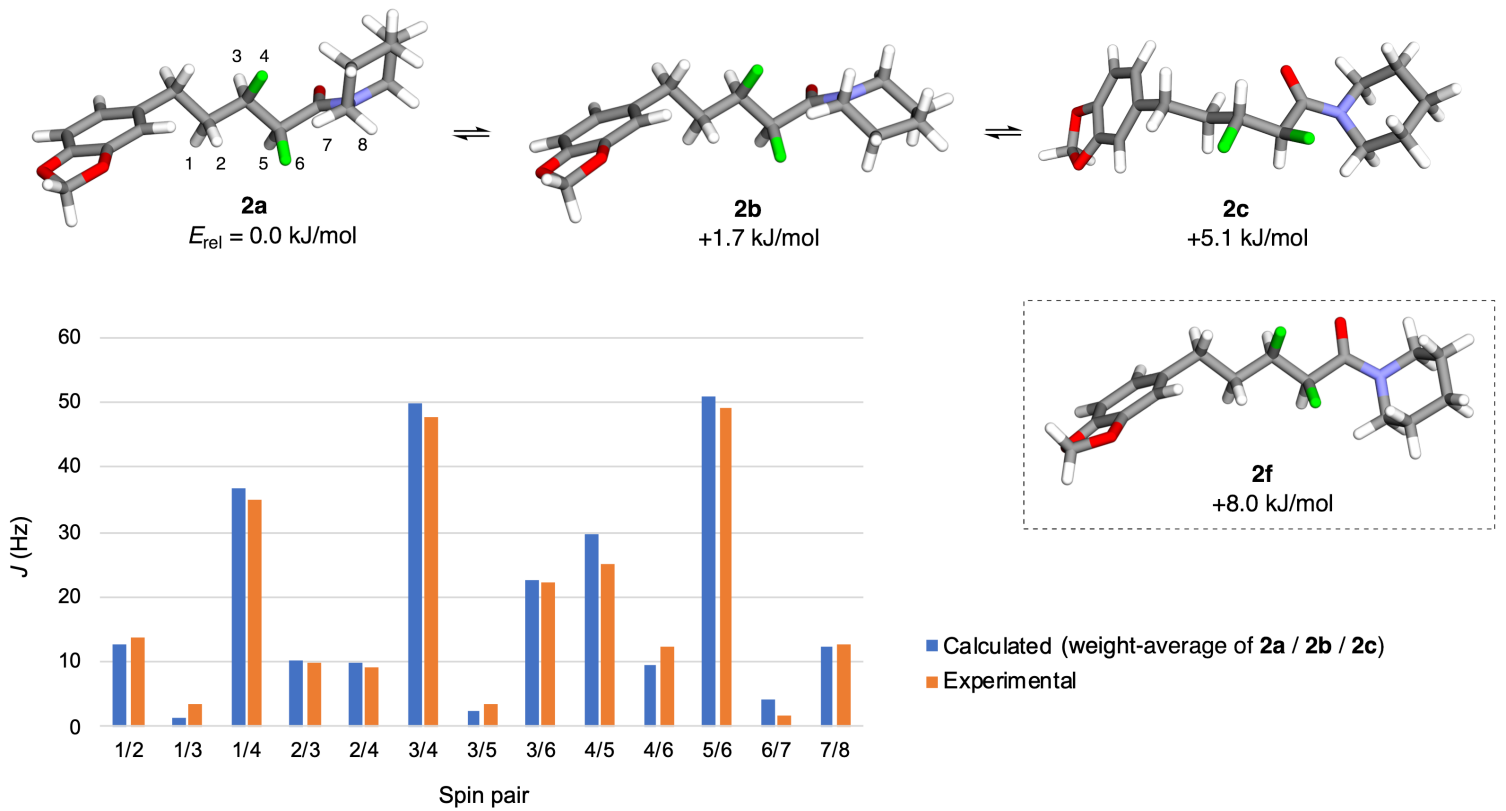
Conformational analysis of **2** by DFT and NMR. The numbering scheme for NMR spins is given on structure **2a**.

The three lowest-energy structures to emerge from the computational analysis (i.e., **2a**–**c**) are shown in Figure 2. The global minimum structure **2a** has a F–C–C–F dihedral angle of 73°, giving an extended zigzag carbon chain. This approximates rotamer I (Figure 1) and in this regard **2a** is a good conformational mimic of **1**. However, **2a** has a F–C–C=O dihedral angle of 152°, approximating rotamer III (Figure 1) and in this regard **2a** is a poorer conformational mimic of **1**. The next-higher energy structure (**2b**, Figure 2) is related to **2a** through a ring-flip of the piperidine moiety (equivalent to a 180° rotation of the amide bond). The structures **2a**,**b** are close in energy, suggesting that both puckers of the piperidine moiety would be substantially populated in solution. A similar situation occurs for piperine itself; an indirect evidence for this comes from the crystal structure of **1** (Figure 1), where there is a second molecule in the unit cell (not shown) that has the alternative ring pucker [14]. The next-higher energy calculated structure of **2** (i.e., **2c**, Figure 2) has a F–C–C–F dihedral angle of −52°. This approximates rotamer II (Figure 1), giving a bent carbon chain that contrasts with the extended chains of **2a**,**b**. The structure **2c** has a F–C–C=O dihedral angle of −118°; this approximates rotamer IV (Figure 1). Together, the structures **2a**–**c** seem to dominate the conformer population distribution of **2** in chloroform solution, because the weight-averaged calculated *J*-values of **2a**–**c** match the experimentally-measured *J*-values quite well (Figure 2). But none of the structures **2a**–**c** are a close conformational match with piperine (**1**).

A noteworthy feature of structures **2a**,**b** (Figure 2) is that both, the α-fluorine and the β-fluorine atoms of each structure make close contacts with the hydrogen atoms on the piperidine ring (2.08–2.34 Å). This manifests in the observation of a through-space coupling (*J* = 1.9 Hz) between the α-fluorine and a piperidine hydrogen in the experimental NMR spectrum of **2** (i.e., spins 6/7, Figure 2). The attractive F···H interactions might explain why the amide bond is twisted by 11–20° from planarity in **2a**,**b**. In structure **2c**, only the α-fluorine makes close contacts with hydrogens on the piperidine ring, and the amide is twisted to a lesser degree in that case (8° away from planarity). It is interesting that in the crystal structure of piperine itself (**1**, Figure 1) [14], the amide bond is also twisted by 15° away from planarity. In this case the distortion might be attributable to a H···H clash (1.99 Å).

Structure **2f** (Figure 2), which was among the higher-energy calculated conformers of **2** (see Supporting Information File 1), bears a close resemblance to the solid-state conformation of piperine (**1**, Figure 1). The structure **2f** has a F–C–C–F dihedral angle of 57° and a F–C–C=O dihedral angle of –133°. These angles approximate rotamers I and IV, respectively (Figure 1). The unexpectedly high relative energy that was calculated for compound **2f** might be partially attributable to the fact that it features only a single F···H contact within 2.50 Å. The calculations suggest that the conformer **2f** is not significantly populated in chloroform solution but it was not possible to verify this by NMR measurements, because **2f** is essentially superimposable with **2a**,**b** within the fluoroalkyl segment. In more polar solvents such as water, the intramolecular F···H interactions would be expected to decrease in significance [32] and this might increase the accessibility of conformer **2f**. We reasoned that comparing the biological activities of **1** vs **2** might shed light on this possibility (vide infra).

### Photostability

**Figure 3:**
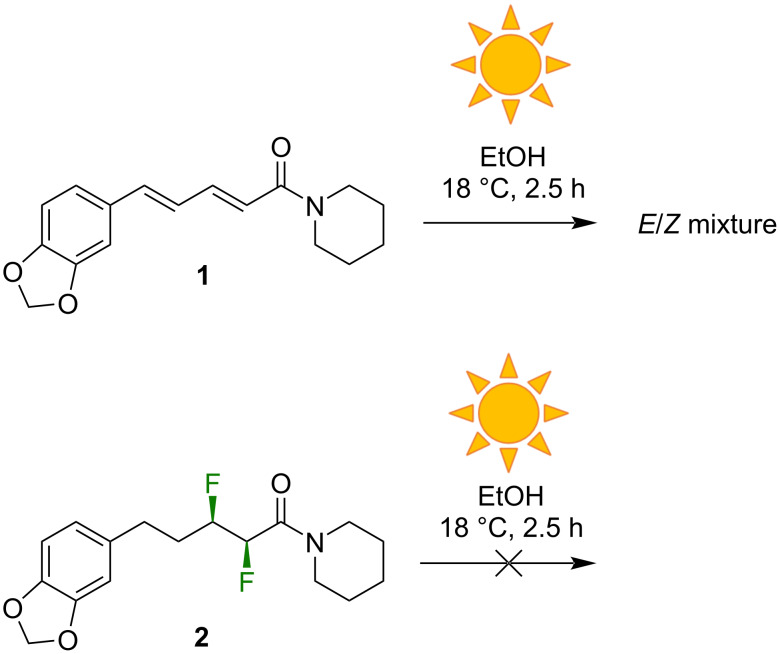
Analog **2** has greater stability to UV light than does piperine (**1**).

A limitation of piperine (**1**) as a drug lead is its instability under UV light. The conjugated system of **1** is well-known to undergo facile *E*/*Z* isomerization upon the absorption of UV photons, leading rapidly to a mixture of all four possible geometric isomers of **1** [15–17]. In the present work, this phenomenon was confirmed by exposing an ethanolic solution of **1** to sunlight for 2.5 h (Figure 3). The analysis of the product by ^1^H NMR spectroscopy revealed a multitude of new signals in the alkenyl region. In contrast, the analog **2** lacks conjugation in the central portion of the molecule and was therefore expected to be more stable to UV light. Indeed, the exposure of **2** to sunlight in an identical manner to that described for **1** led to no detectable decomposition (Figure 3, Supporting Information File 1).

### Biological activity and solubility

The biological activities of piperine (**1**) and the analog **2** were compared using two different assays, namely the inhibition of either acetylcholinesterase (AChE) or β-secretase (BACE-1).

**Figure 4:**
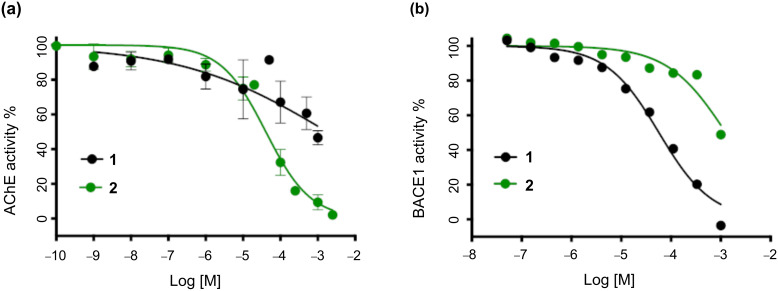
Biological activity of piperine (**1**) and derivative **2**. (a) Inihbition of AChE by **1** (IC_50_ >1000 μM) and **2** (IC_50_ = 51.7 μM) and (b) inhibition of BACE-1 by **1** (IC_50_ = 59.2 μM) and **2** (IC_50_ > 1000 μM).

The inhibition of AChE was measured using a modification of a previously described colorimetric assay (Figure 4a) [33]. It quickly became apparent that the limited solubility of piperine (**1**) in 50 mM Tris-HCl buffer, even in the presence of methanol as a co-solvent, was a major problem in this assay. We observed cloudiness or the appearance of a precipitate at higher concentrations of **1**, which affected the reproducibility of the assay, and prevented complete inhibition from being achieved (Figure 4a). The estimated IC_50_ of **1** was >1,000 μM. In contrast, analog **2** posed no solubility problems in this assay, generating a reproducible curve all the way to complete inhibition and returning an IC_50_ value of 51.7 μM. Thus, the replacement of the C=C fragment in **1** with a *threo*-difluoroalkane motif in **2** appears to preserve or enhance the AChE-binding ability, while simultaneously offering the advantage of improved aqueous solubility.

The inhibition of BACE-1 was measured using a fluorogenic peptide substrate according to an established method (Figure 4b) [34]. Intriguingly, the relative activities of **1** vs **2** were reversed in comparison with the AChE assay. The lead compound **1** achieved the complete inhibition of BACE-1 under the conditions of the assay, giving an IC_50_ value of 59.2 μM. In contrast, analog **2** was a much weaker inhibitor of BACE-1, failing to achieve complete inhibition, and giving an estimated IC_50_ value of >1,000 μM.

There are several conceivable explanations for the reversed relative activities of **1** vs **2** in the AChE vs BACE-1 assays. One possibility is that analog **2** is induced to adopt the “correct” conformation when binding to both targets (i.e., **2f**, Figure 2), and this structure fits well within the AChE active site but poorly within the BACE-1 active site, perhaps due to unfavourable interactions of the fluorine substituents of **2** with BACE-1 active site residues. A second possibility is that **2** adopts an “incorrect” conformation upon binding to both targets (e.g., **2a**, Figure 2), and this novel molecular shape is readily accommodated by AChE [35] but not by BACE-1, perhaps due to different levels of flexibility within the enzyme active sites. A third possibility is that the analog **2** adopts different conformations upon binding to AChE vs BACE-1, since the microenvironments within the enzymes’ active sites could be different (e.g., more/less polar), the “correct” binding geometry of **2** might be favoured in AChE but not in BACE-1. These possibilities all remain speculative in the absence of high-resolution structural data of the enzyme–ligand complexes.

## Conclusion

A *threo*-difluorinated piperine analog (**2**) was successfully synthesised through a stepwise route. The physicochemical properties of compound **2** were found to be superior to piperine (**1**) itself in two key respects, namely photostability and aqueous solubility. The conformational analysis of **2** by DFT and NMR spectroscopy revealed that the lowest-energy conformations **2a**–**c** are imperfect mimics of **1** but that a somewhat higher-energy conformation (**2f**) is a close match for **1**. A preliminary biological investigation revealed that the analog **2** displays superior inhibitory activity towards AChE but inferior activity towards BACE-1, relative to **1**. While the inhibition of both AChE and BACE-1 is potentially desirable for the purpose of treating Alzheimer’s disease, it should be noted that problems have been encountered in clinical trials with BACE-1 as the target [36], suggesting that the analog **2** might still have relevance in the context of an Alzheimer’s treatment. More generally, our finding that the *threo*-difluoroalkane motif is sometimes but not always an effective surrogate for *E*-alkenes suggests that this bioisosteric switch could be exploited more widely in medicinal chemistry as a means of increasing the selectivity of a lead compound towards its desired target.

## Supporting Information

File 1 Synthetic procedures, characterization data for novel compounds and copies of spectra; photostability assessment, conformational analysis of compound **2**, and biological assays.
