# Supplementary material for: Vicinal difluorination as a C=C surrogate: an analog of piperine with enhanced solubility, photostability, and acetylcholinesterase inhibitory activity
Source: Beilstein J Org Chem. 2020 Oct 28;16:2663–70. doi: 10.3762/bjoc.16.216 (PMC7607426; doi:10.3762/bjoc.16.216)
Supplement: File 1 — Synthetic procedures, characterization data for novel compounds and copies of spectra; photostability assessment, conformational analysis of compound 2, and biological assays. [file Beilstein_J_Org_Chem-16-2663-s001.pdf]

BEILSTEIN JOURNAL OF ORGANIC CHEMISTRY

Supporting Information

for

Vicinal difluorination as a C=C surrogate: an analog of piperine with enhanced solubility, photostability, and acetylcholinesterase inhibitory activity

Yuvixza Lizarme-Salas, Alexandra Daryl Ariawan, Ranjala Ratnayake, Hendrik Luesch, Angela Finch and Luke Hunter

Beilstein J. Org. Chem. **2020,** *16,* 2663–2670. doi:10.3762/bjoc.16.216

Synthetic procedures, characterization data for novel compounds and copies of spectra; photostability assessment, conformational analysis of compound 2, and biological assays

License and Terms: This is a supporting information file under the terms of the Creative Commons Attribution License (https://creativecommons.org/licenses/by/4.0). Please note that the reuse, redistribution and reproduction in particular requires that the authors and source are credited.
The license is subject to the *Beilstein Journal of Organic Chemistry* terms and conditions: (https://www.beilstein-journals.org/bjoc)

Table of contents

1. Reagents and instrumentation S2
2. Synthetic procedures and characterisation data for novel compounds S3
3. Attempted oxidation of difluorinated alcohol **13** S12
4. Measurement of enantiopurity S13
5. Reproductions of NMR spectra S14
6. Photostability assessment S34
7. Conformational analysis of compound **2** S36
8. Biological assays S40
9. References S42

1. Reagents and instrumentation

Reactions were performed in oven-dried glassware at room temperature and under a nitrogen atmosphere with magnetic stirring unless stated otherwise. All commercial reagents were of synthetic grade and used as received. Dry solvents were obtained by passage through an alumina column, and/or by storage over 4 Å molecular sieves. Reactions were monitored by thin layer chromatography (TLC) using Merck aluminum-backed silica gel 60 F_{254} (0.2mm) TLC plates. TLC product spots were visualised either under short-wave UV light (254 nm) or by staining with $KMnO_4$ and heating. Flash chromatography was performed using Davisil 40–63 mesh silica gel, and eluents are stated as volume-to-volume ratios. Melting points were determined using an OptiMelt melting point apparatus MPA100. IR spectra were recorded using a Cary 630 Fourier Transform Infrared Spectrometer equipped with attenuated total reflectance (ATR) with a diamond crystal inset. NMR spectra were obtained using Bruker Advance III 300, 400 and 600 MHz instruments at 300 K. Residual solvent peaks were used as an internal reference to calibrate ^{1}H and ^{13}C spectra. Splitting patterns are represented by s = singlet, d = doublet, br s = broad singlet, dm = doublet of multiplets (typically for CHF protons where $^{2}J_{HF}$ is easy to determine while the others are difficult). When necessary to aid in the peak assignment, 2D experiments including COSY, HMBC, and HSQC were acquired. Coupling constants from complex spectra were obtained by Daisy simulation using the Bruker TopSpin software. HRMS results were acquired at the UNSW Bioanalytical Mass Spectrometry Facility using an Orbitrap LCQ XP Plus ion trap MS operating in positive ion mode using electrospray ionisation (ESI). Optical rotations were measured using a Perkin Elmer model 341 polarimeter (λ= 589 nm; *l* = 1 dm and *c* expressed in grams per 100 mL).

2. Synthetic procedures and characterisation data for novel compounds

(2*R*,3*R*)-5-(Benzo[*d*][1,3]dioxol-5-yl)-2,3-difluoro-1-(piperidin-1-yl)pentan-1-one (2)

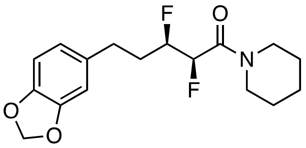

A solution of compound **20** (12.5 mg, 0.048 mmol), 1-hydroxybenzotriazole monohydrate (80%, 8.20 mg, 0.048 mmol) and *N*,*N′*-diisopropylcarbodiimide (0.048 mmol) in dry DMF (1.0 mL) was stirred at rt for 15 min. Piperidine (4.31 mg, 0.508 mmol) was added, and the mixture was stirred at rt under nitrogen for 22 h. The mixture was evaporated under reduced pressure and the residue was dissolved in ethyl acetate (30 mL). The organic layer was washed with citric acid (15 mL), sodium bicarbonate (15 mL), and brine (15 mL), then dried (Na_2SO_4) and concentrated onto silica. The crude product was subjected to flash chromatography eluting with 5:95→40:60 ethyl acetate/hexane to afford the title compound as yellow oil (8.1 mg, 51%); **[α]D** +35.7 (*c* 0.385, $CHCl_3$); **IR** (DCM) ν_{max} (cm^{-1}) 3257, 2940, 1643, 1490, 1445, 1368, 1246, 1189, 1101, 1038; **^{1}H NMR** (600 MHz, $CDCl_3$) δ 6.73 (d, *J* = 7.9 Hz, 1H, ArH), 6.68 (d, *J* = 1.6 Hz, 1H, ArH), 6.65 (dd, *J* = 1.6, 7.9 Hz, 1H, ArH), 5.92 (s, 1H, OCH_2O), 5.04 (ddd, *J* = 3.5, 25.2, 49.0, 1H, CHFCHFCO), 4.83 (dddt, *J* = 3.5, 9.8, 22.1, 47.8, 1H, CHFCHFCO), 3.64–3.47 (m, 4H, CH_2NCH_2), 2.79 (ddd, *J* = 4.9, 8.9, 13.9 Hz, 1H, $CHHCH_2CHF$), 2.66 (ddd, *J* = 8.2, 8.2, 13.9 Hz, 1H, $CHHCH_2CHF$), 2.13 (ddd, *J* = 4.3, 14.3, 27.6 Hz, 1H, $CH_2CHHCHF$), 1.90 (ddddd, *J* = 3.5, 8.2, 9.2, 14.3, 35.1 Hz, 1H, $CH_2CHHCHF$), 1.67–1.53 (m, 6H, N–$CH_2CH_2CH_2$), **^{13}C{^{1}H} NMR** (151 MHz, $CDCl_3$) δ 164.6 (dd, 5.3, 18.7 Hz), 147.9, 146.1, 134.3, 121.5, 109.1, 108.5, 101.0, 92.0 (dd, *J* = 20.1, 196.8 Hz), 91.2 (dd, *J* = 19.4, 177.6 Hz), 46.6 (d, *J* = 12.2 Hz), 44.2, 32.5 (dd, *J* = 4.2, 21.1 Hz), 30.8 (d, *J* = 4.1 Hz) 26.7, 25.8, 24.6; **^{19}F NMR (**377 MHz, $CDCl_3$), δ –194.2 (s, 1F, CH_2CH_2CHF), –195.8 (s, 1F, $CH_2CHFCHF$); **^{19}F{^{1}H} NMR** (377 MHz, $CDCl_3$), δ –194.2 (d, *J* = 12.3 Hz, CH_2CH_2CHF), –195.8 (d, *J* = 12.3 Hz, $CH_2CHFCHF$); **HRMS** (ESI, +ve) $C_{17}H_{21}O_3F_2N_1Na^+$ [MNa^+] requires *m/z* 348.1382, found 348.1372.

(2*S*,3*S*)-5-(Benzo[*d*][1,3]dioxol-5-yl)-1-(benzyloxy)pentane-2,3-diol (8)

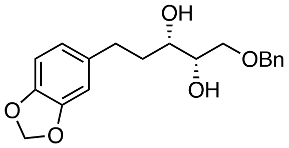

Step 1: To a suspension of sodium hydride (60% dispersion in mineral oil, 48.2 mg, 1.26 mmol) in dried THF (2 mL) at 0 ºC was added allylic alcohol **7**[1] (129 mg, 0.627 mmol), and the reaction mixture was stirred at 0 ºC under nitrogen for 1 hour. Benzyl bromide (323 mg, 1.89 mmol) was added, and the mixture was stirred at

rt for 24 h. Mixture was quenched with saturated sodium bicarbonate and extracted with ethyl acetate (5 × 50 mL), and the combined organic layers were dried ($MgSO_4$) and concentrated onto silica. The residue was subjected to column chromatography, eluting with 1:4 ethyl acetate/hexane, to afford the corresponding benzyl ether as clear oil (106 mg, 57%); **IR** (DCM) ν_{max} (cm^{-1}) 2922, 2851, 2359, 1732, 1606, 1487, 1440, 1360, 1243, 1186, 1096, 1039; **^{1}H NMR** (400 MHz, $CDCl_3$) δ 7.38–7.28 (m, 5H, ArH), 6.72 (d, *J* = 7.9 Hz, 1H. ArH), 6.68 (d, *J* = 1.5 Hz, 1H. ArH), 6.63 (dd, *J* = 1.5, 7.9 Hz, 1H. ArH), 5.91 (s, 2H, $OC\underline{H}_2O$), 5.73 (dt, *J* = 6.3, 15.4 Hz, 1H, $CH{=}C\underline{H}CH_2OBn$), 5.62 (dt, *J* = 6.4, 15.4 Hz, 1H, $C\underline{H}{=}CHCH_2OBn$), 4.48 (app s, 2H, $PhC\underline{H}_2$), 3.97 (s, 1H, $C\underline{H}HOBn$), 3.96 (s, 1H, $CH\underline{H}OBn$), 2.63 (dd, *J* = 7.2, 8.2 Hz, 2H, $CH_2C\underline{H}_2$), 2.34 (dd, *J* = 7.2, 7.7 Hz, 2H, $C\underline{H}_2CH_2$); **^{13}C{^{1}H} NMR** (101 MHz, $CDCl_3$) δ 147.6, 145.7, 138.6, 135.7, 133.8, 128.5, 127.9, 127.7, 127.2, 121.2, 109.0, 108.2, 100.9, 72.0, 70.9, 35.4, 34.5; **HRMS** (ESI, +ve) $C_{19}H_{20}O_3Na^+$ [MNa^+] requires *m/z* 319.1305, found 319.1297.

Step 2: A mixture of sodium bicarbonate (89.3 mg, 1.06 mmol), potassium carbonate (147 mg, 1.06 mmol), potassium ferricyanide (350 mg, 1.06 mmol), $(DHQ)_2PHAL$ (2.80 mg, 0.004 mmol), *tert*-butanol (880 μL), and water (880 μL) was stirred at rt for 30 min, then cooled to 0 ºC. Osmium tetroxide (0.5% solution in water, 0.54 mL, 0.01 mmol) and methanesulfonamide (33.6 mg, 0.354 mmol) were added, and the reaction mixture was stirred at 0 ºC for 1 h. The benzyl ether synthesised above (105 mg, 0.354 mmol) was added, and the mixture was stirred at 0 ºC for 24 h. A second portion of osmium tetroxide (0.5% solution in water, 0.54 mL, 0.01 mmol) was added, and the mixture was stirred for additional 24 h. Sodium sulphite (560 mg, 4.44 mmol) was added, and the reaction was stirred at rt for 1 h. The mixture was extracted with ethyl acetate (4 × 20 mL) and dichloromethane (4 × 25 mL), and the combined organic layers were dried ($MgSO_4$) and concentrated onto silica. The crude product was subjected to flash chromatography eluting with 1:4 ethyl acetate/hexane to give the title compound as a clear waxy solid (78.1 mg, 67%); **$[\alpha]_D$** –26.4 (*c* 1.06, $CHCl_3$); **m.p.** 85.6–85.9 ºC; **IR** (neat) ν_{max} (cm^{-1}) 3286, 3028, 2895, 2863, 2318, 1607, 1360, 1484, 1438, 1360, 1239, 1106, 1036, 1004; **^{1}H NMR** (400 MHz, $CDCl_3$) δ 7.37–7.29 (m, 5H, ArH), 6.72 (d, *J* = 7.7 Hz, 1H, ArH), 6.69 (d, *J* = 1.2 Hz, 1H, ArH), 6.64 (dd, *J* = 1.2, 7.7 Hz, 1H, ArH), 5.91 (s, 2H, $OC\underline{H}_2O$), 4.56 (d, *J* = 11.8 Hz, 1H, $C\underline{H}HPh$), 4.52 (d, *J* = 11.8 Hz, 1H, $CH\underline{H}Ph$), 3.63–3.54 (m, 4H, $C\underline{H}OH\text{-}C\underline{H}OH\text{-}C\underline{H}_2OBn$), 2.74 (ddd, *J* = 5.4, 9.1, 14.5, 1H, $C\underline{H}HCH_2CHOH$), 2.74 (ddd, *J* = 5.4, 9.1, 14.5, 1H, $C\underline{H}HCH_2CHOH$), 2.61 (ddd, *J* = 7.5, 9.1, 13.6, 1H, $CH\underline{H}CH_2CHOH$), 1.83 (ddd, *J* = 5.3, 13.4, 14.1, 1H, $CH_2C\underline{H}HCHOH$), 1.70 (ddd, *J* = 3.4, 6.9, 13.4, 1H, $CH_2C\underline{H}HCHOH$); **^{13}C{^{1}H} NMR** (101 MHz, $CDCl_3$) δ 147.7, 145.7, 137.7, 135.9, 128,7, 128.1, 127.9, 121.3, 109.1, 108.3, 100.9, 72.8, 72.6, 71.4, 35.5, 31.6; **HRMS** (ESI, +ve) $C_{19}H_{22}O_5Na^+$ [MNa^+] requires *m/z* 353.1359, found 353.1359.

(4*S*,5*S*)-4-(2-(Benzo[*d*][1,3]dioxol-5-yl)ethyl)-5-((benzyloxy)methyl)-1,3,2-dioxathiolane 2,2-dioxide (9)

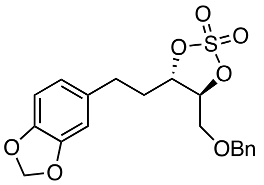

Step 1: Thionyl chloride (0.033 mL, 0.451 mmol) was added to a solution of the diol **8** (74.4 mg, 0.225 mmol) and pyridine (0.055 mL, 0.676 mmol) in dichloromethane (2.6 mL) at 0 °C, and the resulting mixture was stirred at 0 °C for 1 h. Saturated aqueous copper sulphate (20.0 mL) was added, and the reaction was extracted with dichloromethane (3 × 40 mL). The combined organic layers were dried ($MgSO_4$) and concentrated to give the cyclic sulphite as yellow/green oil, which was used without purification for the next step.

Step 2: The crude cyclic sulphite (~0.225 mmol) was dissolved in acetonitrile (1.78 mL) and dichloromethane (1.78 mL), and the solution was cooled to 0 °C. Sodium metaperiodate (96.4 mg, 0.451 mmol), ruthenium chloride hydrate (≈ 10 mg) and water (2.62 mL) were added, and the mixture was stirred at 0 °C for 2 h. Diethyl ether (40 mL) was added, and the mixture was washed with water (2 × 75 mL), saturated aqueous sodium bicarbonate (2 × 75 mL), and brine (2 × 75 mL). The organic layer was dried ($MgSO_4$) and concentrated to give the title compound as dark orange oil (79.5 mg, 90%). **[α]D** –66.7 (*c* 0.150, $CHCl_3$); **IR** (DCM) ν_{max} (cm^{-1}): 2920, 1742, 1489, 1444, 1384, 1208,1100, 1038; **^{1}H NMR** (400 MHz, $CDCl_3$) δ 7.34–7.25 (m, 5H, ArH), 6.74 (d, *J* = 7.9 Hz, 1H, ArH), 6.65 (d, *J* = 1.4 Hz, 1H, ArH), 6.61 (dd, *J* = 1.4, 7.9 Hz, 1H, ArH), 5.94 (s, 2H, $OC\underline{H}_2O$), 4.79 (ddd, *J* = 3.1, 7.6, 9.8 Hz, 1H, $CHSO_4C\underline{H}CH_2OBn$), 4.65 (dt, *J* = 4.7, 7.6 Hz, 1H, $C\underline{H}SO_4CHCH_2OBn$), 4.59 (d, *J* = 11.8 Hz, 1H, C<u>H</u>HPh), 4.54 (d, *J* = 11.8 Hz, 1H, CH<u>H</u>Ph), 3.74 (dd, *J* = 4.6, 11.0 Hz, 1H, C<u>H</u>HOBn), 3.70 (dd, *J* = 4.6, 11.0 Hz, 1H, CH<u>H</u>OBn), 2.80 (ddd, *J* = 4.9, 9.0, 13.9 Hz, 1H, $C\underline{H}HCH_2CHSO_4$), 2.66 (dt, *J* = 8.3, 13.9 Hz, 1H, $C\underline{H}HCH_2CHSO_4$), 2.18 (dddd, *J* = 4.9, 8.5, 9.4, 14.5 Hz, 1H, $C\underline{H}HCH_2CHSO_4$), 1.99 (dddd, *J* = 3.2, 8.0, 8.5, 14.5 Hz, 1H, $CH\underline{H}CH_2CHSO_4$); **^{13}C{^{1}H} NMR** (101 MHz, $CDCl_3$) δ 148.1, 146.4, 136.8, 133.1, 128.8, 128.4, 128.0, 127.9, 121.5, 108.9, 108.6, 101.1, 84.3, 83.7, 74.0, 67.3, 34.5, 31.1; **HRMS** (ESI, +ve) $C_{19}H_{21}O_7S^+$ [MH^+] requires *m/z* 393.1003, found 393.1002.

(2*S*,3*R*)-5-(Benzo[*d*][1,3]dioxol-5-yl)-1-(benzyloxy)-3-fluoropentan-2-ol (10)

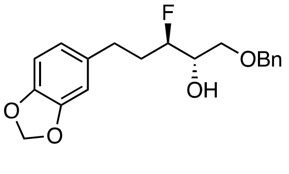

A mixture of tetra-*N*-butylammonium fluoride (1 M solution in THF, 10 mL, 10 mmol) and acetonitrile (60 mL) was dried over 4 Å molecular sieves, then added via cannula to an ice-cold flask containing cyclic sulphate **8** (1.86 g, 4.75 mmol), and the resulting mixture was stirred at rt for 18 h. The mixture was concentrated to give

a clear brown oil. This residue was dissolved in a mixture of sulphuric acid (810 µL), water (148 µL) and tetrahydrofuran (148 mL), and the resulting solution was stirred at rt for 17 h. Brine (150 mL) and water (150 mL) were added, and the mixture was extracted with dichloromethane (5 × 300 mL). The combined organic layers were dried ($MgSO_4$) and concentrated onto silica. The crude product was subjected to flash chromatography eluting with 3:7 ethyl acetate/hexane to give the title compound as yellow liquid (551 mg, 35%); **[α]D** +16.5 (*c* 1.34, $CHCl_3$); **IR** (DCM) ν_{max} (cm^{-1}) 3045, 2920, 2350, 1734, 1488, 1441, 1363, 1263, 1095, 1036; **^{1}H NMR** (400 MHz, $CDCl_3$) δ 7.38–7.29 (m, 5H, ArH), 6.73 (d, *J* = 7.9 Hz, 1H, ArH), 6.69 (d, *J* = 1.3 Hz, 1H, ArH), 6.65 (dd, *J* = 1.3, 7.9 Hz, 1H, ArH), 5.92 (s, 2H, $OC\underline{H}_2O$), 4.58 (d, 1H, *J* = 12.3 Hz, 1H, $C\underline{H}HPh$), 4.54 (d, 1H, *J* = 12.3 Hz, 1H, $CH\underline{H}Ph$), 4.46 (ddd, *J* = 6.4, 12.3, 48.5 Hz, 1H, $CF\underline{H}$), 3.84 (ddd, *J* = 3.6, 6.5, 10.2 Hz, 1H, $C\underline{H}OH$), 3.65 (ddd, *J* = 2.4, 3.3, 9.6 Hz, 1H, $C\underline{H}HOBn$), 3.57 (ddd, *J* = 1.5, 6.4, 9.6 Hz, 1H, $CH\underline{H}OBn$), 2.80 (ddd, *J* = 6.1, 8.3, 14.4 Hz, 1H, $C\underline{H}HCH_2CHF$), 2.63 (dt, *J* = 8.2, 14.4 Hz, 1H, $CH\underline{H}CH_2CHF$), 2.08–1.91 (m, 2H, $CH_2C\underline{H}_2CHF$); **^{13}C{^{1}H} NMR** (101 MHz, $CDCl_3$) δ 147.7, 145.9, 137.8, 135.2, 128.7, 128.7, 127.9, 121.4, 109.1, 108.3, 100.9, 92.5 (d, *J* = 170.9 Hz), 73.7, 71.6 (d, *J* = 24.3 Hz), 70.4 (d, *J* = 5.2 Hz), 33.3 (d, *J* = 20.4 Hz), 30.1 (d, *J* = 3.2 Hz); **^{19}F NMR** (377 MHz, $CDCl_3$) δ –193.6 (s, 1F); **^{19}F{^{1}H} NMR** (377 MHz, $CDCl_3$) δ –193.6; **HRMS** (ESI, +ve) $C_{19}H_{21}FO_4Na^+$ [MNa^+] requires *m/z* 355.1316, found 355.1317.

5-((3*R*,4*R*)-5-(benzyloxy)-3,4-difluoropentyl)benzo[*d*][1,3]dioxole (11)

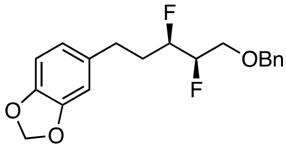

Following a literature protocol [2], a mixture of fluorohydrin **10** (99.4 mg, 0.299 mmol) and a solution of Deoxo-Fluor™ in toluene (2.7 M, 1.1 mL, 3.0 mmol) was stirred at 70 °C in a Teflon vessel for 24 h. The mixture was cooled to 0 °C, diluted with dichloromethane, and concentrated onto silica. The crude product was subjected to flash chromatography eluting with 1:9 ethyl acetate/hexane to give the title compound as a pale yellow oil (25.4 mg, 25%) and the side product **12** (21.2 mg, 23%). Data for **11**: **[α]D** +15.3 (*c* 1.26, $CHCl_3$); **IR** (DCM) ν_{max} (cm^{-1}) 3028, 2923, 2872, 1866, 1607, 1488, 1442,1364, 1246, 1112, 1039; **^{1}H NMR** (400 MHz, $CDCl_3$) δ 7.38–7.28 (m, 5H, ArH), 6.74 (d, *J* = 7.8 Hz, 1H, ArH), 6.68 (d, *J* = 1.2 Hz, 1H, ArH), 6.64 (dd, *J* = 1.2, 7.8 Hz, 1H, ArH), 5.93 (s, 1H, $OC\underline{H}_2O$), 4.75–448 (m, 2H, $C\underline{H}FC\underline{H}F$), 4.57 (app s, 2H, $PhC\underline{H}_2O$), 3.76 (d, *J* = 5.0 Hz, 1H, $CH\underline{H}OBn$), 3.71 (dd, *J* = 2.5, 5.0 Hz, 1H, $C\underline{H}HOBn$), 2.77 (ddd, *J* = 5.0, 9.0, 14.0 Hz, 1H, $CH\underline{H}CH_2CHF$), 2.65 (dt, *J* = 8.1, 14.0 Hz, 1H, $C\underline{H}HCH_2CHF$), 2.11 (dddd, *J* = 5.0, 9.0, 27.8 Hz, 1H, $CH_2C\underline{H}HCHF$), 1.83 (ddddd, *J* = 3.4, 7.4, 14.0, 34.8 Hz, 1H, $CH_2CH\underline{H}CHF$); **^{13}C{^{1}H} NMR** (101 MHz, $CDCl_3$) δ 147.8, 146.0, 137.6, 134.6, , 128.6 ,128.0, 127.9, 121.4, 109.0, 108.4, 101.0, 92.3 (dd, *J* = 19.7, 179.0 Hz), 90.7 (dd, *J* = 19.8,

175.6 Hz), 73.8, 68.5 (dd, *J* = 6.9, 24.8 Hz), 32.3 (dd, *J* = 4.9, 21.1 Hz), 30.8 (d, *J* = 4.4 Hz); **^{19}F NMR (**377 MHz, $CDCl_3$), δ –200.3 (s, 1F, CH_2CH_2CHF), –202.1 (s, 1F, $CH_2CH_2CHFCHF$); **^{19}F{^{1}H} NMR** (377 MHz, $CDCl_3$), δ –200.3 (d, *J* = 10.5 Hz, CH_2CH_2CHF), –202.1 (d, *J* = 10.5 Hz, $CH_2CH_2CHFCHF$); **HRMS** (ESI, +ve) $C_{19}H_{20}O_3F_2Na^+$ [MNa^+] requires *m/z* 357.1273, found 357.1263.

(5*S*,6*R*)-5-((Benzyloxy)methyl)-6-fluoro-5,6,7,8-tetrahydronaphtho[2,3-*d*][1,3]dioxole (12)

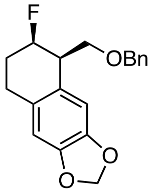

Obtained as a side-product during the synthesis of difluoroalkane **11**. Data for **12**: pale yellow oil; **[α]$_D$** –19.8 (*c* 0.561, $CHCl_3$); **IR** (DCM) ν_{max} (cm^{-1}): 3067, 3033, 2880, 2181, 2091, 1869, 1737, 1485, 1453, 1360, 1330, 1225, 1113, 1092, 1038; **^{1}H NMR** (400 MHz, $CDCl_3$) δ 7.37–7.28 (m, 5H, ArH), 6.84 (s, 1H, ArH), 6.58 (s, 1H, ArH), 5.89 (ddd, *J* = 1.3, 3.9, 12.3 Hz, 1H, OCH_2O), 5.20 (dddd, *J* = 2.5, 3.9, 6.5, 49.2, 1H, CHF), 4.61 (d, *J* = 12.5 Hz, 1H, PhCHHO), 4.58 (d, *J* = 12.5 Hz, 1H, PhCHHO), 3.87 (ddd, *J* = 0.9, 5.7, 9.1 Hz, 1H, CHHOBn), 3.83 (dd, *J* = 6.9, 9.1 Hz, 1H, CHHOBn), 3.15 (ddd, *J* = 6.1, 10.5, 27.4 Hz, 2H, $CHCH_2OBn$), 2.95 (ddd, *J* = 6.9, 9.7, 16.4 Hz, 1H, $CHFCH_2CHH$), 2.66 (dt, *J* = 5.7, 16.4 Hz, 1H, $CHFCH_2CHH$), 2.24 (ddd, *J* = 6.1, 13.6 33.1 Hz, 1H, $CHFCHHCH_2$), 1.89 (ddddd, *J* = 2.5, 6.8, 9.3, 13.6, 34.2 Hz, 1H, $CHFCHHCH_2$); **^{13}C{^{1}H} NMR** (101 MHz, $CDCl_3$) δ 146.3 (d, *J* = 3.1 Hz), 138.4, 129.3, 128.5, 127.8, 127.7, 127.6, 127.5, 108.5, 108.0, 100.9, 89.1 (d, 173.5Hz), 73.5, 71.1 (d, *J* = 7.3 Hz), 42.8 (d, *J* = 19.4 Hz), 27.1 (d, *J* = 20.7 Hz), 25.3 (d, *J* = 7.5 Hz); **^{19}F NMR (**377 MHz, $CDCl_3$), δ –195.1 (s, 1F, CHF); **^{19}F{^{1}H} NMR** (377 MHz, $CDCl_3$), δ –195.1 (m, 1F, CHF); **HRMS** (ESI, +ve) $C_{19}H_{19}O_3FNa^+$ [MNa^+] requires *m/z* 337.1210, found 337.1206.

(2*R*,3*R*)-5-(Benzo[*d*][1,3]dioxol-5-yl)-2,3-difluoropentan-1-ol (13)

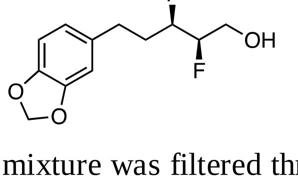

Following a literature protocol [3], compound **11** (28.1 mg, 0.084 mmol) was dissolved in methanol (2 mL), and Pd/C (10%, 24.5 mg) was added to the solution. The mixture was stirred under H_2 atmosphere at rt for 18 h. The mixture was filtered through Celite and washed with methanol. The filtrate was concentrated under reduced pressure to yield the title compound as a sticky white solid (19.8 mg, 97%); **[α]$_D$** +26.3 (*c* 1.00, $CHCl_3$); **IR** (neat) ν_{max} (cm^{-1}) 3338, 2930, 2060, 1997, 1719, 1608, 1491, 1444, 1360, 1247, 1190, 1098, 1039; **^{1}H NMR** (400 MHz, $CDCl_3$) δ 6.74 (d, *J* = 7.9 Hz, 1H, ArH), 6.69 (d, *J* = 1.6 Hz, 1H, ArH), 6.65 (dd, *J* = 1.6, 7.9 Hz, 1H, ArH), 5.93 (s, 1H, OCH_2O), 4.59 (dddt, *J* = 3.5, 9.6, 22.8,

47.5 Hz, CHFCH$_2$OH), 4.52 (ddddd, *J* = 3.5, 3.5, 6.7, 23.0, 48.0 Hz, CHFCHFCH$_2$OH), 3.91 (ddd, *J* = 6.7, 12.6, 19.1 Hz, 1H, CHHOH), 3.82 (ddd, *J* = 3.5, 12.6, 25.6 Hz, 1H, CHHOH), 2.78 (ddd, *J* = 5.2, 8.6, 13.8 Hz, 1H, CHHCH$_2$CHF), 2.66 (dt, *J* = 8.2, 13.8 Hz, 1H, CHHCH$_2$CHF), 2.13 (ddd, *J* = 4.8, 9.1, 13.8, 23.0 Hz, 1H, CH$_2$CHHCHF), 1.82 (m, 1H, CH$_2$CHHCHF); **^{13}C{^{1}H} NMR** (101 MHz, CDCl$_3$) δ 147.9, 146.1, 134.5, 121.4, 109.0, 108.5, 101.0, 93.7 (dd, *J* = 19.7, 177.4 Hz), 90.6 (dd, *J* = 19.7, 175.1 Hz), 61.8 (dd, *J* = 7.2, 23.9 Hz), 32.4 (dd, *J* = 4.7, 21.2 Hz), 30.8 (d, *J* = 4.4 Hz); **^{19}FNMR** (377 MHz, CDCl$_3$), δ –200.1 (d, *J* = 11.4 Hz, CH$_2$CH$_2$CHF), –204.9 (d, *J* = 11.4 Hz, CH$_2$CH$_2$CHFCHF); **^{19}F{^{1}H} NMR (**377 MHz, CDCl$_3$), δ –200.1 (m, 1F, CH$_2$CH$_2$CHF), –204.9 (m, 1F, CH$_2$CH$_2$CHFCHF); **HRMS** (ESI, +ve) $C_{12}H_{14}F_2O_3Na^+$ [MNa$^+$] requires *m/z* 267.0803, found 267.0808.

Ethyl (2*R*,3*S*)-5-(benzo[*d*][1,3]dioxol-5-yl)-2,3-dihydroxypentanoate (16)

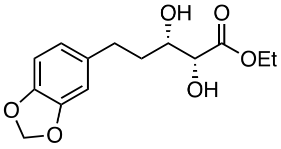

A mixture of AD-mix-α (1.4 g), *tert*-butanol (1.5 mL), and water (1.5 mL) was stirred at rt for 30 min. The mixture then was cooled to 0 ºC, and ester **15** (0.24 g, 0.97 mmol) was added, followed by the addition methanesulphonamide (95 mg, 0.97 mmol), and the reaction mixture was stirred at 0 ºC for 48 h. The reaction was quenched with addition sodium sulphite (1.5 g, 12 mmol), and stirred at rt for 1 h. The mixture was extracted with ethyl acetate (4 × 100 mL) and dichloromethane (4 × 100 mL), and the combined organic layers were dried ($MgSO_4$) and concentrated onto silica. The residue was subjected to flash chromatography eluting with 3:7 ethyl acetate/hexane to give the title compound as white waxy solid (167 mg, 61%); **[α]$_D$** –24.5 (*c* 0.388, CHCl$_3$); **m.p.** 82.4–82.7 ºC; **IR** (neat) ν_{max} (cm^{-1}) 3453, 3350, 2903, 2779, 1844, 1727, 1384, 1502, 1439, 1332, 1292, 1238, 1190, 1110, 1068, 1036; **^{1}H NMR** (400 MHz, CDCl$_3$) δ 6.72 (d, *J* = 7.9 Hz, 1H, ArH), 6.70 (d, *J* = 1.5 Hz, 1H, ArH), 6.65 (dd, *J* = 1.5, 7.9 Hz, 1H, ArH), 5.91 (s, 2H, OCH$_2$O), 4.27 (ddd, *J* = 2.1, 7.1, 13.9 Hz, 2H, CH$_2$CH$_3$), 4.08 (d, *J* = 1.8 Hz, 1H, CHOHC=O), 3.89 (ddd, *J* = 1.8, 4.7, 8.3 Hz, 1H, CHOHCHOH), 2.74 (ddd, *J* = 5.6, 9.2, 14.0 Hz, 1H, PhCHHCH$_2$), 2.63 (ddd, *J* = 7.2, 8.8, 14.0 Hz, 1H, PhCHHCH$_2$), 1.96–1.80 (m, 2H, CH$_2$CH$_2$CHOH), 1.30 (t, *J* = 7.1 Hz, 3H, CH$_2$CH$_3$), **^{13}C{^{1}H} NMR** (101 MHz, CDCl$_3$) 173.6, 147.8, 145.8, 135.5. 121.3, 109.0, 108.3, 100.9, 73.3, 71.8, 62.3, 35.7, 31.8, 14.3; **HRMS** (ESI, +ve) $C_{14}H_{18}O_6Na^+$ [MNa$^+$] requires *m/z* 305.0996, found 305.0991.

Ethyl (4*R*,5*S*)-5-(2-(benzo[*d*][1,3]dioxol-5-yl)ethyl)-1,3,2-dioxathiolane-4-carboxylate 2,2-dioxide (17)

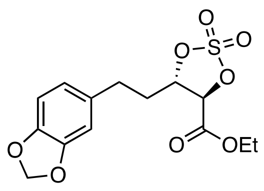

Step 1: Thionyl chloride (110 μL, 1.52 mmol) was added to a solution of the diol **16** (212 mg, 0.751 mmol) and pyridine (180 μL, 2.25 mmol) in dichloromethane (8.75 mL) at 0 ºC, and the resulting mixture was stirred at 0 ºC for 5 h. Saturated aqueous copper sulphate (30 mL) was added, and the reaction mixture was extracted with dichloromethane (3 × 70 mL). The combined organic layers were dried ($MgSO_4$) and concentrated to give the corresponding cyclic sulphite as yellow/green oil which was used in the next step without further purification.

Step 2: The crude cycle sulphite (≈ 0.751 mmol) was dissolved in acetonitrile (5.8 mL) and dichloromethane (5.8 mL) and the solution was cooled to 0 ºC. Sodium metaperiodate (313 mg, 0.45 mmol), ruthenium chloride hydrate (≈ 10 mg), and water (8.5 mL) were added, and the mixture was stirred at 0 ºC for 16 h. Diethyl ether (150 mL) was added and the mixture was washed with water (2 × 75 mL), saturated aqueous sodium bicarbonate (2 × 75 mL), and brine (2 × 75 mL). The organic layer was dried with $MgSO_4$ and concentrated to give a mixture of the title compound as dark orange oil (149 mg, 59%) and unreacted cyclic sulphite intermediate (69.8 mg, 31%). Data for **17**: **[α]$_D$** –70.4 (*c* 0.27, $CHCl_3$); **IR** ($CHCl_3$) ν_{max} (cm^{-1}) 2591, 2544, 2361, 2160, 1767, 1491, 1445, 1395, 1208, 1020; **^{1}H NMR** (400 MHz, $CDCl_3$) δ 6.75 (d, *J* = 7.8 Hz, 1H, ArH), 6.67 (d, *J* = 1.4 Hz, 1H, ArH), 6.64 (dd, *J* = 1.4, 7.8 Hz, 1H, ArH), 5.94 (s, 2H, OC$\underline{H}_2$O), 4.90 (ddd, 1H, *J* = 4.0, 7.2, 8.4 Hz, C$\underline{H}$SO$_4$CH), 4.86 (t, 1H, *J* = 7.2 Hz, CHSO$_4$C$\underline{H}$), 4.32 (dd, *J* = 7.2, 14.2 Hz, 2H, OC$\underline{H}_2$CH$_3$), 2.85 (ddd, H, *J* = 5.4, 8.6, 14.0 Hz, 1H, PhC$\underline{H}$HCH$_2$), 2.71 (dt, H, *J* = 8.3, 14.0 Hz, 1H, PhCH$\underline{H}$CH$_2$), 2.35–2.18 (m, 2H, PhCH$_2$C$\underline{H}_2$), 1.32 (t, *J* = 7.2 Hz, 1H, OCH$_2$C$\underline{H}_3$), **^{13}C{^{1}H} NMR** (101 MHz, $CDCl_3$) 164.8, 148.1, 146.5, 132.7, 121.5, 108.9, 108.7, 101.2, 83.1, 79.9, 63.5, 35.1, 30.8, 14.1.

Ethyl (2*S*,3*S*)-5-(benzo[*d*][1,3]dioxol-5-yl)-2-fluoro-3-hydroxypentanoate (18)

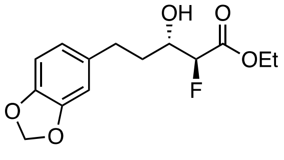

A mixture of tetra-*N*-butylammonium fluoride (1 M solution in THF, 1.5 mL, 1.5 mmol) and acetonitrile (12.5 mL) was dried over 4 Å molecular sieves, then added via cannula to an ice-cold flask containing a mixture of cyclic sulphate **17** and sulphite precursor (≈ 2:1, ≈ 0.64 mmol). The resulting mixture was stirred at rt for 4.5 h. Then, the mixture was concentrated to give a clear brown oil. The residue was dissolved in a

mixture of sulphuric acid (0.12 mL), water (22 µL), and tetrahydrofuran (22 mL), and the resulting solution was stirred at rt for 19 h. Brine (50 mL) and water (50 mL) were added, and the mixture was extracted with dichloromethane (3 × 150 mL). The combined organic layers were dried ($MgSO_4$) and concentrated onto silica. The crude product was subjected to flash chromatography eluting with 1:4 ethyl acetate/hexane to give the title compound as a clear yellow liquid (58 mg, 28%); **[α]D** –31 (*c* 0.86, $CHCl_3$); **IR** ($CHCl_3$) ν_{max} (cm^{-1}) 3512, 2931, 2661, 1740, 1611, 1491, 1444, 1374, 1246, 1095, 1039; **^{1}H NMR** (400 MHz, $CDCl_3$) δ 6.72 (d, *J* = 7.9 Hz, 1H, ArH), 6.68 (s, 1H, ArH), 6.64 (d, *J* = 7.9 Hz, 1H, ArH), 5.91 (s, 2H, $OC\underline{H}_2O$), 4.83 (dd, *J* = 4.0, 48.3 Hz, 1H, $C\underline{H}F$), 4.27 (ddd, *J* = 2.4, 7.0, 14.1 Hz, 2H, $OC\underline{H}_2CH_3$), 3.99 (ddd, *J* = 4.0, 9.6, 17.5 Hz, 1H, $CH_2\underline{H}COH$), 2.79 (ddd, H, *J* = 5.2, 8.8, 14.0 Hz, 1H, $PhC\underline{H}HCH_2$), 2.63 (dt, H, *J* = 8.1, 14.0 Hz, 1H, $PhCH\underline{H}CH_2$), 2.36 (s, 1H, $CH_2HCO\underline{H}$), 1.93–1.76 (m, 2H, $PhCH_2C\underline{H}_2$), 1.29 (t, *J* = 7.1 Hz, 1H, $OCH_2C\underline{H}_3$), **^{13}C{^{1}H} NMR** (101 MHz, $CDCl_3$) 168.1 (d, *J* = 23.9 Hz), 147.8, 145.9, 135.0, 121.3, 109.0, 108.3, 100.9, 91.3 (d, *J* = 187.7 Hz), 70.7 (d, *J* = 21.8 Hz), 61.9, 33.3 (d, *J* = 4.7 Hz), 31.3, 14.2; **^{19}F NMR (**377 MHz, $CDCl_3$), δ –198.9 (s, 1F, $CH\underline{F}$); **^{19}F{^{1}H} NMR** (377 MHz, $CDCl_3$), δ –198.9 (dd, *J* = 17.7, 48.3, 1F, $CH\underline{F}$); **HRMS** (ESI, +ve) $C_{14}H_{17}FO_5Na^+$ [MNa^+] requires *m/z* 307.0952, found 307.0948.

Ethyl (2*R*,3*R*)-5-(benzo[*d*][1,3]dioxol-5-yl)-2,3-difluoropentanoate (19)

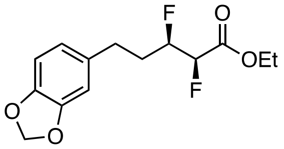

Following a literature protocol [2], a mixture of fluorohydrin **18** (55 mg, 0.19 mmol) and a solution of Deoxo-Fluor™ in toluene (2.7 M, 0.71 mL, 1.9 mmol) was stirred at 70 °C in a Teflon vessel for 20 h. The mixture was cooled to 0 °C, diluted with dichloromethane, and concentrated onto silica. The crude product was subjected to flash chromatography eluting with 1:4 ethyl acetate and hexane to give the title compound as pale yellow oil (29 mg, 52%); **[α]D** +31.3 (*c* 0.560, $CHCl_3$); **IR** (DCM) ν_{max} (cm^{-1}) 2938, 1767, 1491, 1444, 1374, 1299, 1246, 1123, 1038; **^{1}H NMR** (400 MHz, $CDCl_3$) δ 6.75 (d, *J* = 7.8 Hz, 1H, ArH), 6.69 (s, 1H, ArH), 6.65 (d, *J* = 7.8 Hz, 1H, ArH), 5.93 (s, 1H, $OC\underline{H}_2O$), 4.84 (ddddd, *J* = 2.2, 3.7, 9.8, 25.1, 46.2, 1H, $CH_2C\underline{H}FCHF$), 4.83 (ddd, *J* = 2.2, 28.6, 47.3, 1H, $CH_2CHFC\underline{H}F$), 4.33 (d, *J* = 7.1 Hz, 1H, $CH_3C\underline{H}HO$), 4.29 (d, *J* = 7.1 Hz, 1H, $CH_3CH\underline{H}O$), 2.79 (ddd, *J* = 5.1, 8.9, 14.0 Hz, 1H, $CH\underline{H}CH_2CHF$), 2.66 (ddd, *J* = 8.1, 8.1, 14.0 Hz, 1H, $C\underline{H}HCH_2CHF$), 2.23 (ddd, *J* = 5.2, 13.6, 23.9 Hz, 1H, $CH_2C\underline{H}HCHF$), 1.98–1.81 (m, 1H, $CH_2CH\underline{H}CHF$), 1.32 (t, *J* = 7.1, 7.1 Hz, 1H, $OCH_2C\underline{H}_3$); **^{13}C{^{1}H} NMR** (101 MHz, $CDCl_3$) δ 166.8 (dd, 4.5, 24.3 Hz), 147.9, 146.2, 137.6, 134.1, , 128.4, 109.0, 108.5, 101.0, 90.9 (dd, *J* = 20.2, 179.4 Hz), 89.0 (dd, *J* = 20.8, 194.7 Hz), 62.3, 32.1 (dd, *J* = 4.3, 21.4 Hz), 30.8 (d, *J* = 4.8 Hz), 14.2; **^{19}F NMR (**377 MHz, $CDCl_3$), δ –197.1 (s, 1F, $CH_2CH_2CH\underline{F}$), –208.2 (s, 1F, $CH_2CHFCH\underline{F}$ $CH_2CHFCH\underline{F}$); **^{19}F{^{1}H} NMR** (377 MHz,

$CDCl_3$), δ –197.1 (d, *J* = 10.1 Hz, $CH_2CH_2CH\underline{F}$), –208.1 (d, *J* = 10.1 Hz, $CH_2CHFCH\underline{F}$); **HRMS** (ESI, +ve) $C_{14}H_{16}O_4F_2Na^+$ [MNa^+] requires *m/z* 309.0909, found 309.0903.

(2*R*,3*R*)-5-(Benzo[*d*][1,3]dioxol-5-yl)-2,3-difluoropentanoic acid (20)

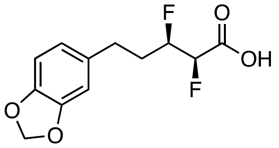

A solution of water (910 µL), HCl (10.2 M, 29 µL, 0.296 mmol), and 1,4-dioxan (0.25 mL) was added to the difluorinated ester **19** (14.1 mg, 0.049). The mixture was heated to 100 °C for 6 h, then diluted with water, and aqueous sodium bicarbonate solution was added until the pH was basic. The mixture was extracted with ethyl acetate (3 × 5 mL). The aqueous layer was acidified with HCl (2 M), then extracted with ethyl acetate (3 × 5 mL). All of the organic layers were combined, dried ($MgSO_4$), and concentrated under reduced pressure to give the title compound as pale yellow oil (12.6 mg, 99%); **[α]D** +27.8 (*c* 0.630, $CHCl_3$); **^{1}H NMR** (400 MHz, $CDCl_3$) δ 6.75 (d, *J* = 7.9 Hz, 1H, ArH), 6.69 (s, 1H, ArH), 6.65 (d, *J* = 7.8 Hz, 1H, ArH), 5.94 (s, 1H, $OC\underline{H_2}O$), 4.89 (ddd, *J* = 1.4, 29.4, 47.0, 1H, $C\underline{H}FCOOH$), 4.87 (ddddd, *J* = 1.4, 3.7, 8.8, 25.2, 45.6, 1H, $C\underline{H}FCHFCOOH$), 2.80 (ddd, *J* = 5.2, 8.8, 14.0 Hz, 1H, $CH\underline{H}CH_2CHF$), 2.67 (ddd, *J* = 8.0, 8.0, 14.0 Hz, 1H, $C\underline{H}HCH_2CHF$), 2.28 (dddd, *J* = 4.5, 8.8, 13.5, 27.2 Hz, 1H, $CH_2C\underline{H}HCHF$), 2.01–1.84 (m, 1H, $CH_2CH\underline{H}CHF$); **^{13}C{^{1}H} NMR** (101 MHz, $CDCl_3$) δ 170.9 (dd, 4.1, 25.7 Hz), 148.0, 146.2, 134, 121.5, 109.0, 108.5, 101.1, 90.7 (dd, *J* = 19.9, 178.8 Hz), 88.5 (dd, *J* = 21.2, 194.0 Hz), 32.0 (dd, *J* = 4.0, 21.3 Hz), 30.7 (d, *J* = 4.7 Hz); **^{19}F NMR (**377 MHz, $CDCl_3$), δ –197.0 (s, 1F, $CH_2CH_2CH\underline{F}$), –208.5 (s, 1F, $CH_2CHFCH\underline{F}$ $CH_2CHFCH\underline{F}$); **^{19}F{^{1}H} NMR** (377 MHz, $CDCl_3$), δ –197.0 (d, *J* = 9.9 Hz, $CH_2CH_2CH\underline{F}$), –208.5 (d, *J* = 10.1 Hz, $CH_2CHFCH\underline{F}$); **HRMS** (ESI, +ve) $C_{12}H_{12}O_4F_2Na^+$ [MNa^+] requires *m/z* 281.0596, found 281.0596.

3. Attempted oxidation of difluorinated alcohol 13

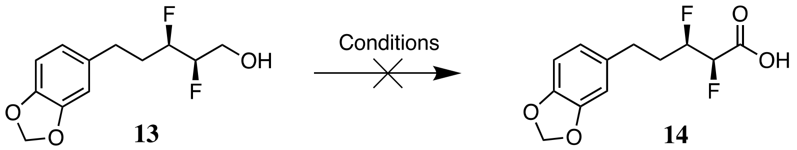

13 **14**

Entry	Conditions	Outcome
1	$NaIO_4$ (4.0 equiv), $RuCl_3$ (0.1 equiv), MeCN, DCM, H_2O, rt, 18 h.	**13** (51%)
2	NaO^tBu (3.0 equiv), benzene, O_2, rt, 24 h.	**13** (95%)
3	PDC (6.0 equiv), DMF, rt, 17 h.	**13** (36%) + multiple other products
4	Jones reagent 4*N*–8*N* (excess), acetone, 0 °C to rt, 2–8h.	**13** (18%) + multiple other products
5	Solution H_5IO_6/CrO_3 (0.44M, 2.9 equiv) MeCN/H_2O (3/1), 3–6h rt, then 50 °C.	**13** (77%) + multiple other products

4. Measurement of enantiopurity

Mosher ester analysis[4]

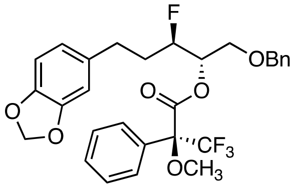

Alcohol **10** (0.006 mmol) and either (*S*)- or (*R*)-Mosher acid (0.030 mmol) were dissolved in dry DCM (350 μL). *N,N'*-Dicyclohexylcarbodiimide (0.020 mmol) was added, followed by 4-dimethylaminopyridine (0.020 mmol), and the mixture was stirred at rt until the reaction was complete as observed by TLC (typically 24 h). The mixture was filtered and concentrated under reduced pressure. The residue was analysed by $^{19}F\{^{1}H\}$ NMR, focusing on and integrating signals in the CHF region.

Chiral HPLC analysis

The enantiomeric purity of **2** was assessed via analytical HPLC using a Chiralcel OD-H column and PDA detector (300 nm), with a solvent system of propanol/hexane (5:95). Two peaks were observed, with retention times of 16.9 min and 17.6 min (see below).

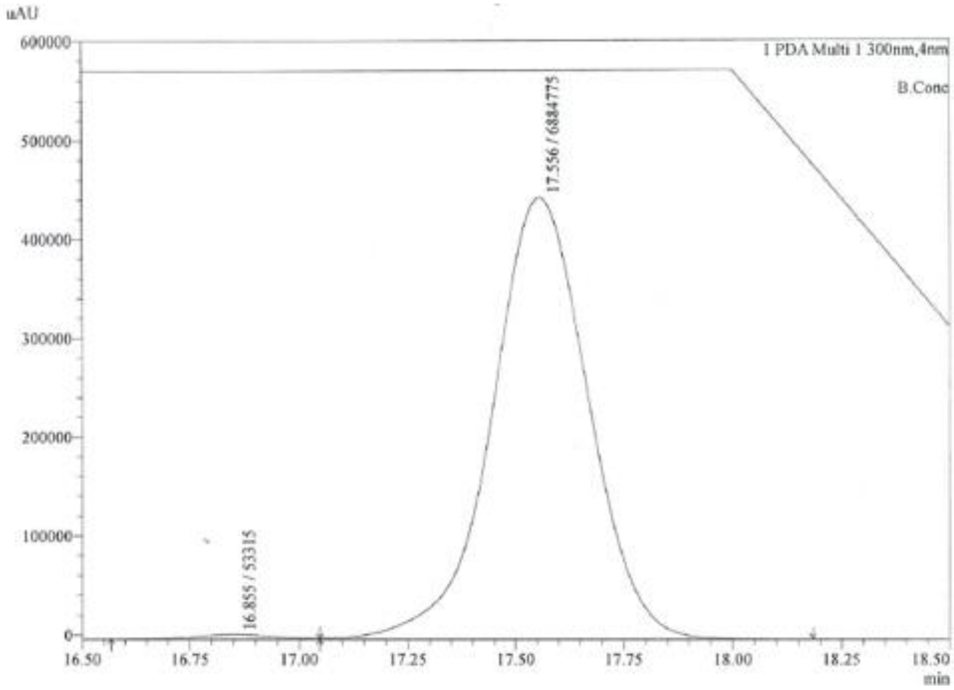

5. Reproductions of NMR spectra

^{1}H NMR (600 MHz, $CDCl_3$) of **2**

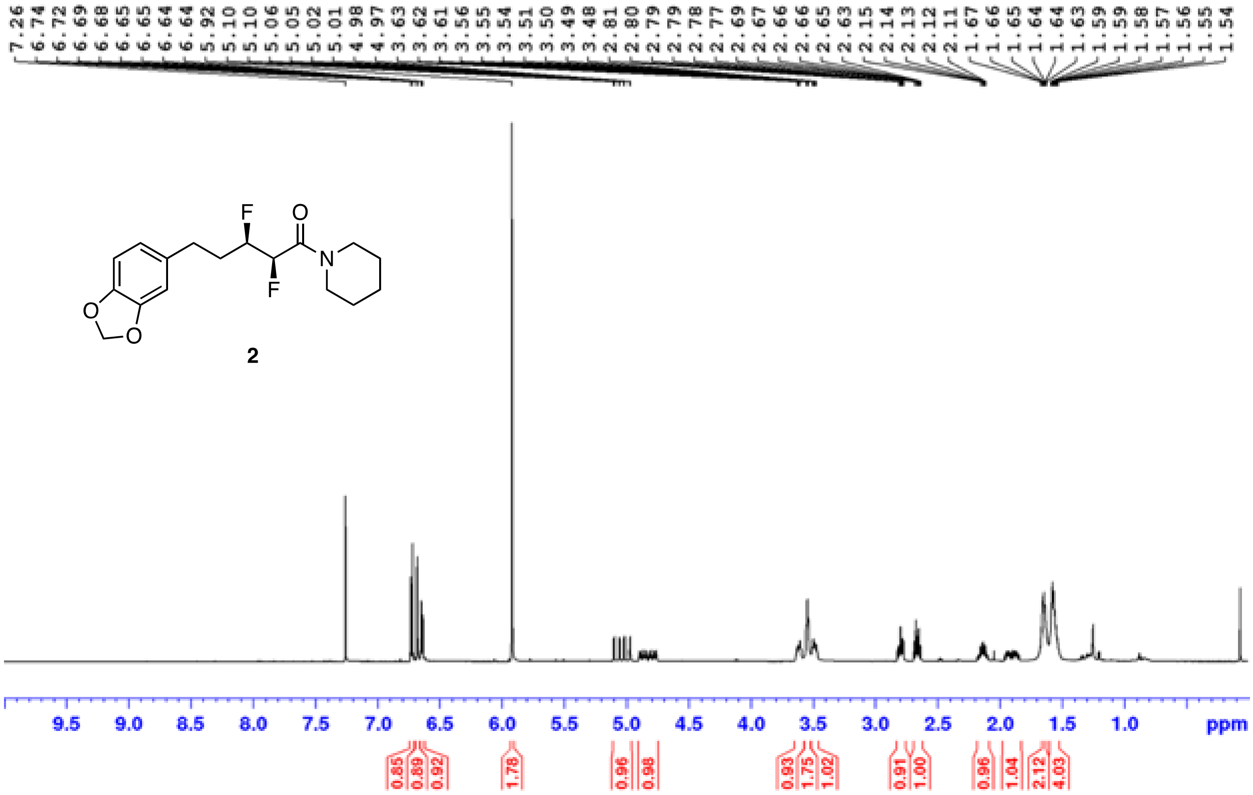

^{13}C{^{1}H} NMR (151 MHz, $CDCl_3$) of **2**

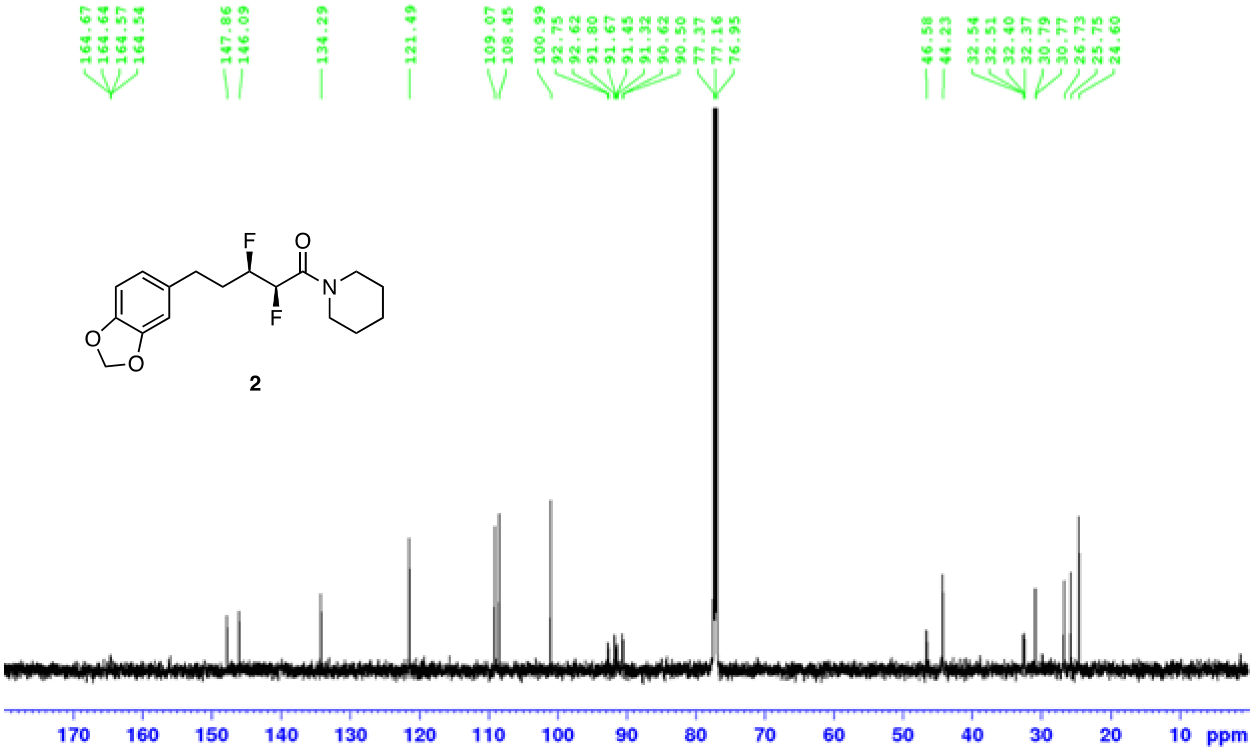

^{19}F NMR (565MHz, $CDCl_3$) of **2**

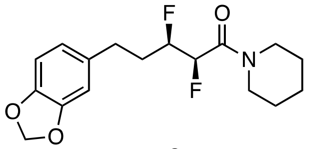

2

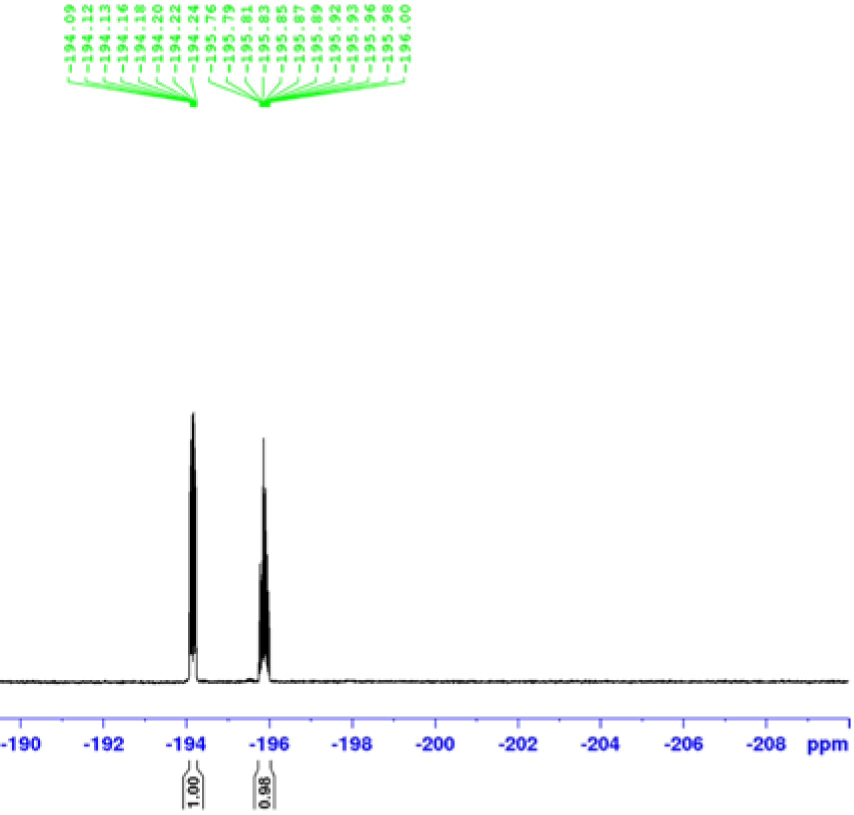

^{19}F {^{1}H} NMR (565 MHz, $CDCl_3$) of **2**

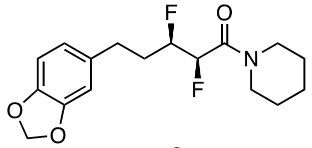

2

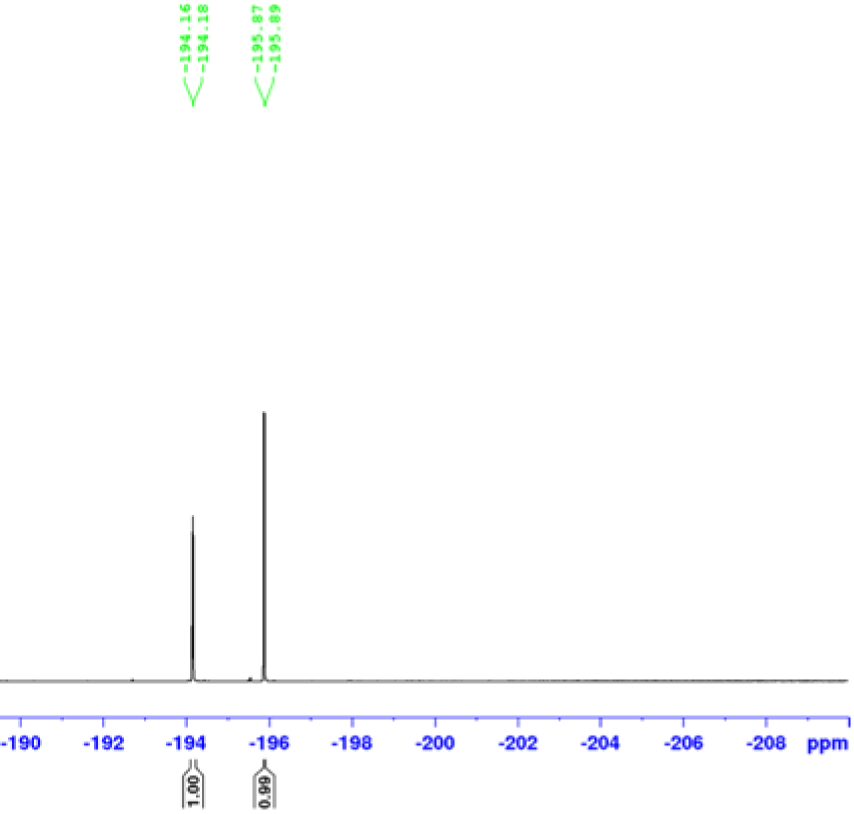

^{1}H NMR (400 MHz, $CDCl_3$) of **8**

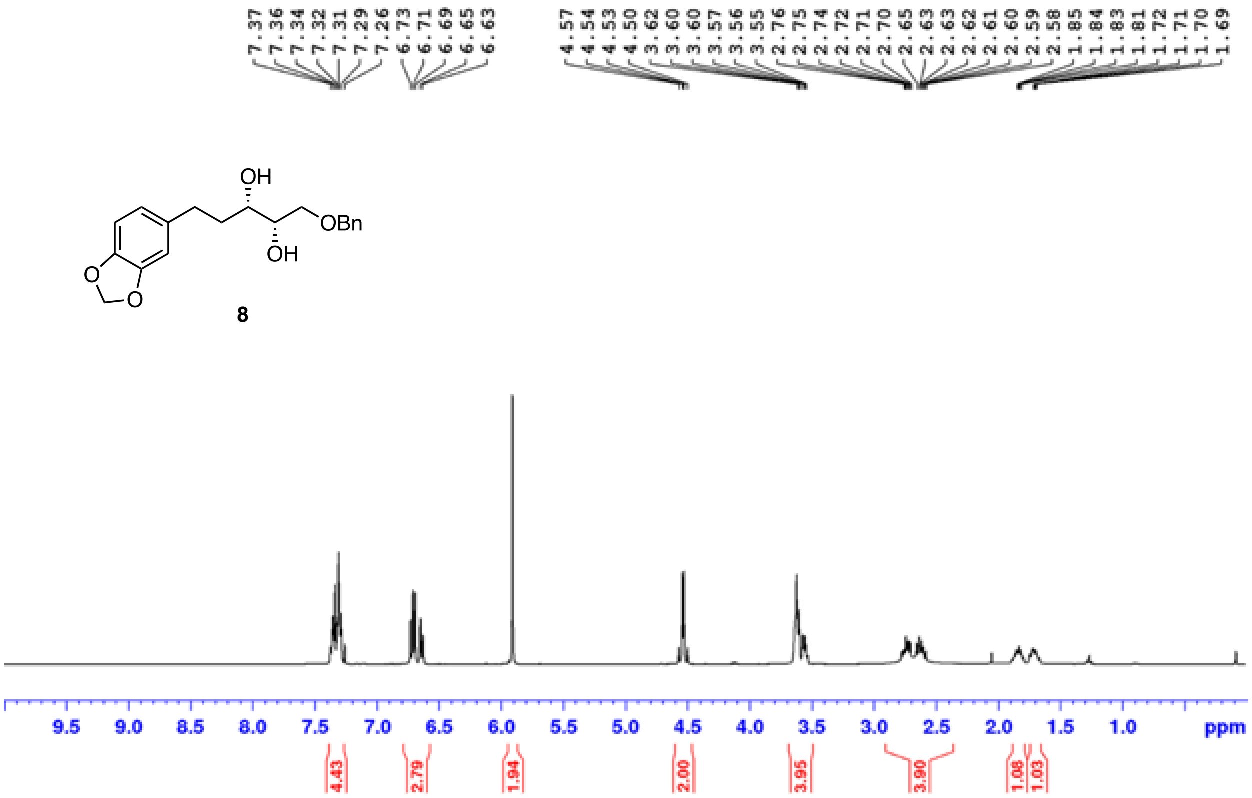

^{13}C{^{1}H} NMR (101 MHz, $CDCl_3$) of **8**

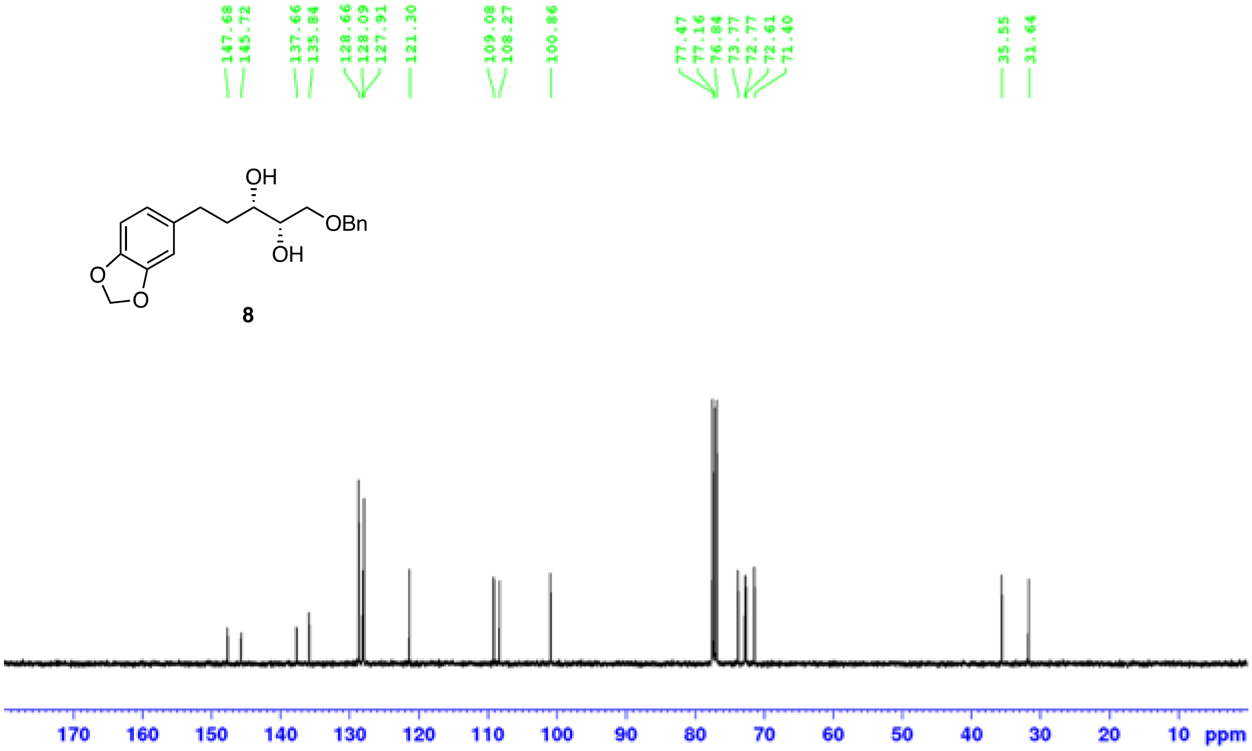

^{1}H NMR (400 MHz, $CDCl_3$) of **9**

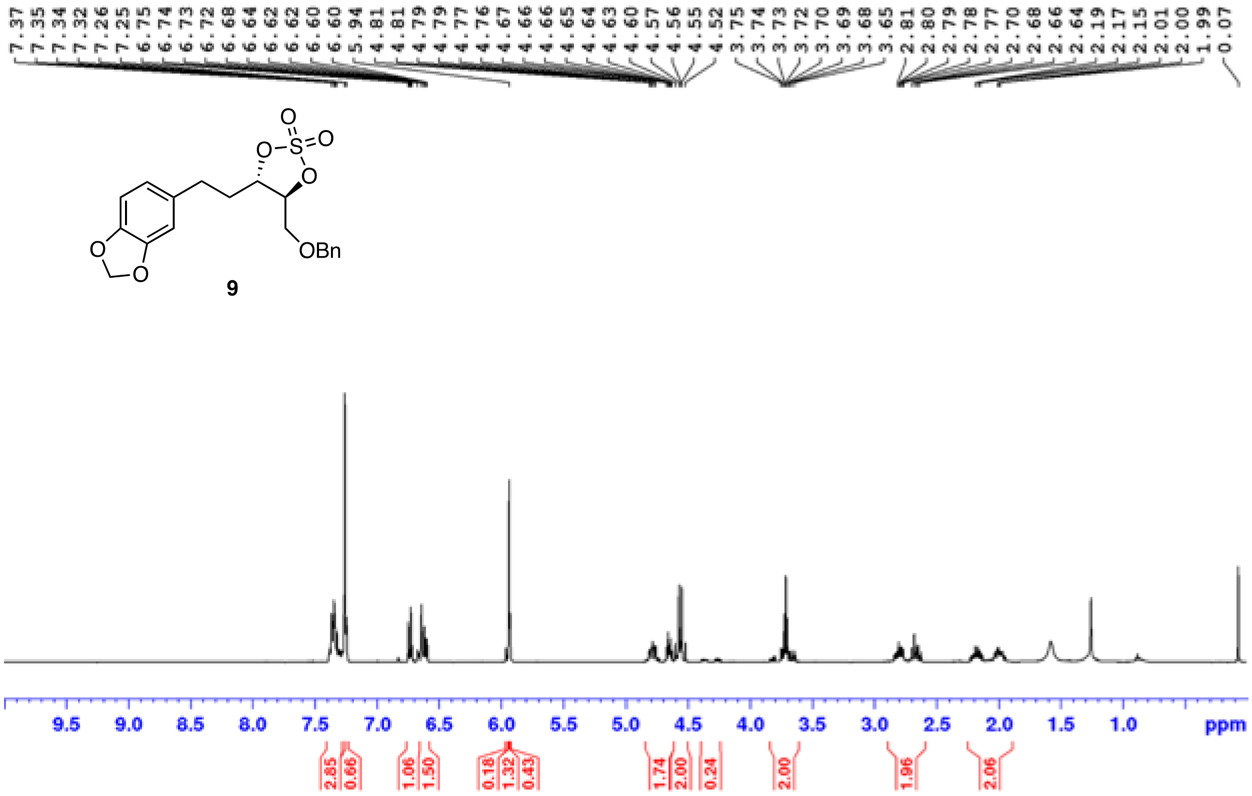

$^{13}C\{^{1}H\}$ NMR (101 MHz, $CDCl_3$) of **9**

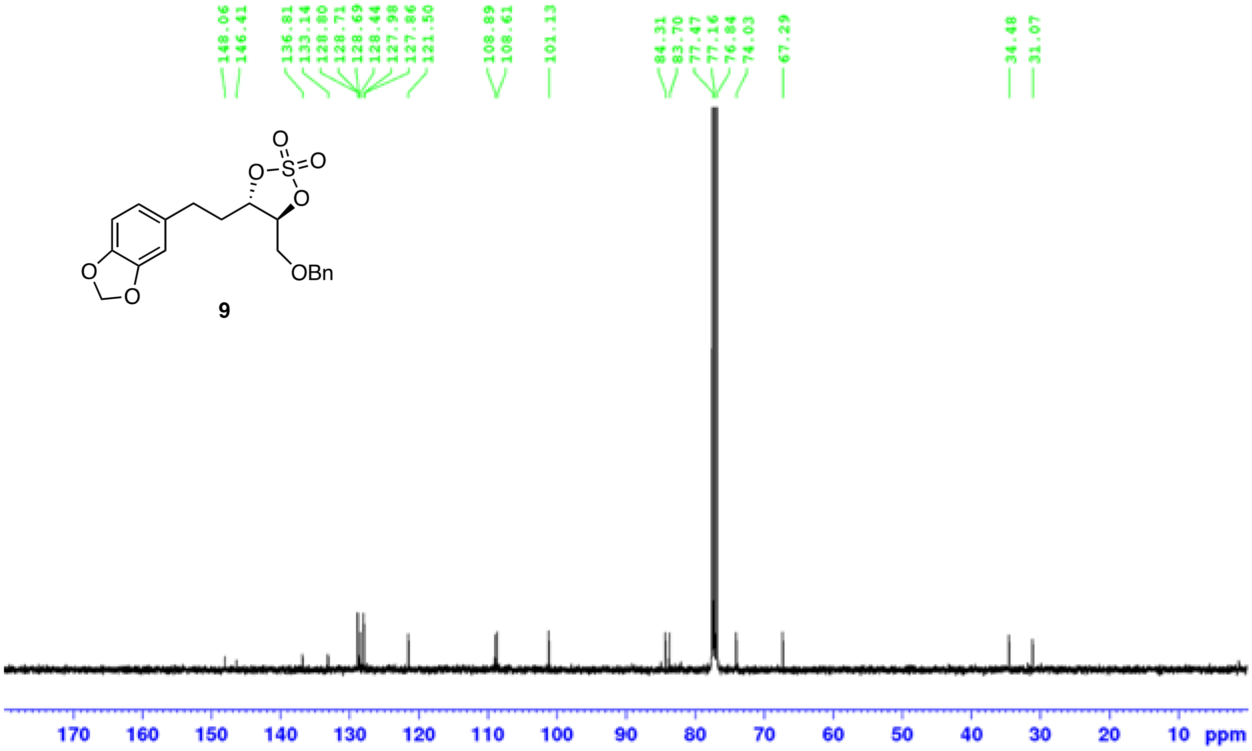

¹H NMR (400 MHz, $CDCl_3$) of **10**

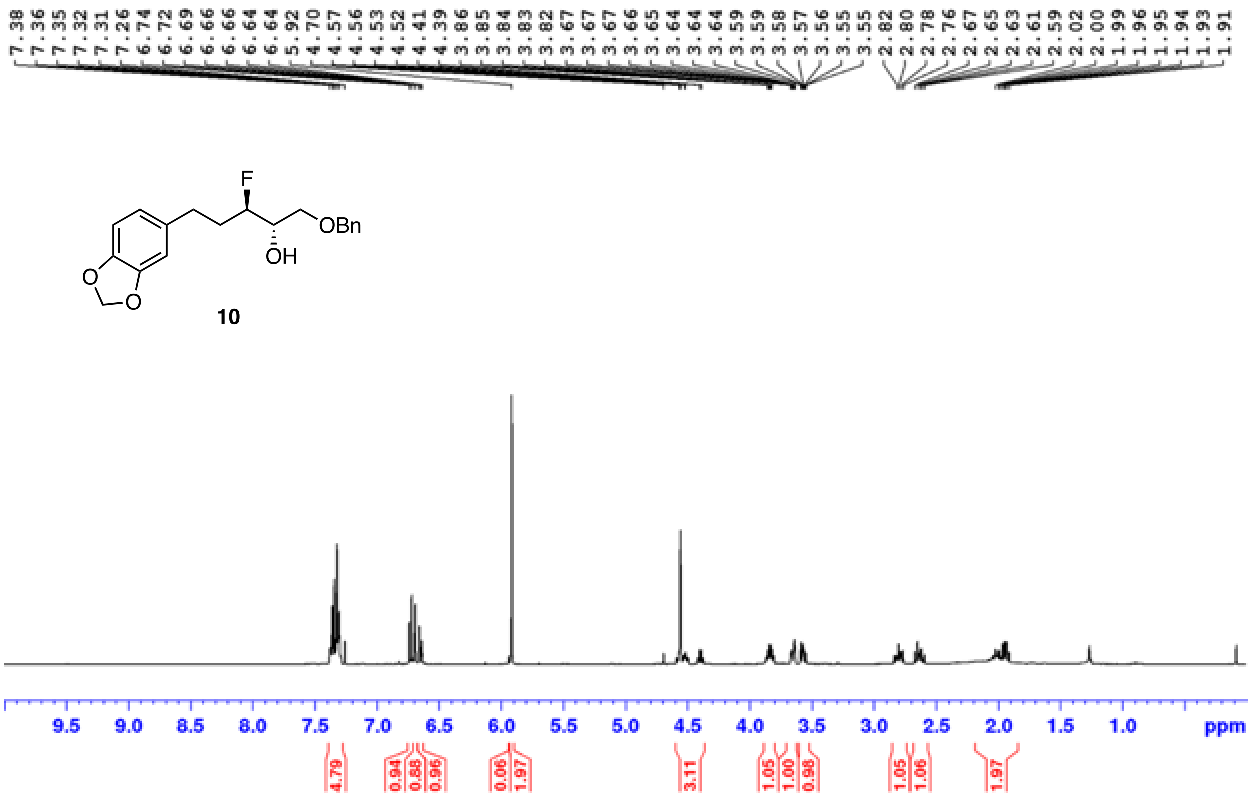

¹³C{¹H} NMR (101 MHz, $CDCl_3$) of **10**

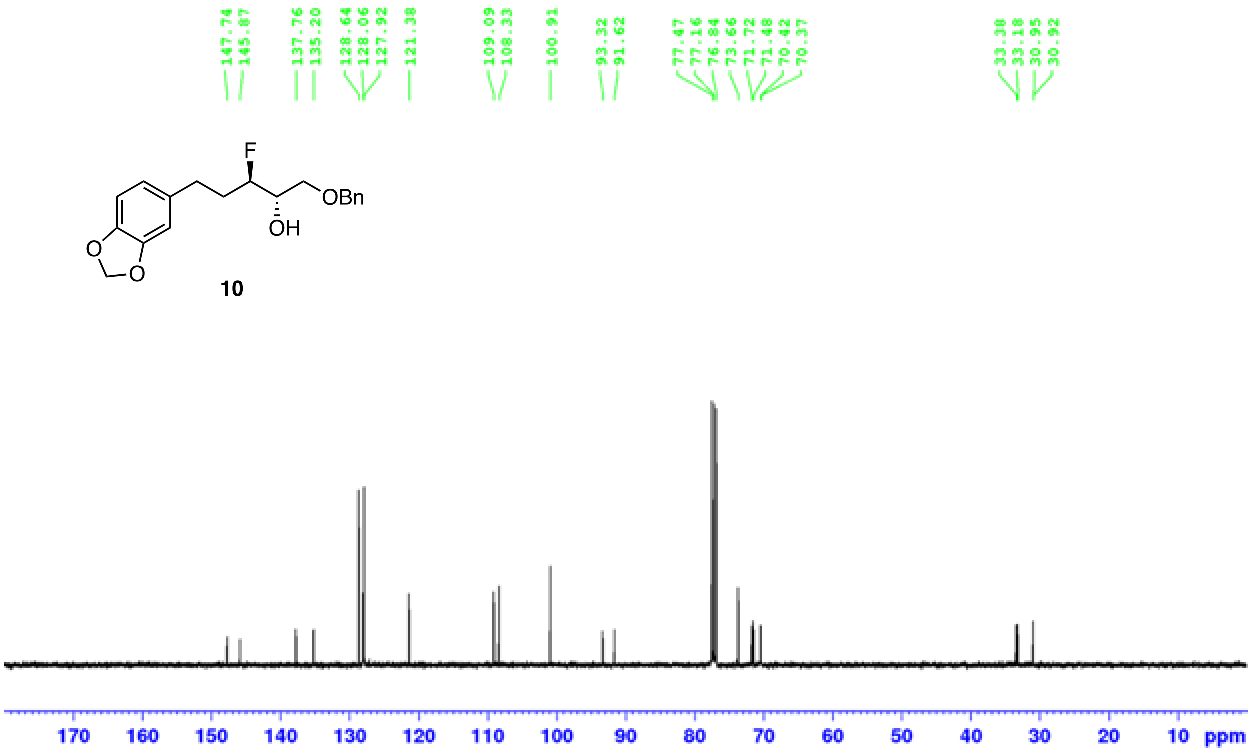

^{19}F NMR (377 MHz, $CDCl_3$) of **10**

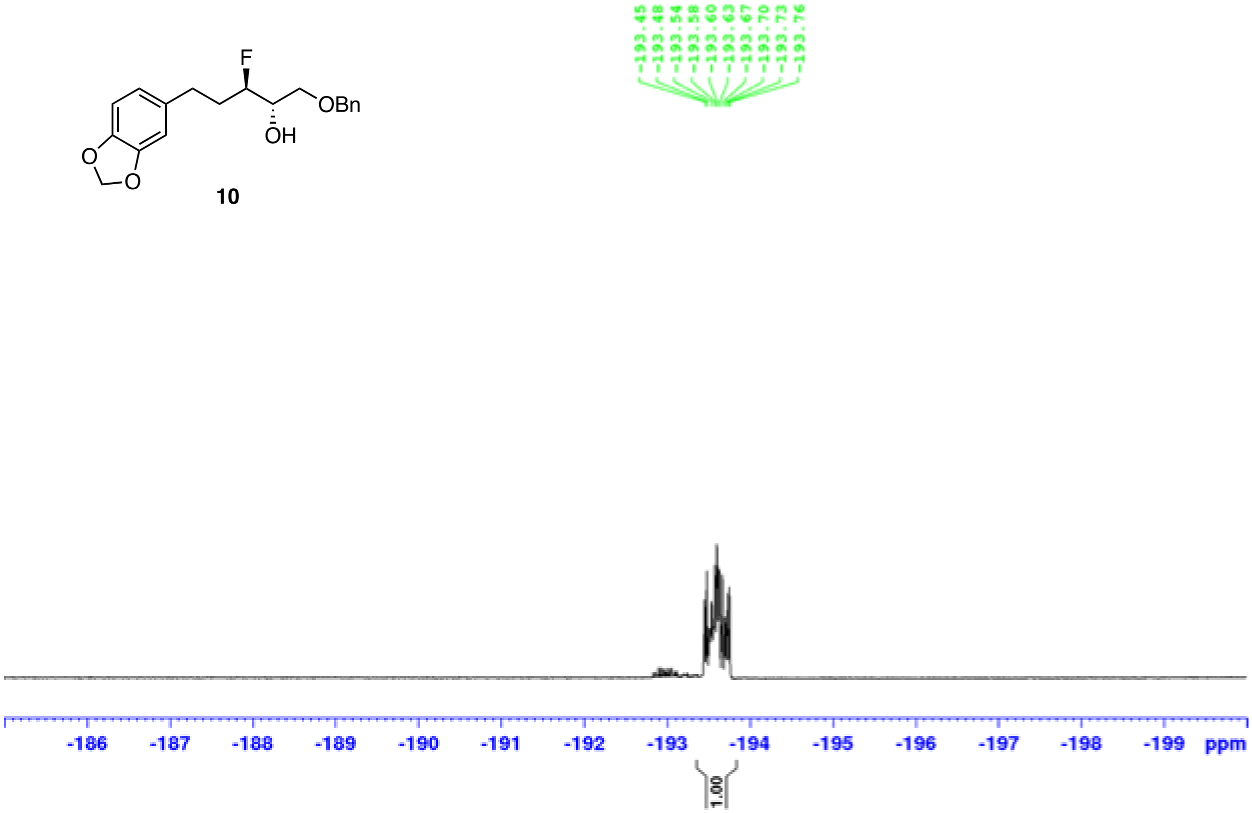

^{19}F{^{1}H} NMR (377 MHz, $CDCl_3$) of **10**

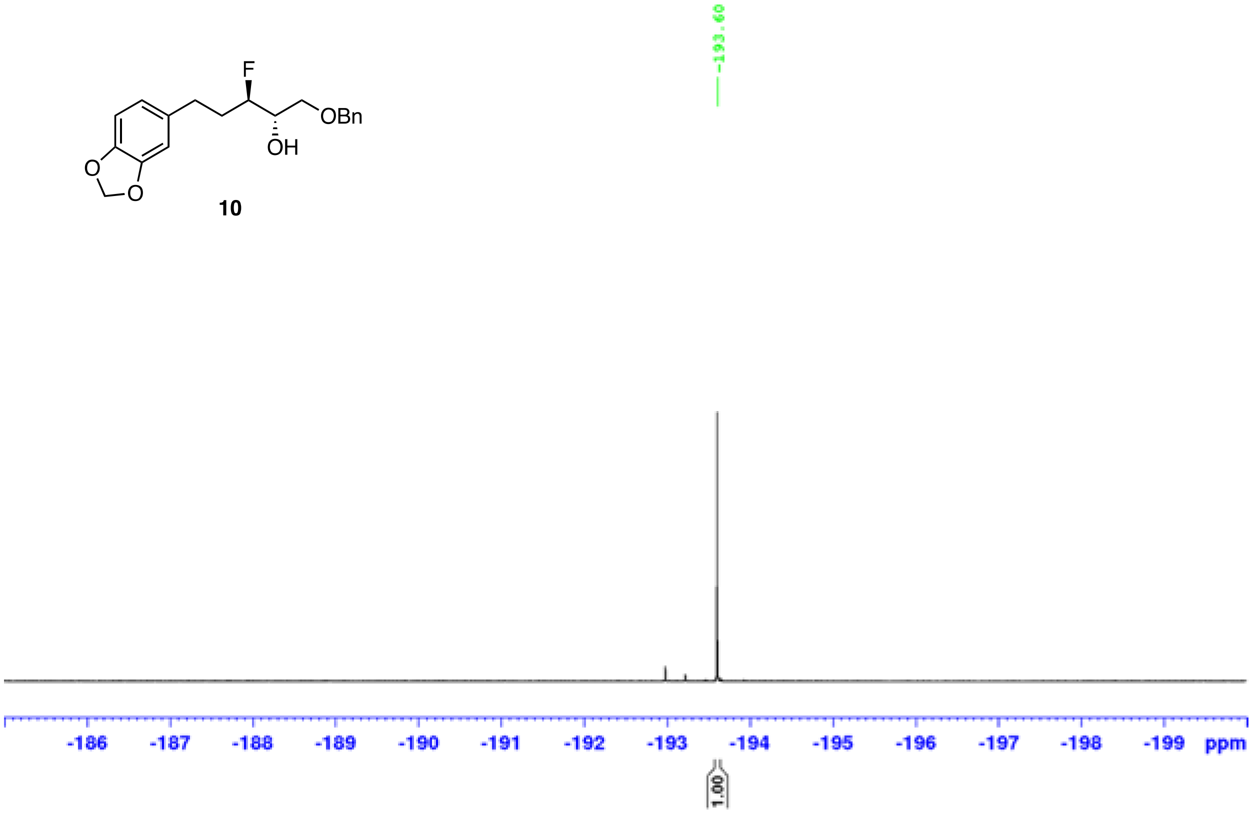

^{1}H NMR (400 MHz, $CDCl_3$) of **11**

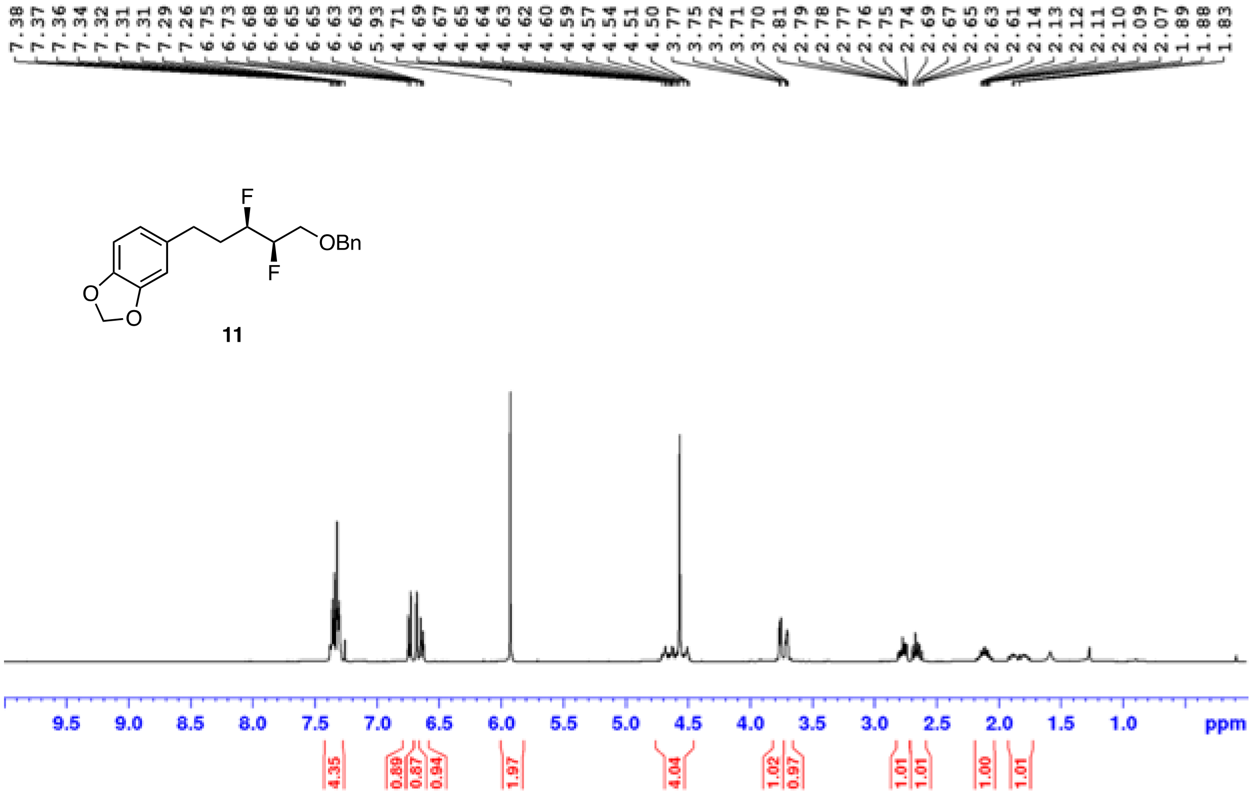

^{13}C{^{1}H} NMR (101 MHz, $CDCl_3$) of **11**

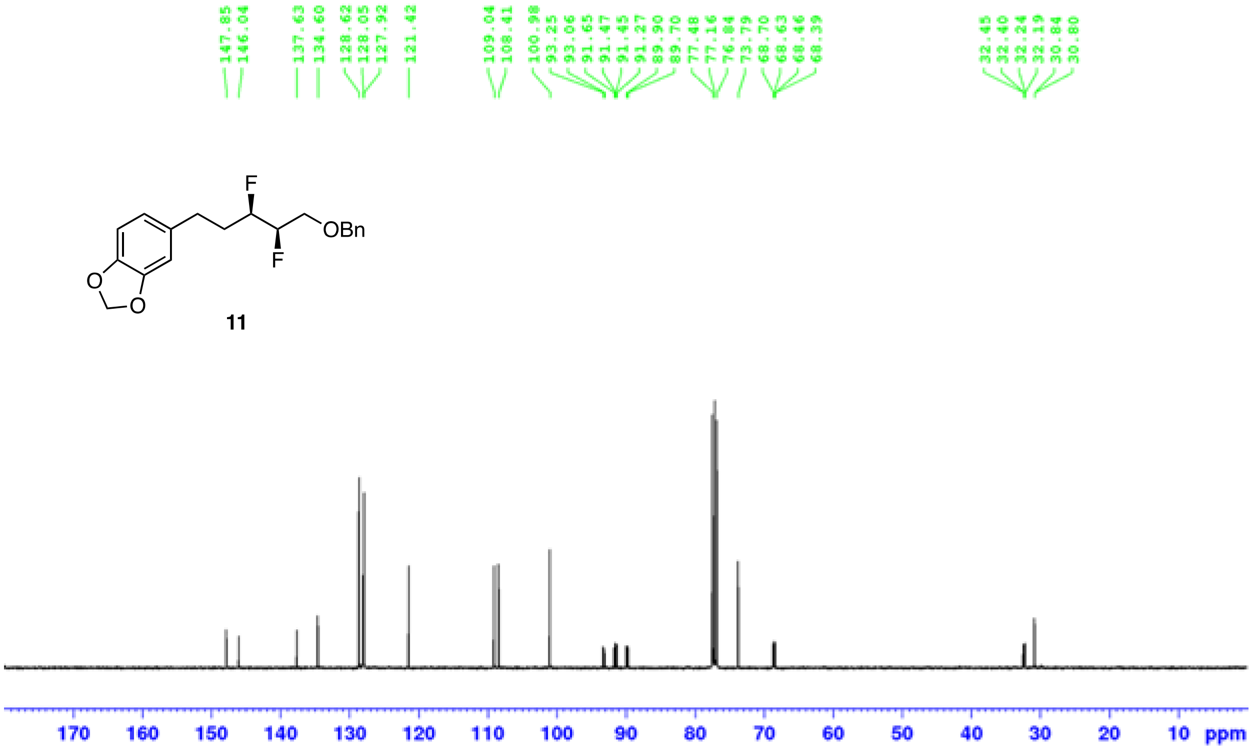

^{19}**F NMR** (377 MHz, CDCl$_3$) of **11**

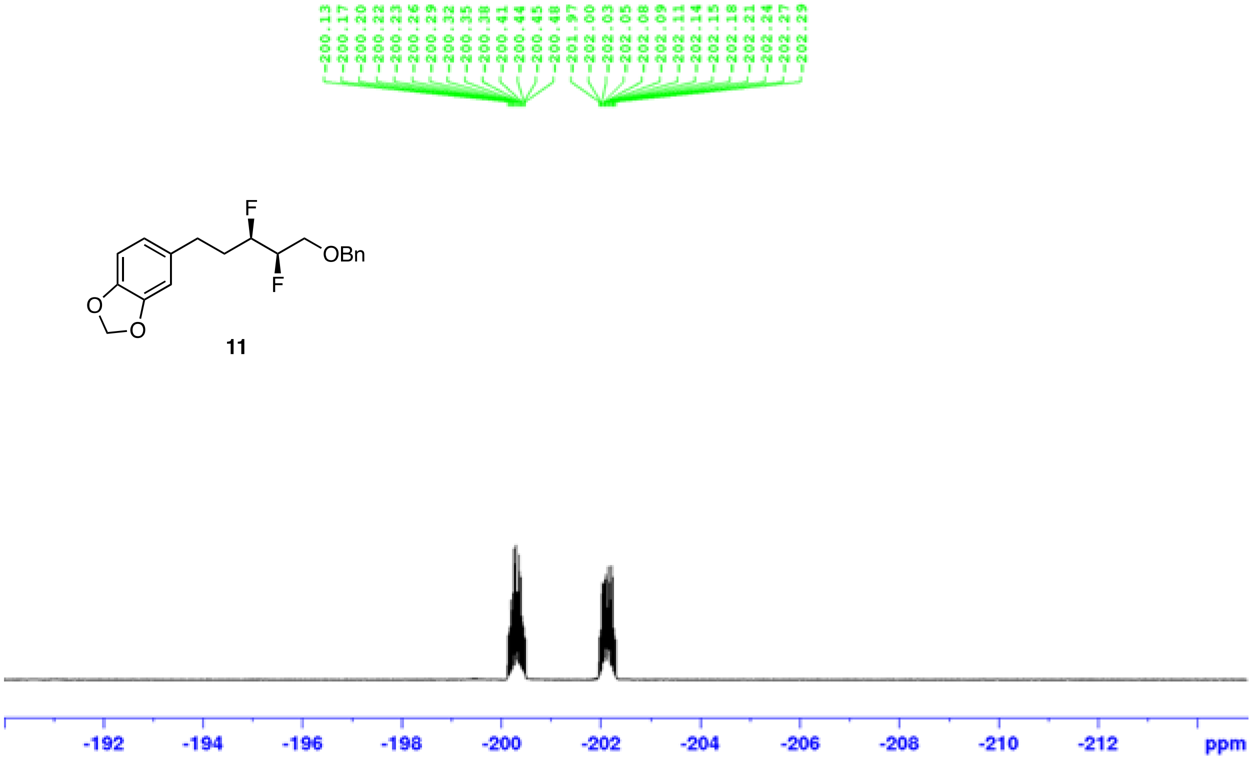

^{19}**F{**^{1}**H} NMR** (377 MHz, CDCl$_3$) of **11**

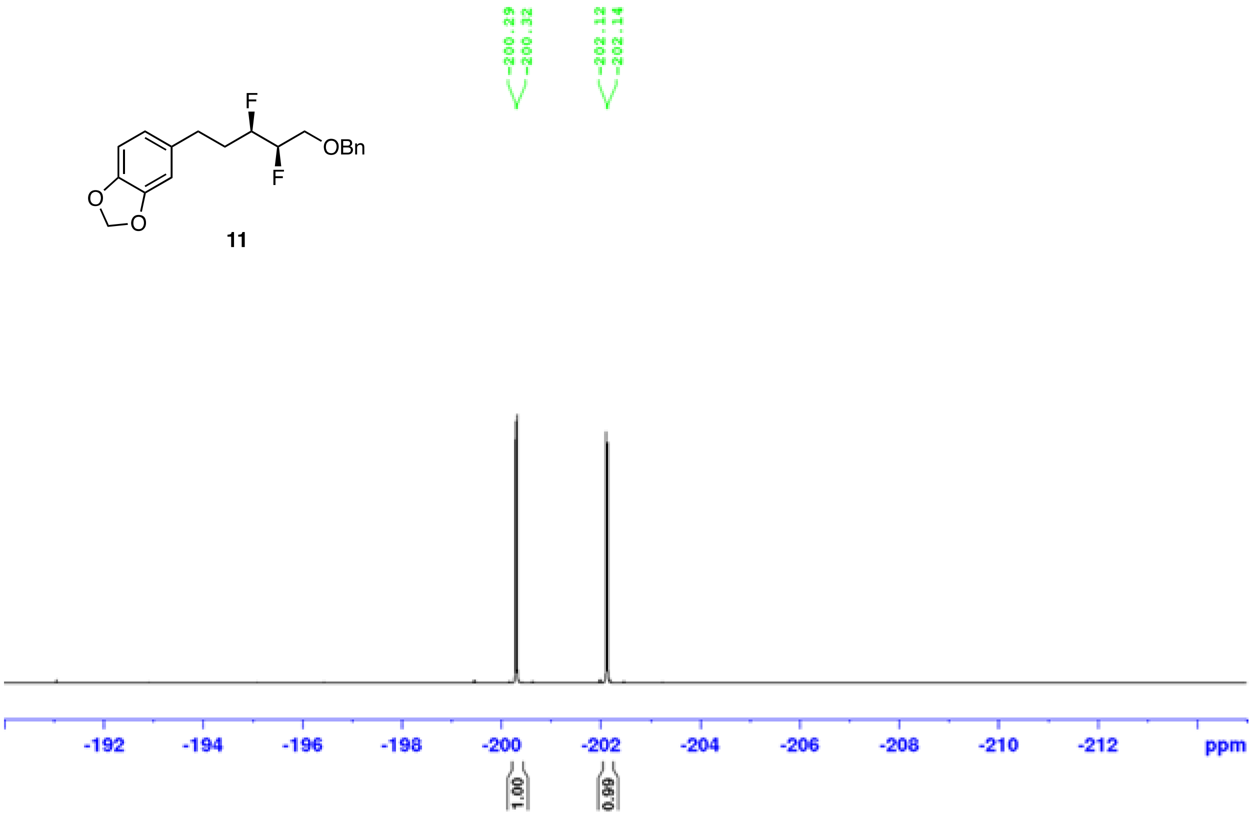

[1]**H NMR** (400 MHz, $CDCl_3$) of **12**

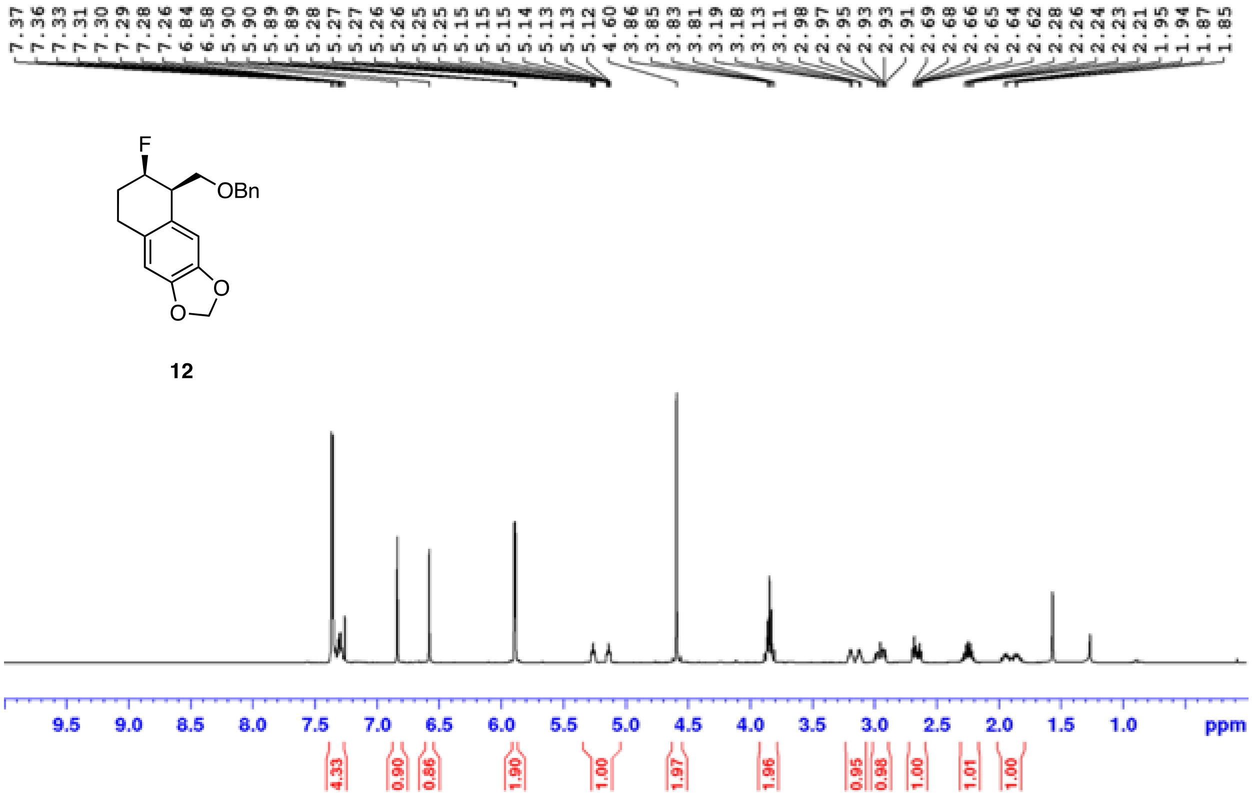

[13]**C{^{1}H} NMR** (101 MHz, $CDCl_3$) of **12**

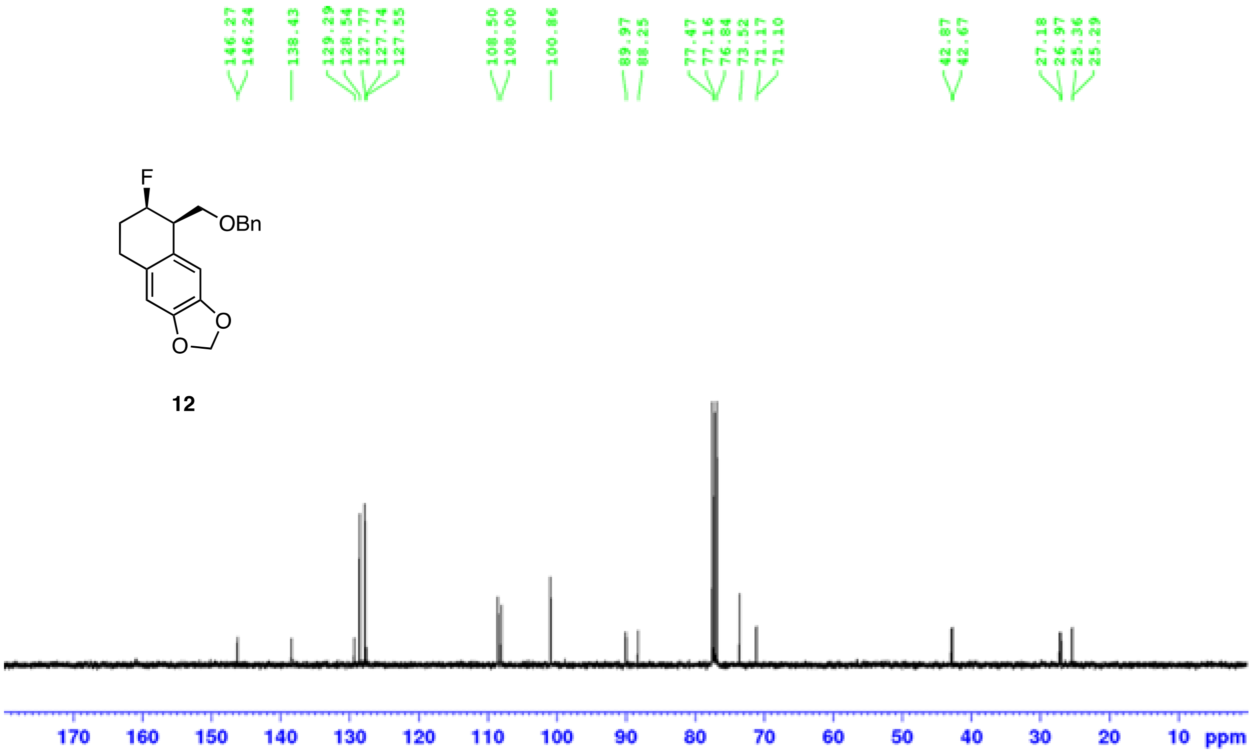

^{19}F NMR (377 MHz, $CDCl_3$) of **12**

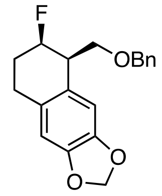

12

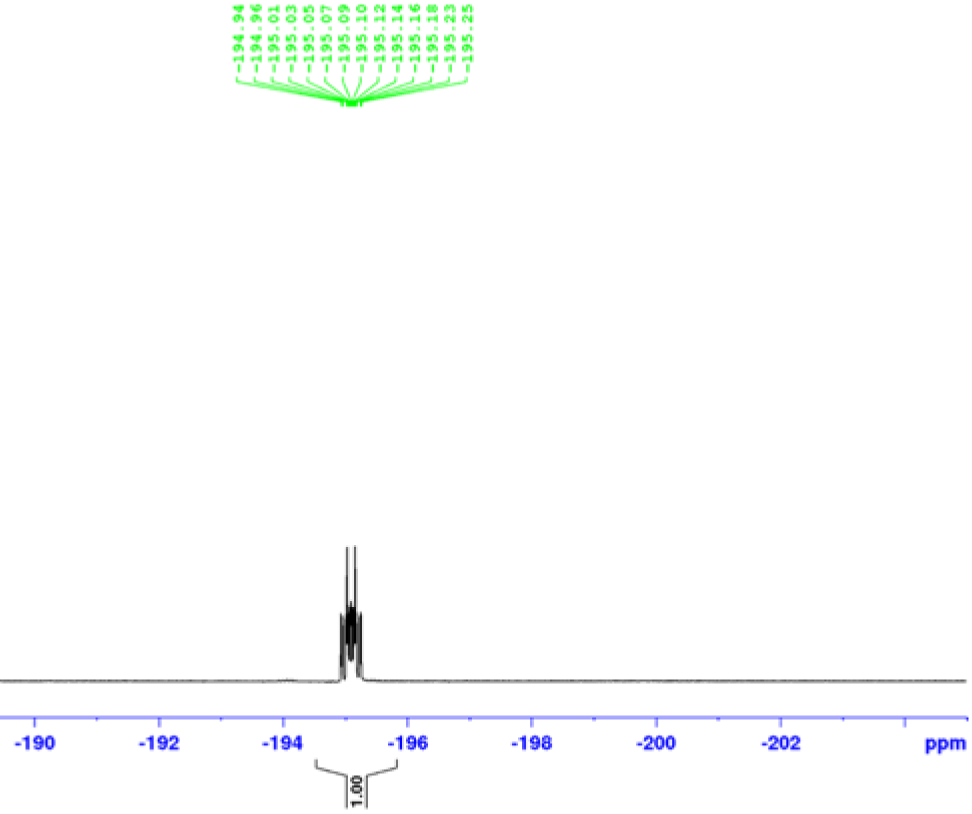

^{19}F{^{1}H} NMR (377 MHz, $CDCl_3$) of **12**

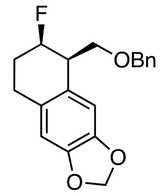

12

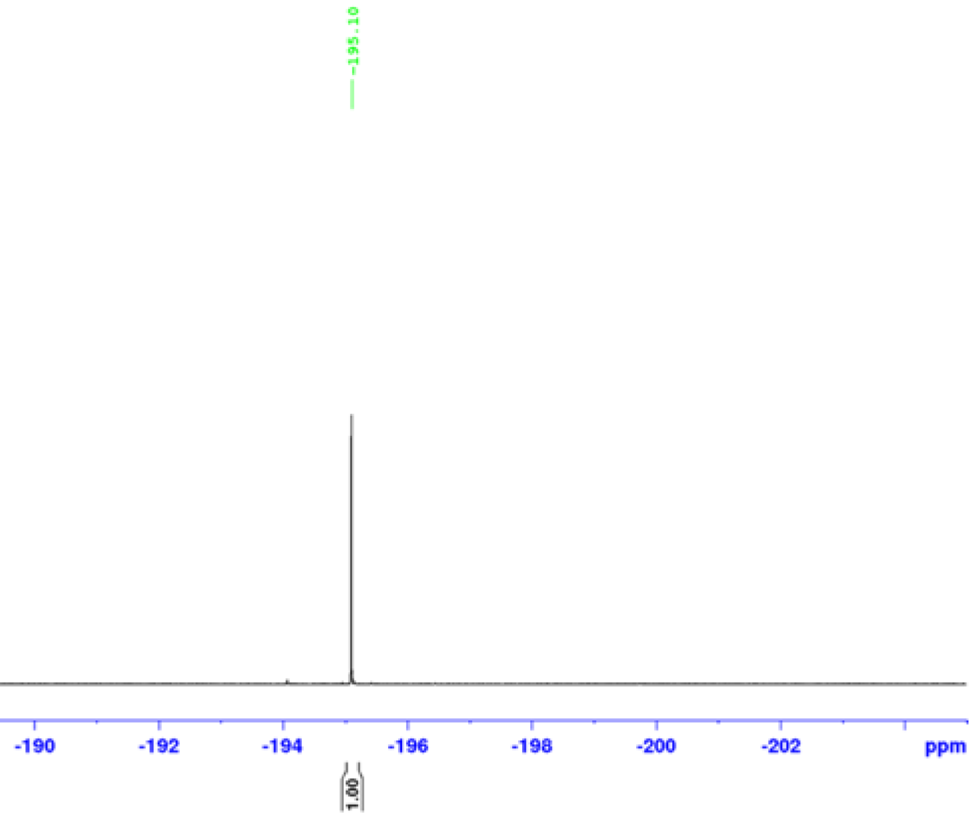

^{1}H NMR (400 MHz, $CDCl_3$) of **13**

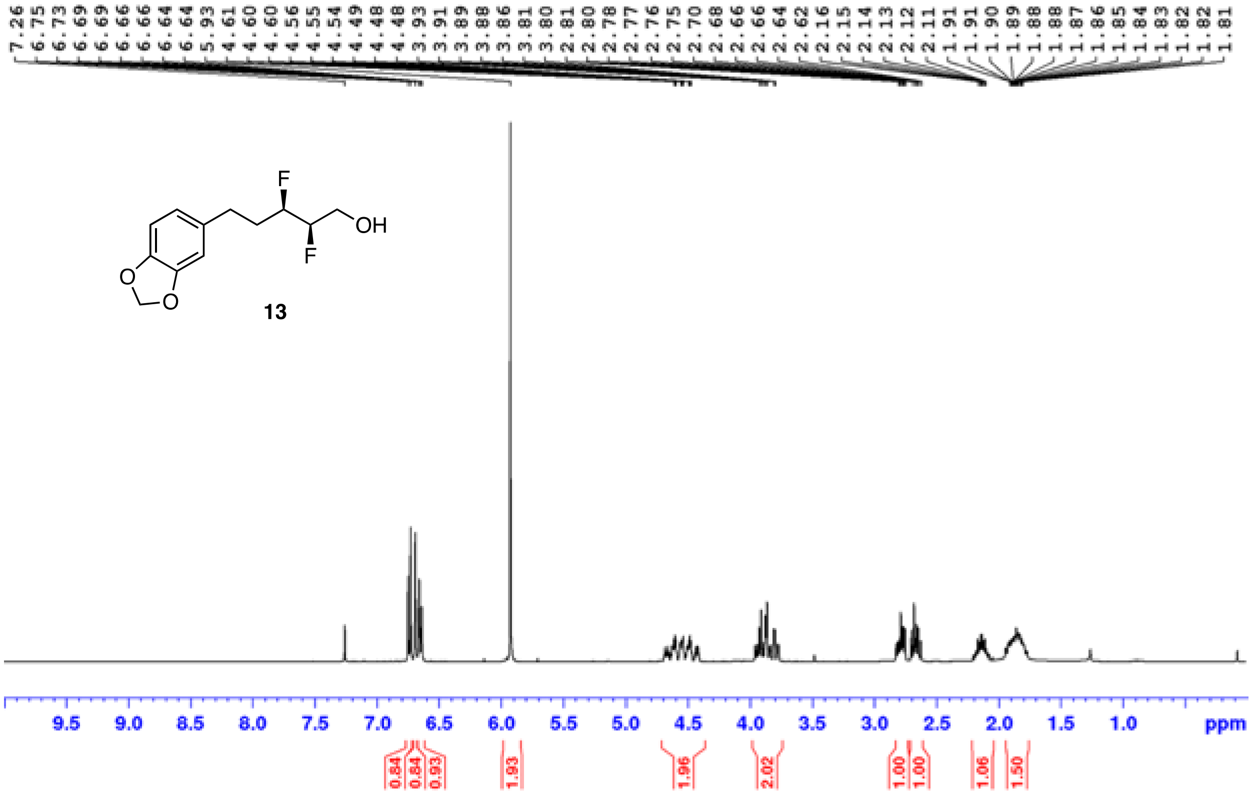

^{13}C{^{1}H} NMR (101 MHz, $CDCl_3$) of **13**

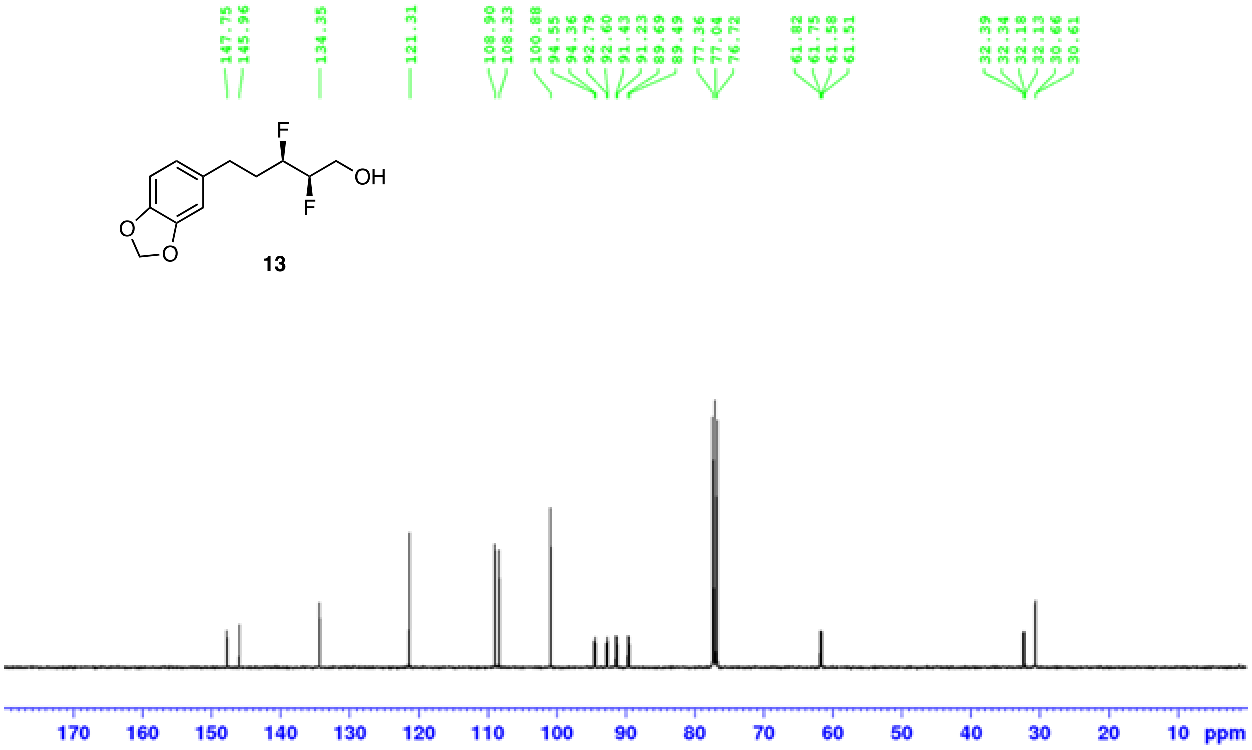

^{19}F NMR (377 MHz, CDCl$_3$) of **13**

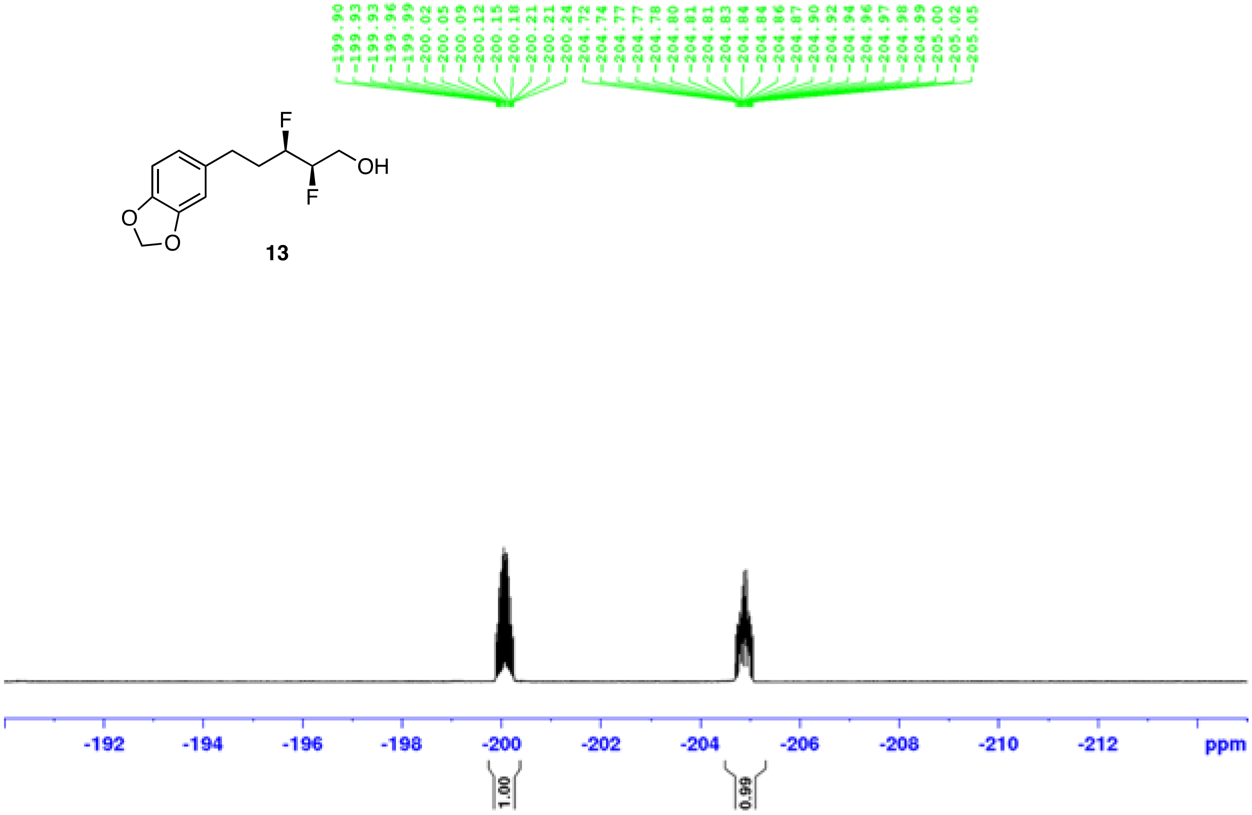

^{19}F{^{1}H} NMR (377 MHz, CDCl$_3$) of **13**

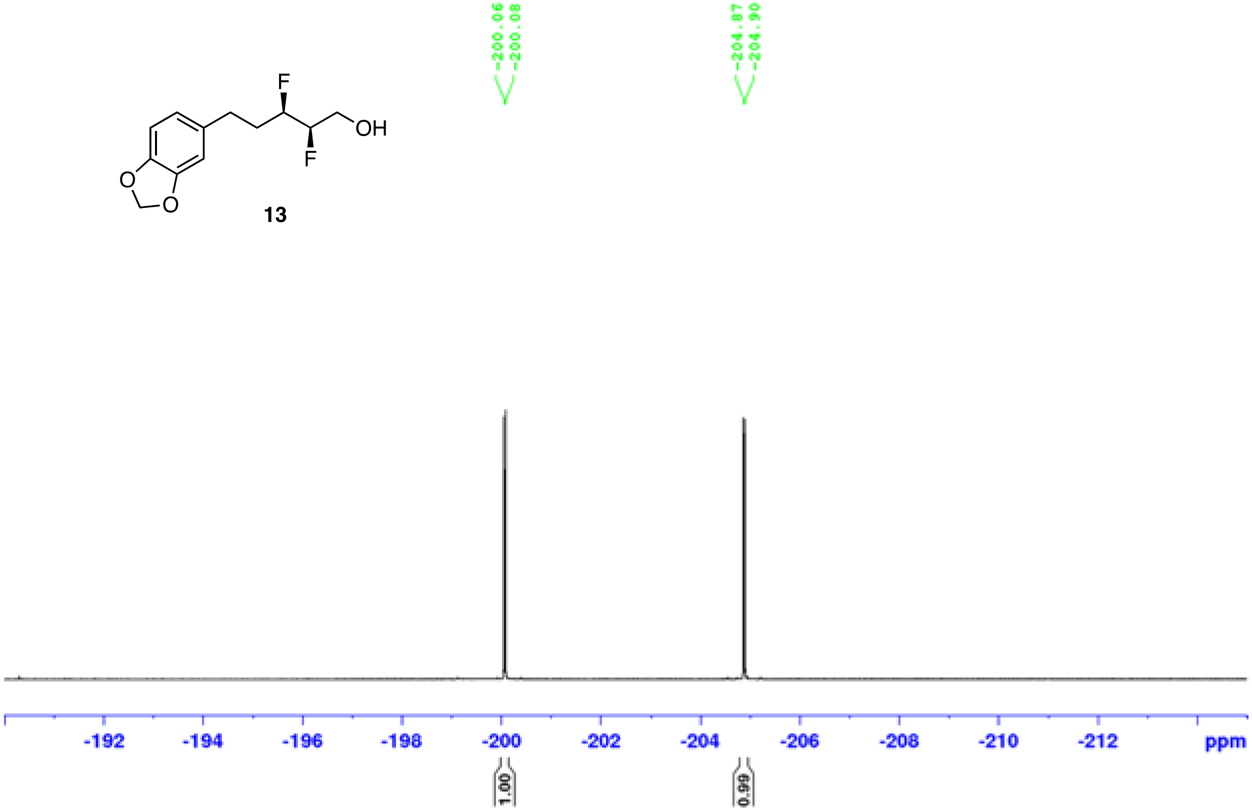

^{1}H NMR (300 MHz, $CDCl_3$) of **16**

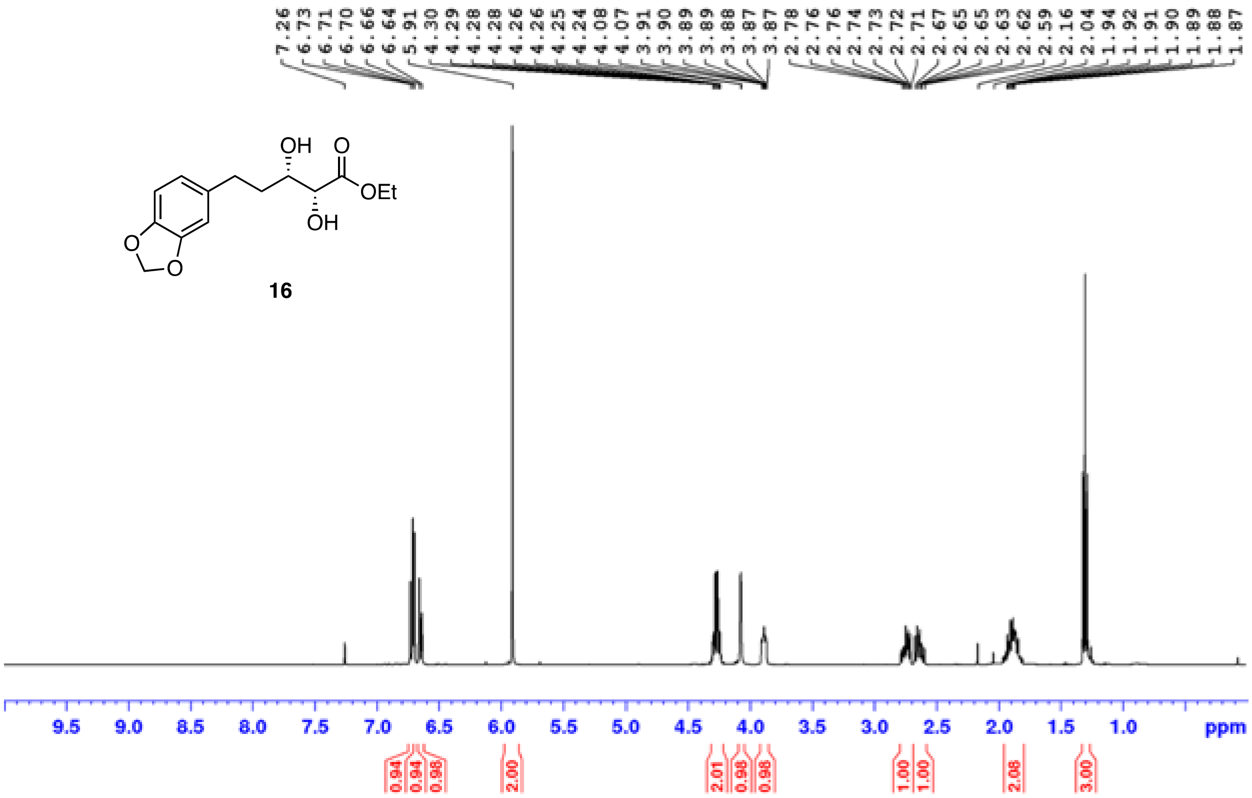

^{13}C{^{1}H} NMR (101 MHz, $CDCl_3$) of **16**

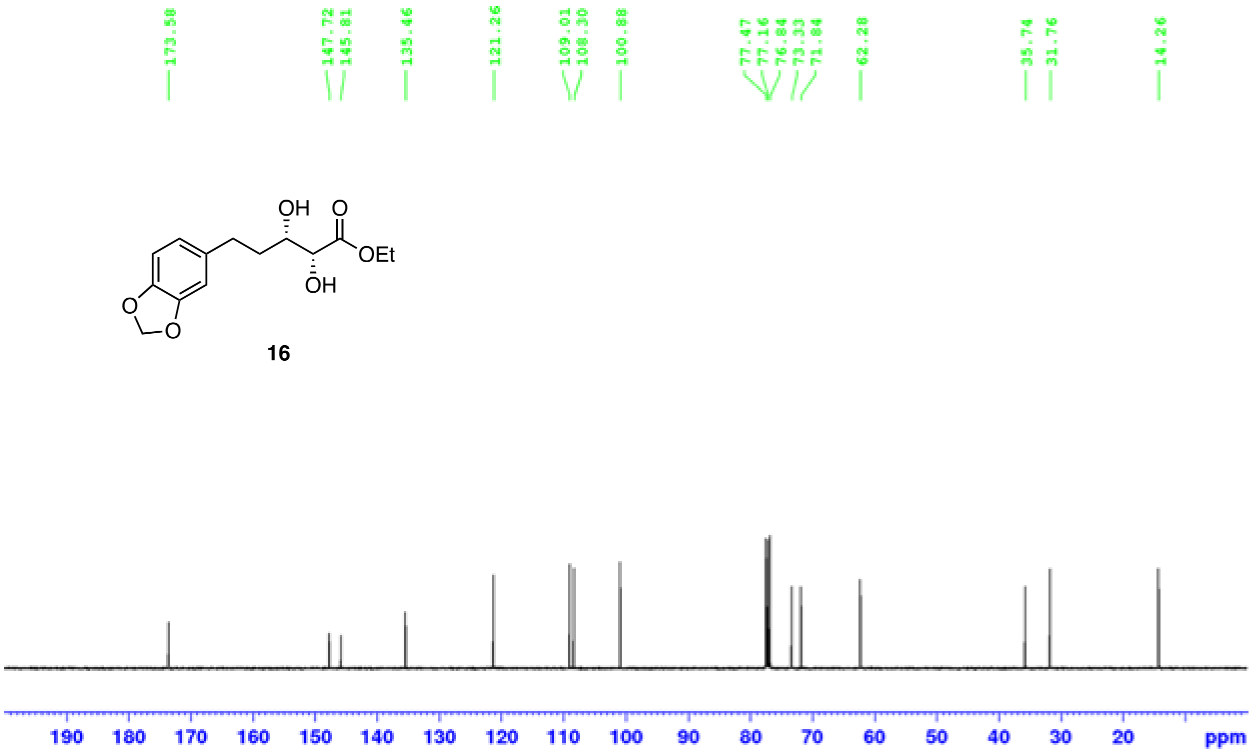

^{1}H NMR (400 MHz, $CDCl_3$) of **17** + cyclic sulfite precursor

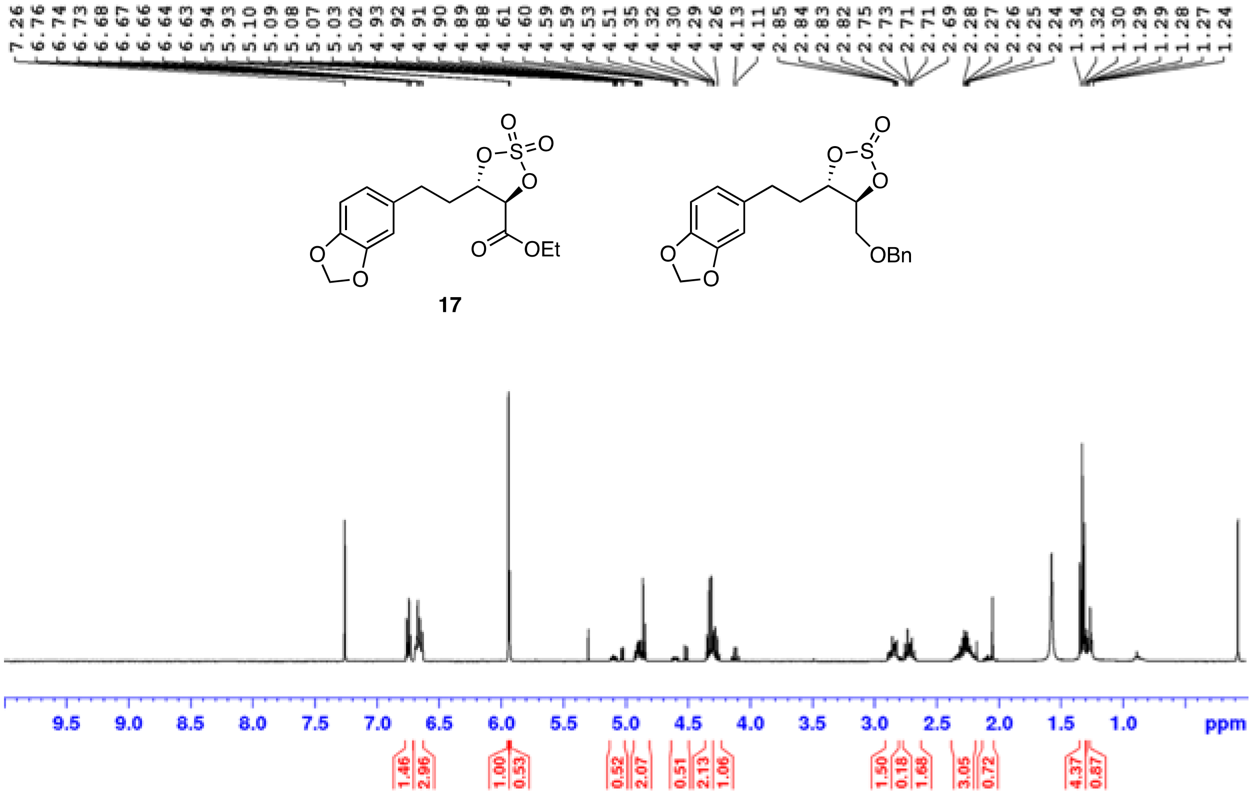

^{13}C{^{1}H} NMR (101 MHz, $CDCl_3$) of **17** + cyclic sulfite precursor

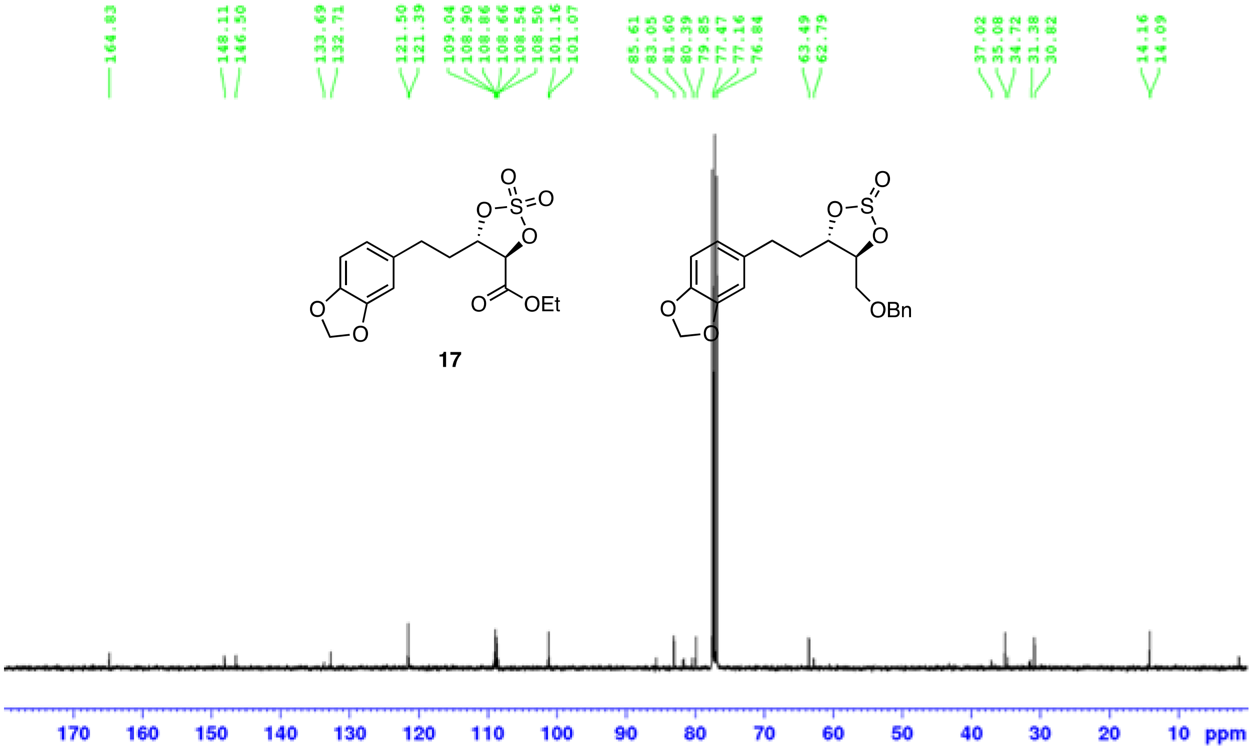

¹H NMR (400 MHz, $CDCl_3$) of **18**

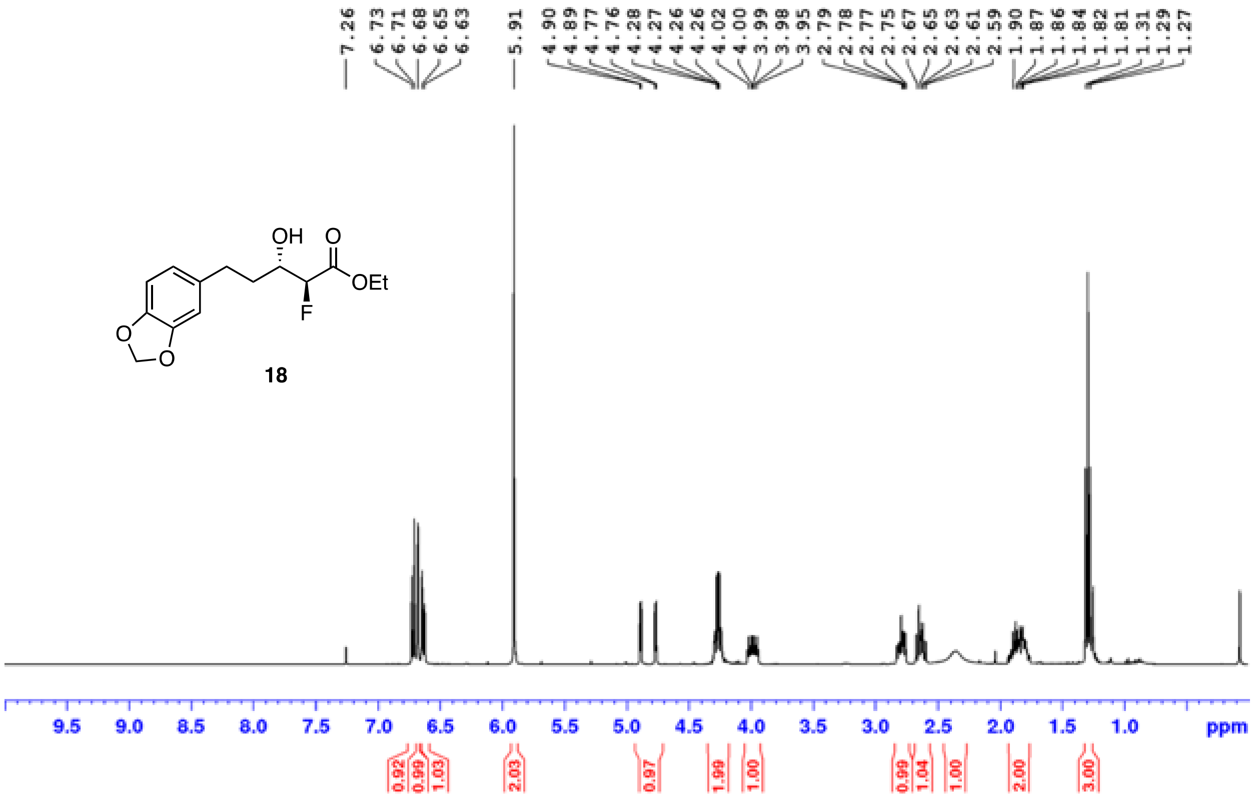

¹³C{¹H} NMR (101 MHz, $CDCl_3$) of **18**

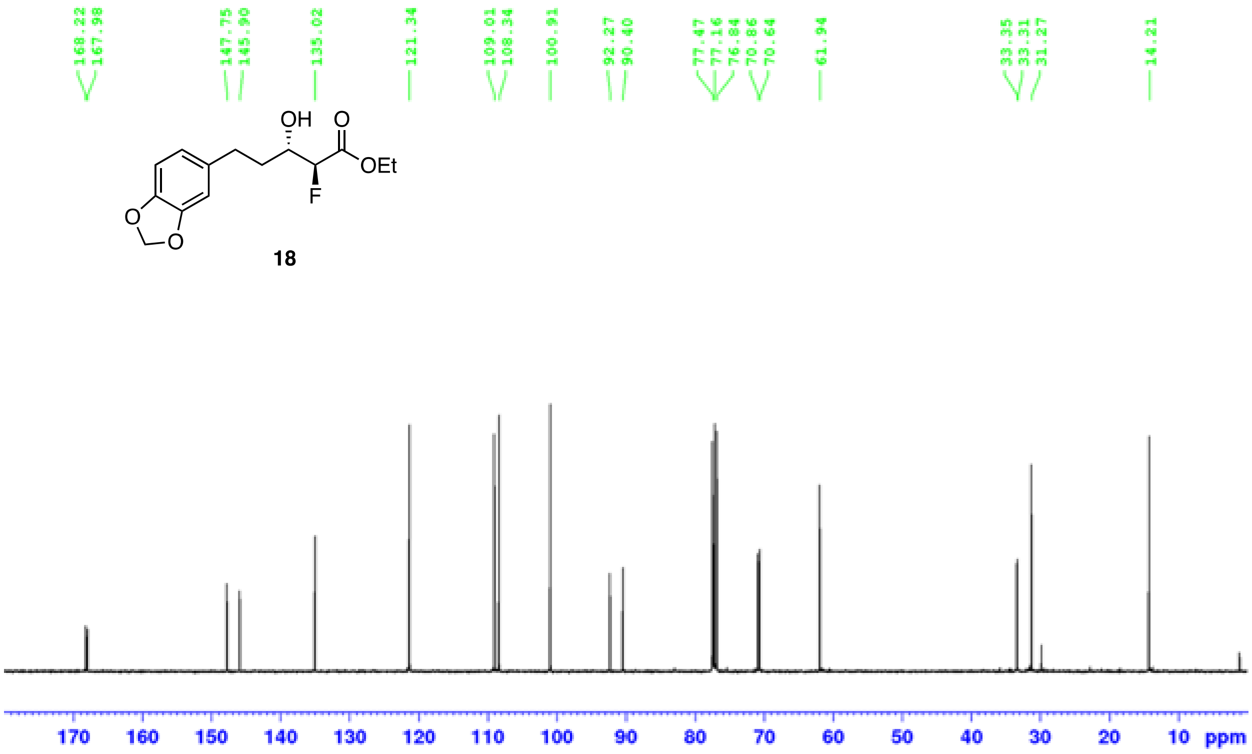

^{19}F NMR (377 MHz, $CDCl_3$) of **18**

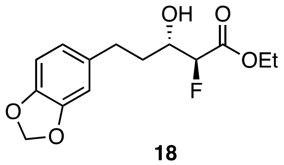

18

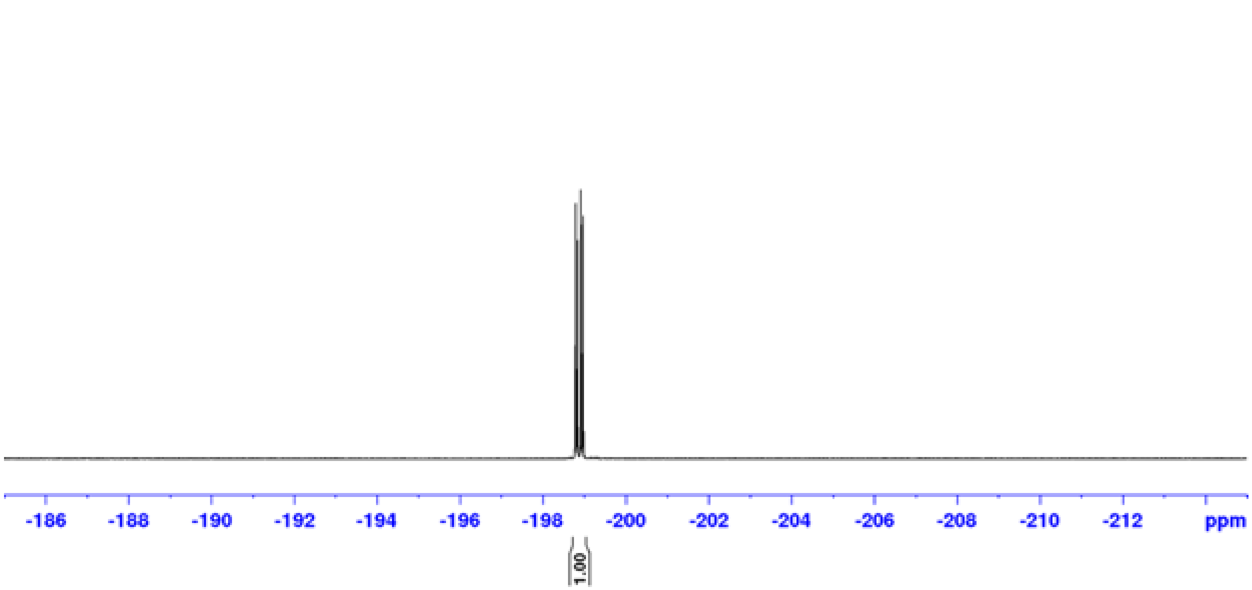

^{19}F {^{1}H} NMR (377 MHz, $CDCl_3$) of **18**

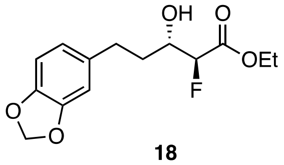

18

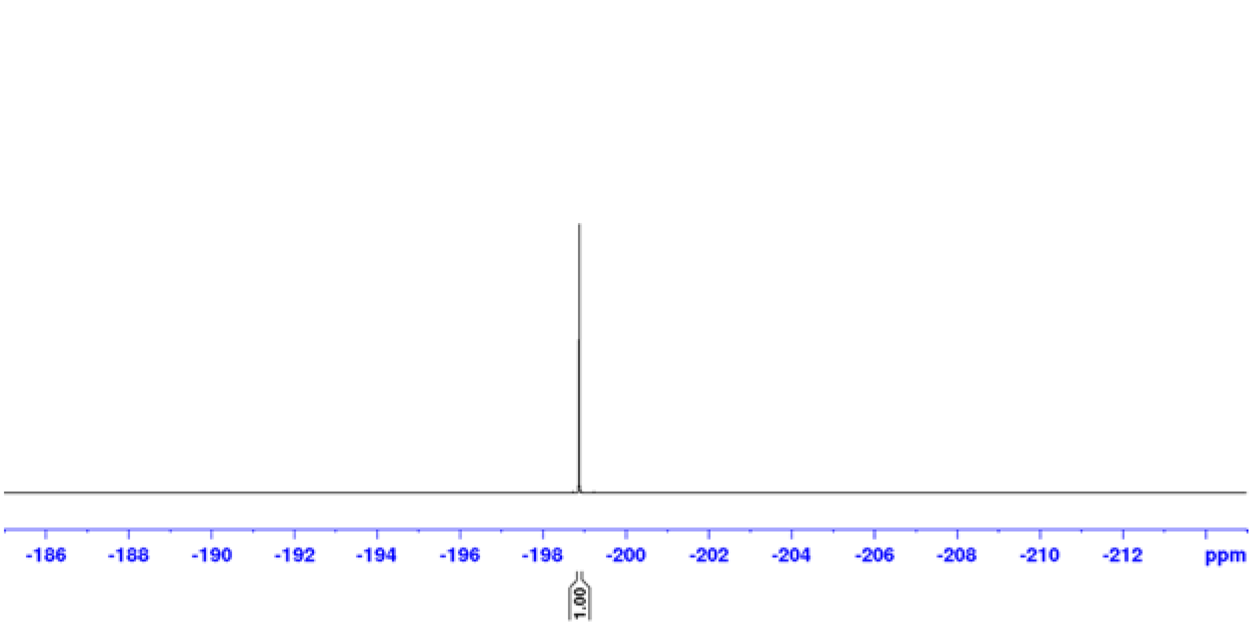

^{1}**H NMR** (300 MHz, $CDCl_3$) of **19**

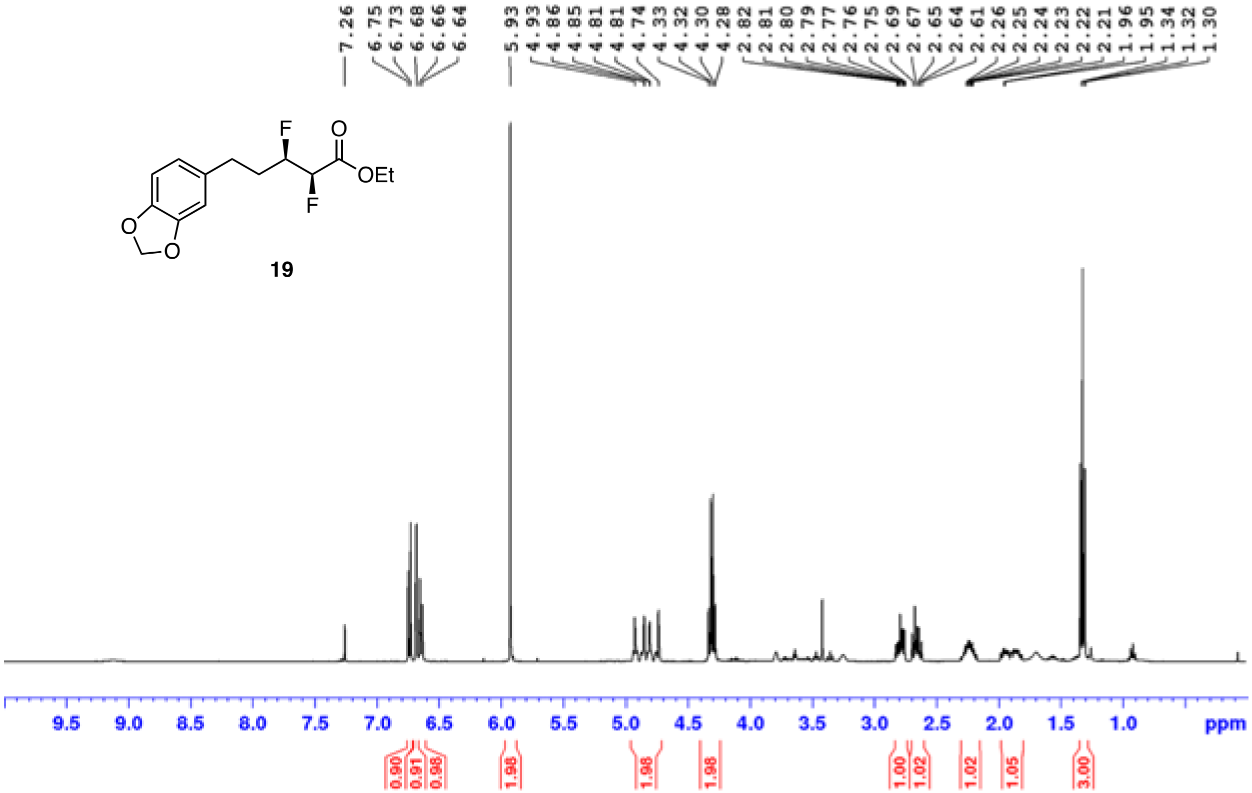

^{13}**C{^{1}H} NMR** (101 MHz, $CDCl_3$) of **19**

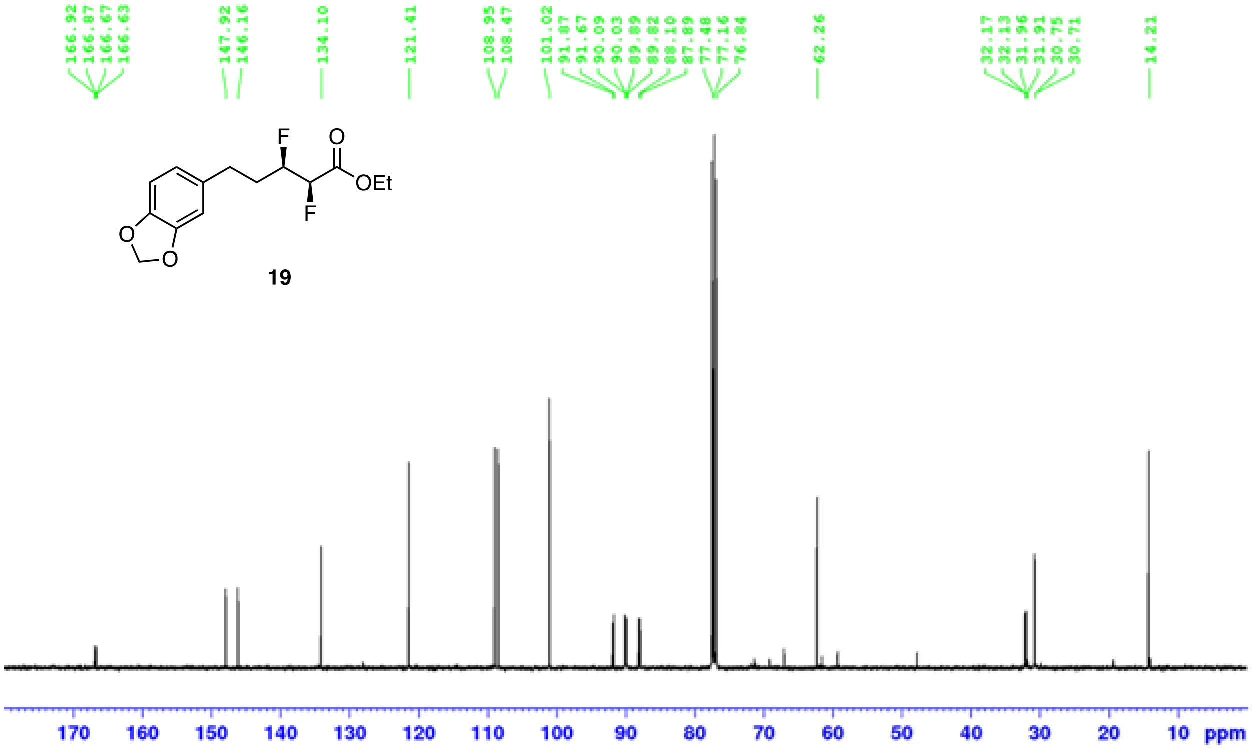

^{19}F **NMR** (377 MHz, $CDCl_3$) of **19**

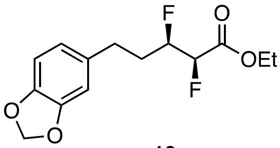

19

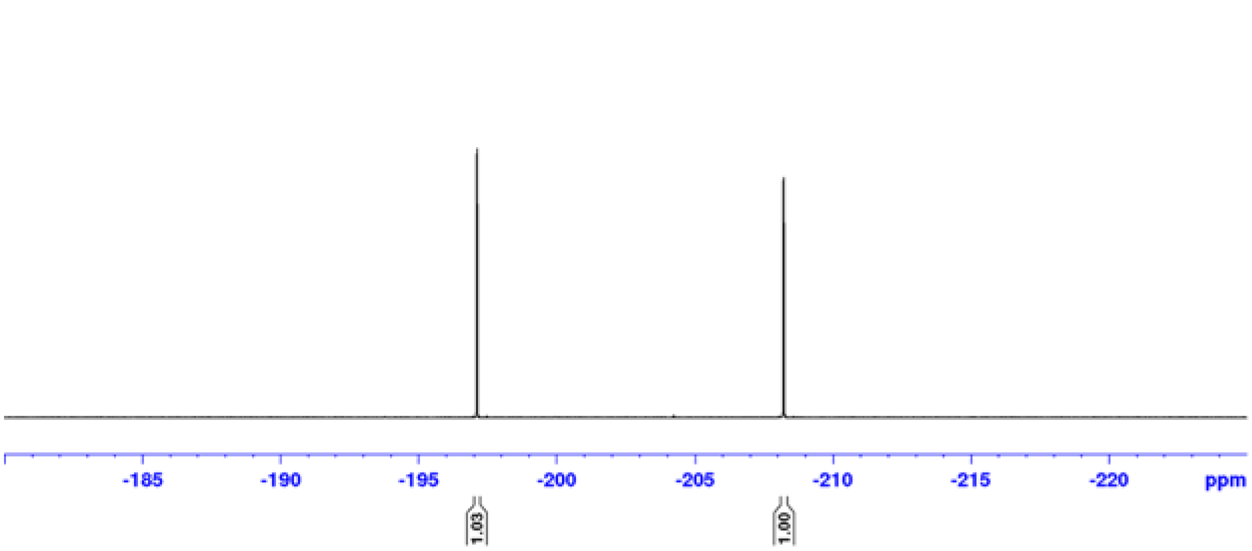

^{19}F {^{1}H} **NMR** (377 MHz, $CDCl_3$) of **19**

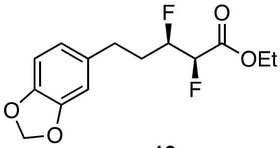

19

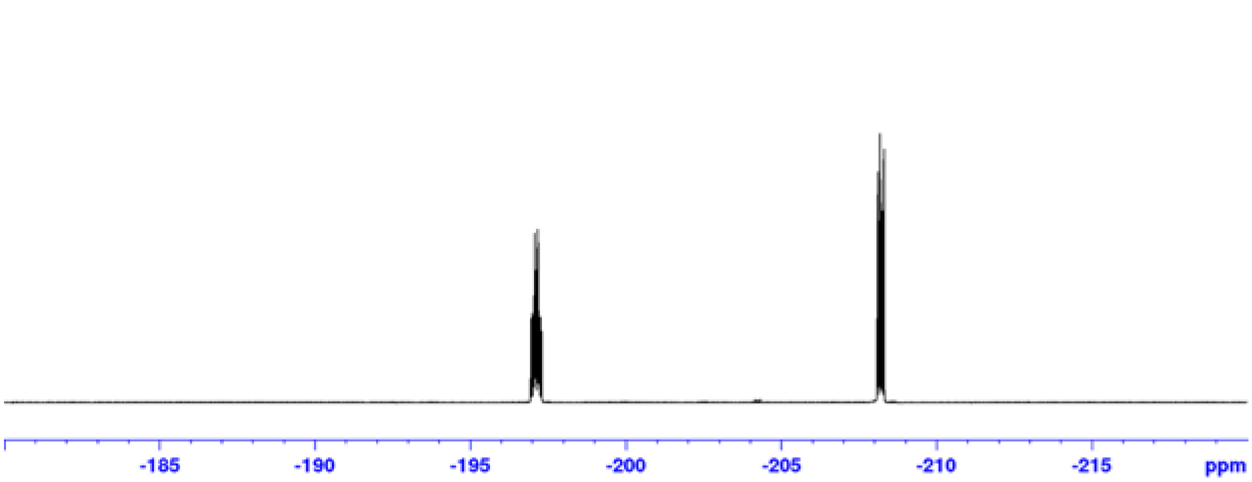

^{1}H NMR (300 MHz, $CDCl_3$) of **20**

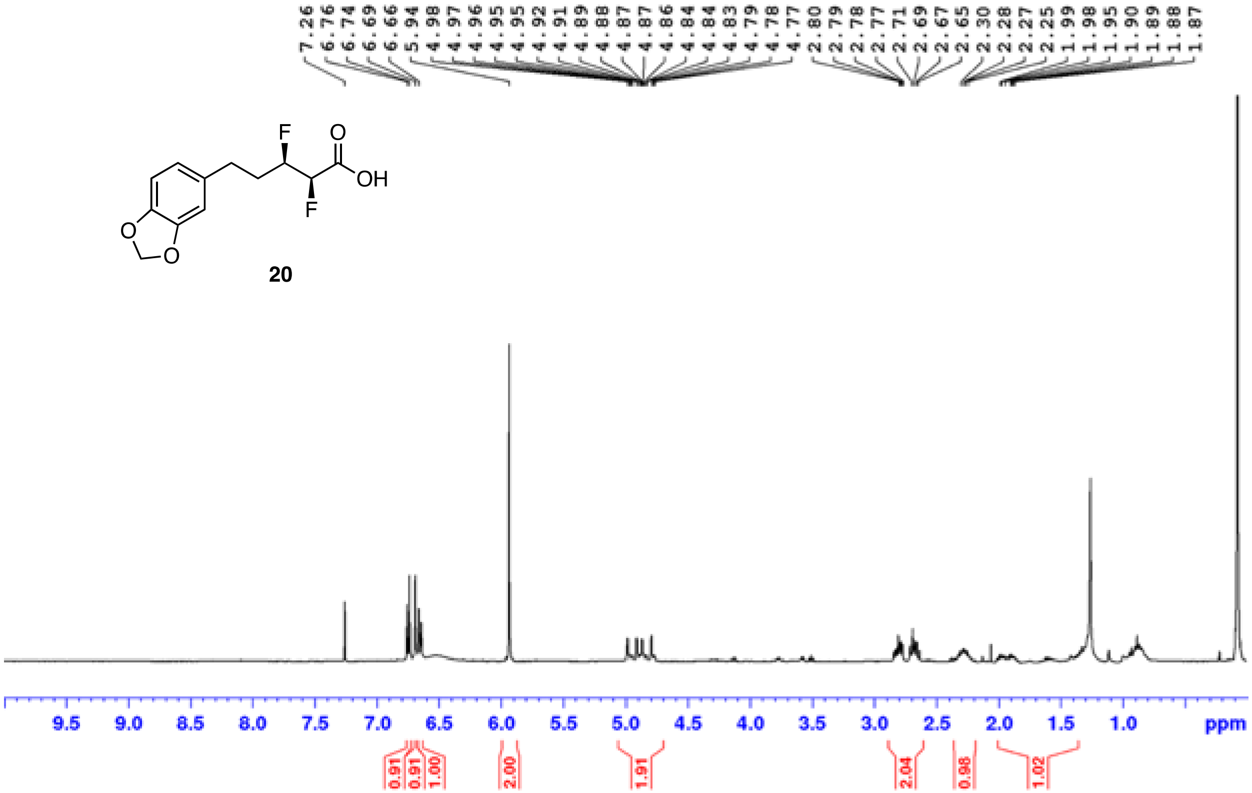

^{13}C{^{1}H} NMR (101 MHz, $CDCl_3$) of **20**

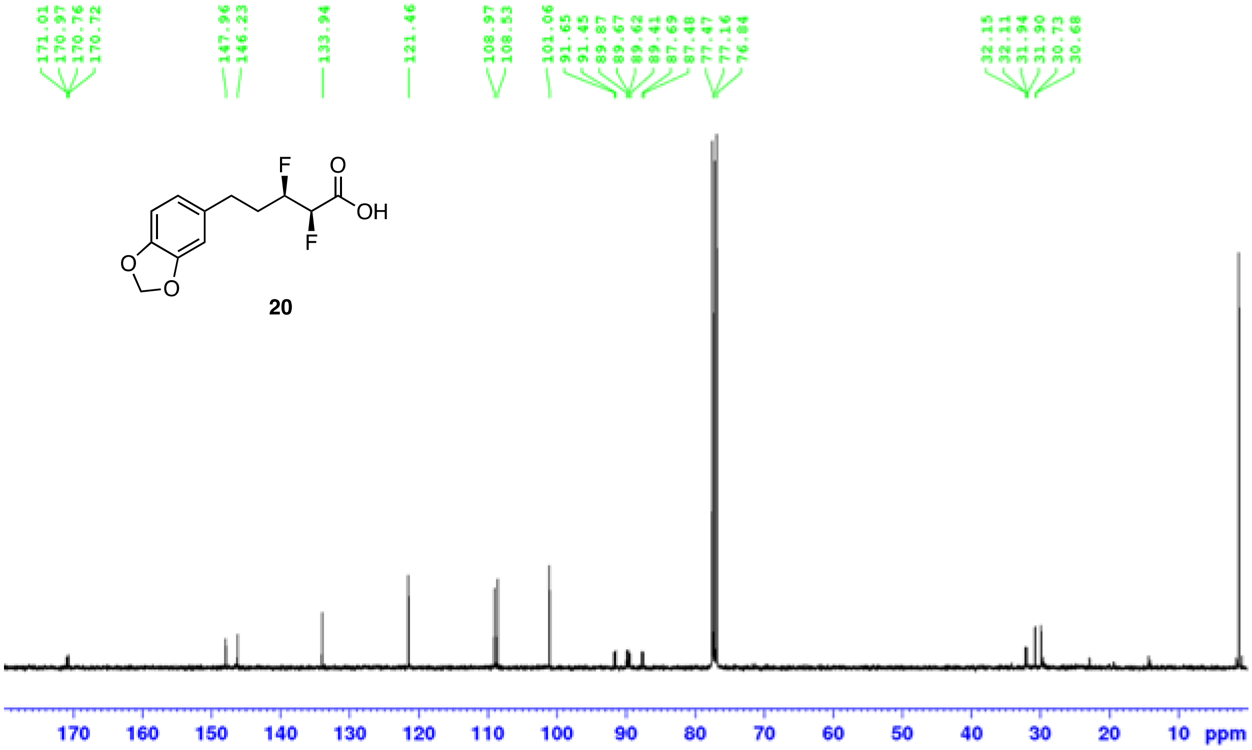

^{19}F NMR (377 MHz, CDCl$_3$) of **20**

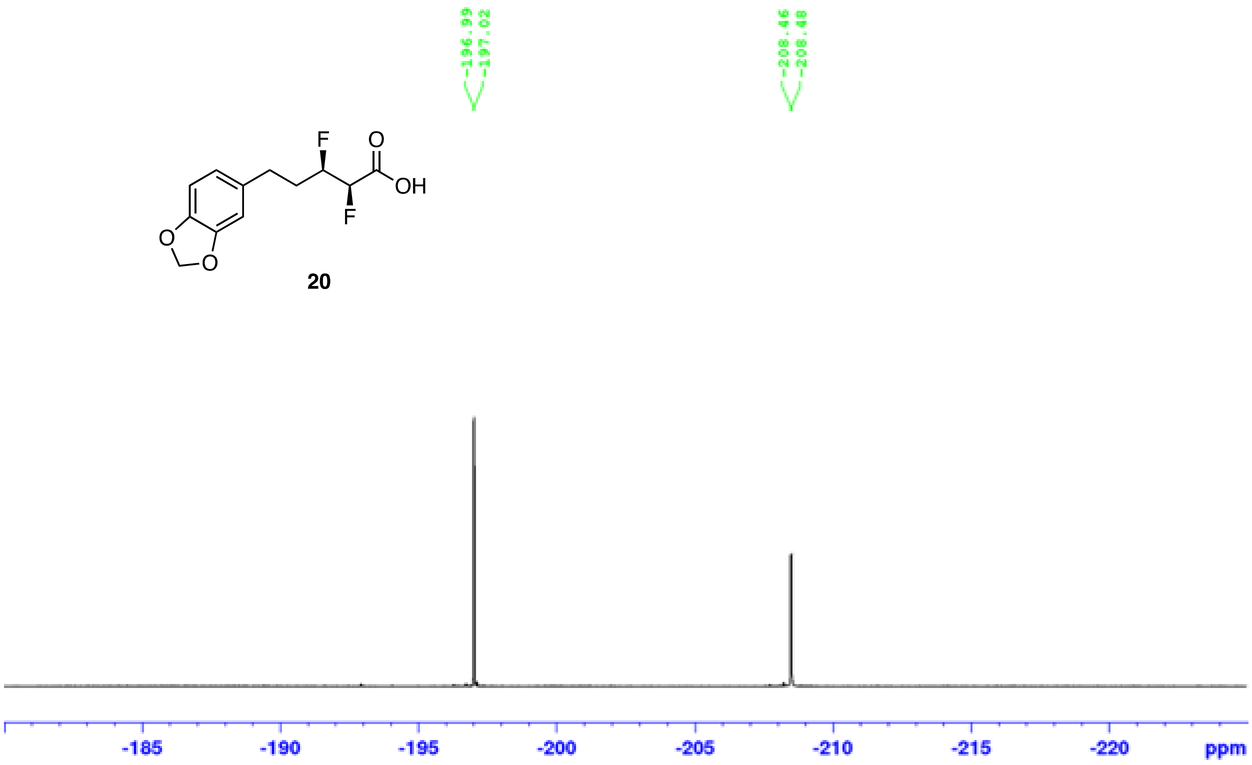

^{19}F {^{1}H} NMR (377 MHz, CDCl$_3$) of **20**

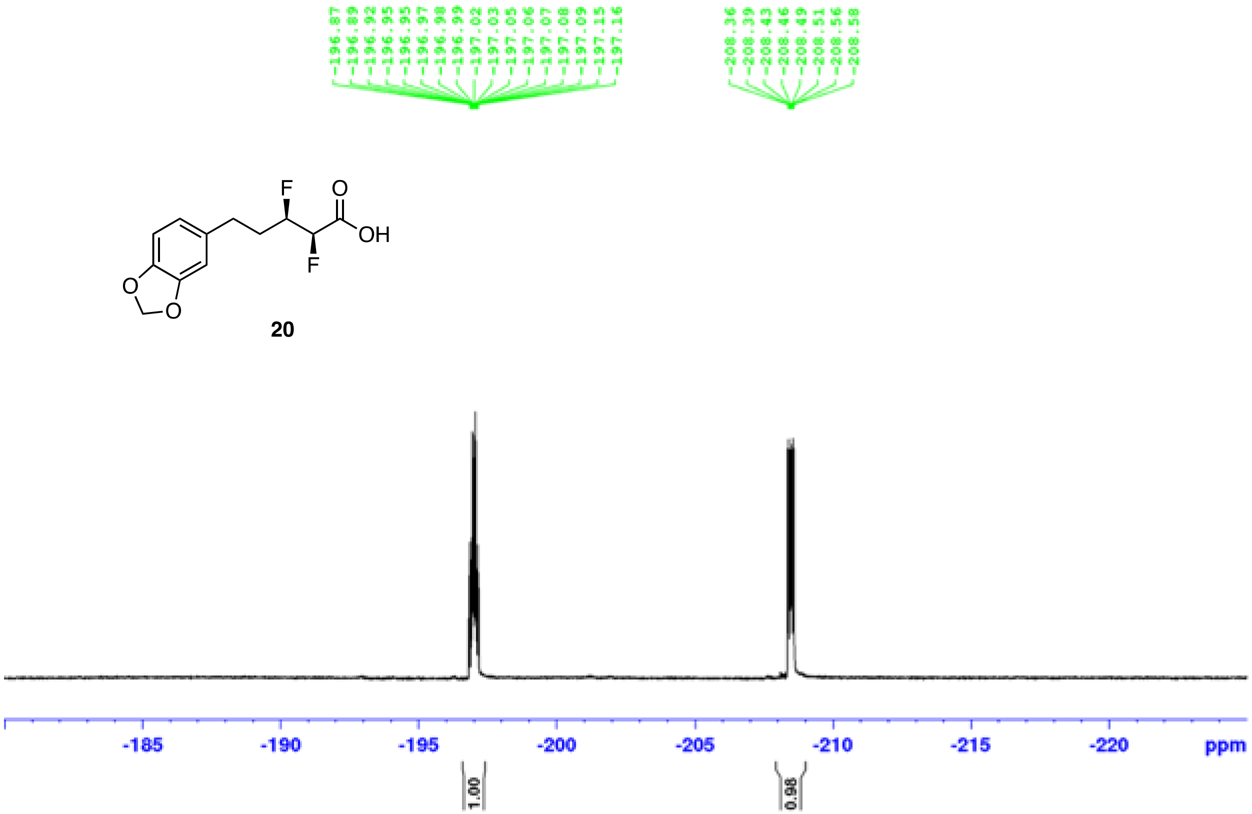

6. Photostability assessment

Piperine (**1**) and analog **2** were dissolved in ethanol in separate clear glass vials. Both vials were exposed to sunlight, side-by-side, for 2.5 hours. The solutions were then concentrated, and each residue was analysed by ^{1}H NMR (see below). Unfortunately, the NMR solvent ($CDCl_3$) seemed to have been contaminated with ethyl acetate in some instances.

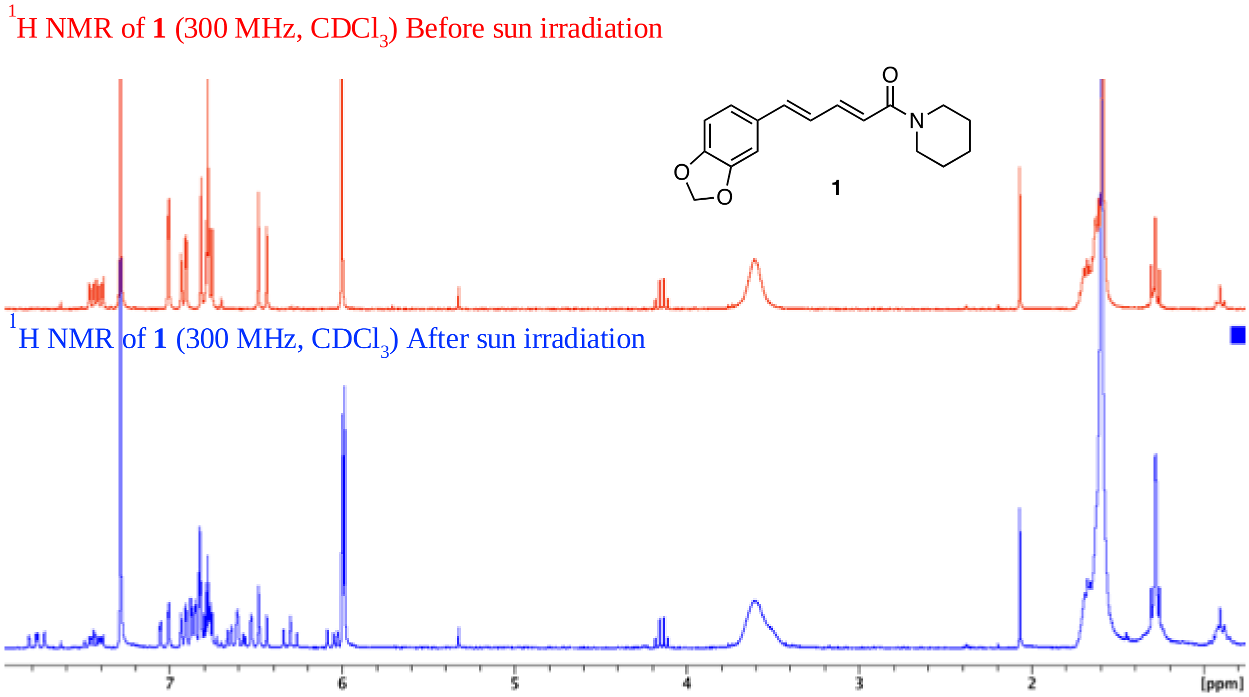

^{1}H NMR of **2** (300 MHz, $CDCl_3$) Before sun irradiation

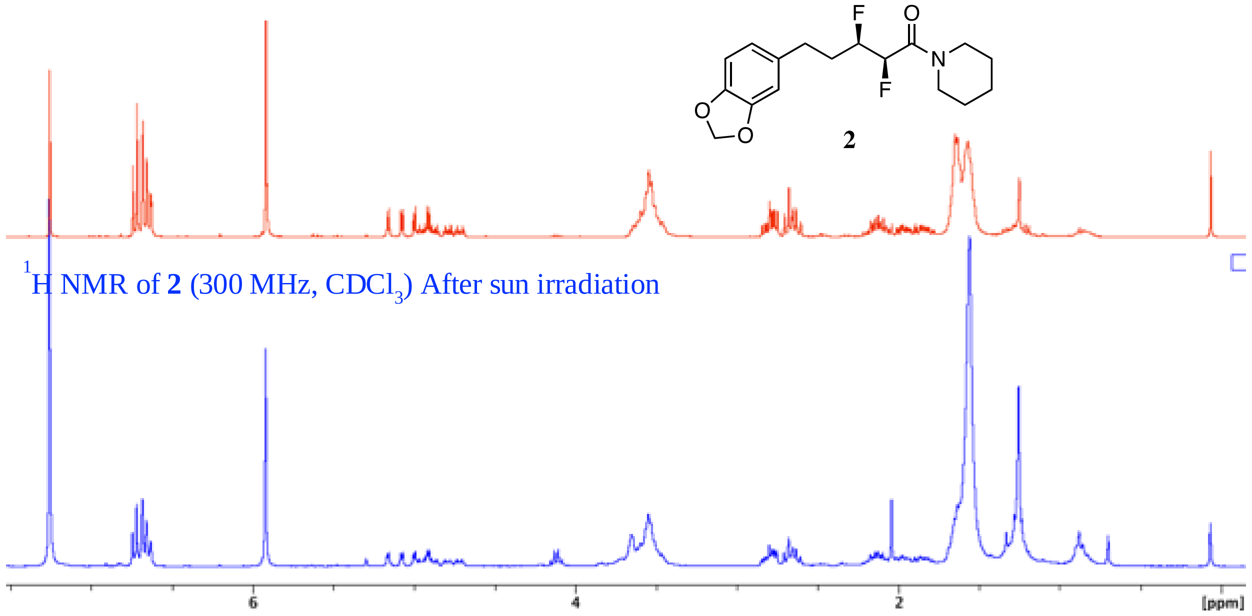

7. Conformational analysis of compound 2

Accurate NMR *J*-values for **2** were obtained by simulating the ^{1}H NMR spectrum of this compound using the Daisy module of the Bruker TopSpin software (see enlargements below).

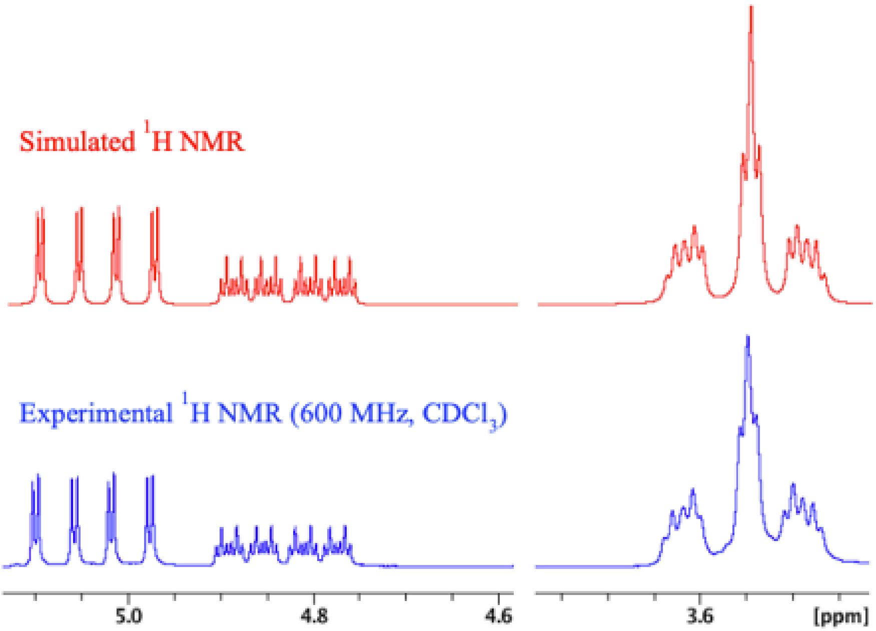

Density functional theory calculations were carried out in Gaussian 09 using a Linux-based computational cluster (Katana, UNSW). Starting conformers of **2** were generated in IQmol by systematically rotating three dihedral angles (F–C–C–F, F–C–C=O and O=C–N–C) in 120° increments using the *rotbond.pl* script developed by Prof. Michelle Coote. All of the starting conformers were then geometry optimised and their energies calculated at the M06-2X level of theory with the 6-311+G(d,p) basis set, and with SMD chloroform solvent. Duplicate final structures were removed. NMR coupling constants were calculated for the lowest energy final structures using the GIAO method at the B3LYP/6-311+G(d,p) level of theory.

Geometry	3D structure	Relative energy (kJ/mol) in chloroform
**2a**	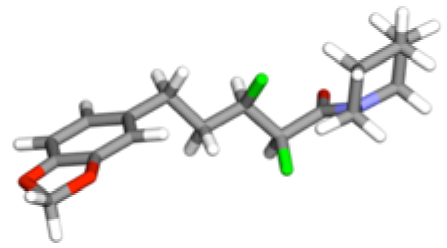	0.00

**2b**	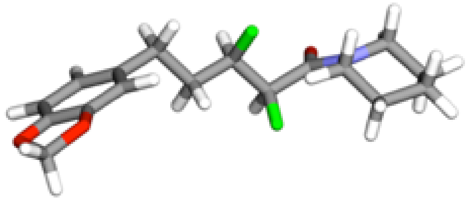	+1.7
**2c**	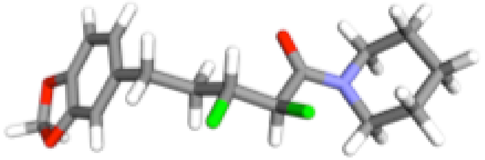	+5.1
**2d**	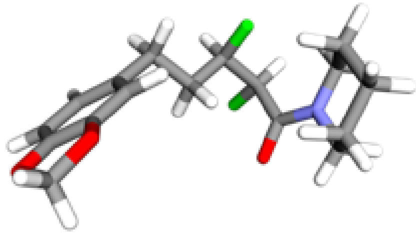	+5.6
**2e**	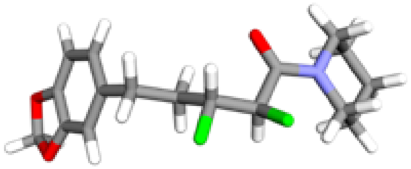	+7.6
**2f**	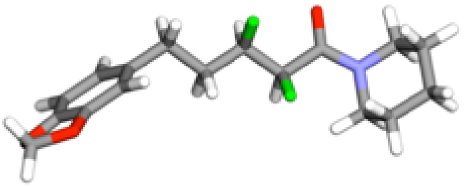	+8.0
**2g**	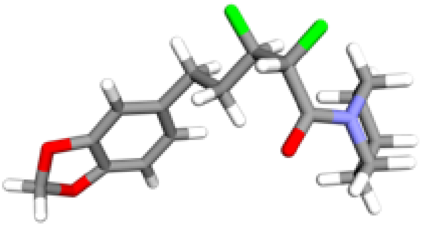	+8.3
**2h**	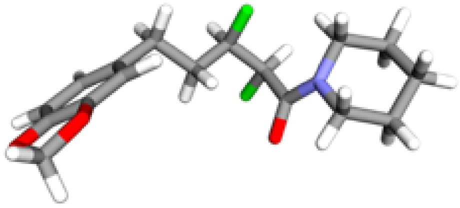	+8.8
**2i**	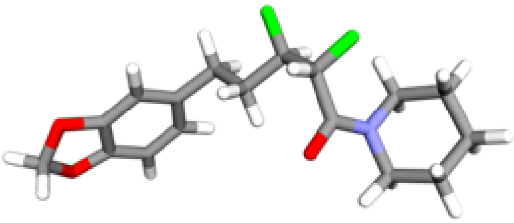	+8.9

**2j**	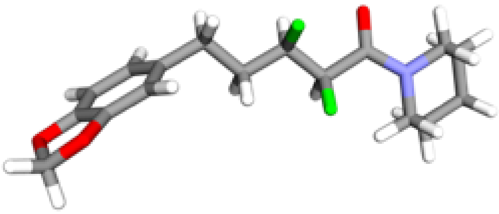	+9.4
**2k**	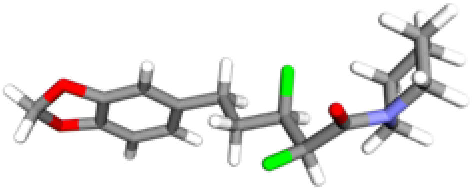	+11.8
**2l**	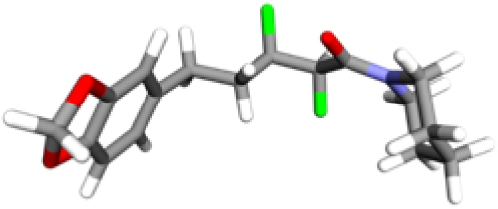	+14.0
**2m**	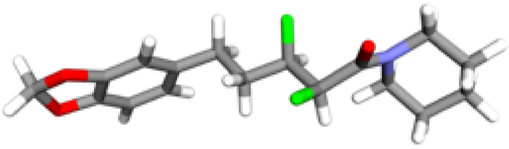	+15.1
**2n**	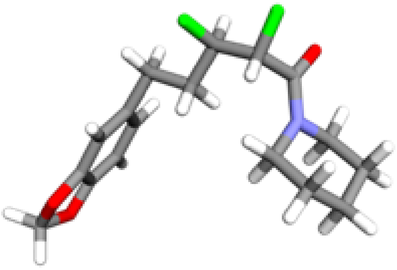	+15.6
**2o**	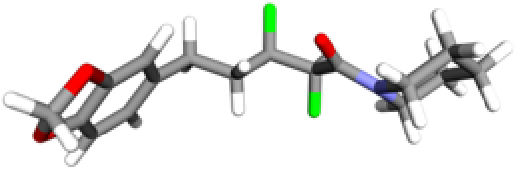	+15.7
**2p**	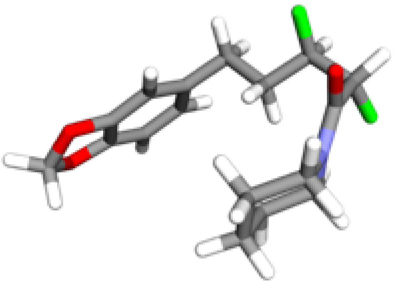	+16.3

**2q**	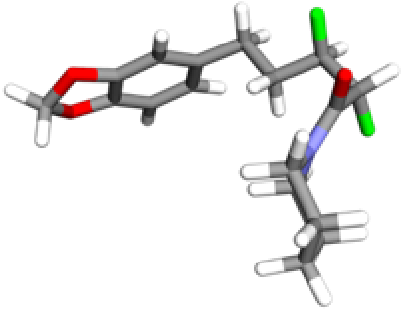	+18.1

8. Biological assays

AChE inhibition assay[5]

5,5′-Dithiobis(2-nitrobenzoic acid) (DTNB), acetylthiocholine (ATCI), and acetylcholinesterase from Electrophorus electricus (AChE) were purchased from Sigma Aldrich. The absorbance at 415 nm was measured using an iMark BioRad plate reader.

Three buffers were used in the assay at pH 8: buffer A (50 mM Tris-HCl); buffer B (50 mM Tris-HCl and 0.1% bovine serum albumin), and buffer C (50 mM Tris-HCl, 100 mM NaCl and 20 mM $MgCl_2$). Fresh solutions were prepared for each experiment: ATCl (15 mM in milli-Q water), DTNB (3 mM in buffer C), and AChE (0.26 U/mL in buffer A).

To each 96-well plate, DTNB (100 µL, 3 mM), AChE (20 µL of 0.26 U/mL), buffer B, and the test compounds (20 µL) at different concentrations or buffer A (20 µL, non-specific) were added. The plate was then mixed and incubated for 15 min at rt. The initial absorbances were measured at 415 nm, then ATCl (20 µL) was added and the absorbance was measured at 415 nm every 2 min for 20 reads. The absorbance versus time was plotted for each well, giving a linear relation whose slope was used to calculate the AChE inhibition activity using the equation below (S_{NS} is the slope of the non-specific control well (buffer A) and S_X is the slope of a test well).

$$\%AChEI = \frac{(S_{NS} - S_X)}{S_{NS}} \times 100\%$$

BACE-1 inhibition assay[6]

The BACE-1 inhibition assay was carried out using the Reaction Biology Corp assay protocol. Briefly, Recombinant Human BACE-1 (Accession # P56817.1) expressed in mouse myeloma cell line was dissolved in freshly prepared reaction buffer (100 mM sodium acetate, pH 4.0, with 1% DMSO added before use). This enzyme solution was delivered into the reaction well by acoustic technology (Echo550; LabCyte Inc. Sunnyvale, CA) in the nanolitre range, followed by a solution of the test compounds in DMSO. The mixture was incubated for 10 min at rt. A solution of Fluorogenic Peptide Substrate IV (R&D System Cat# ES004) in freshly prepared reaction buffer was then dispensed into the reaction well to initiate the reaction. The enzyme activity was monitored every 5 min as a time-course measurement of the increase in the fluorescence signal from a fluorescently

labelled peptide substrate for 120 min at rt. The data was analysed by taking the slope (signal/time) of the linear portion of the measurement, and calculating the slope using MSExcel. Curve fits were performed using the Graphpad Prism software.

9. References

(1) De Araújo-Júnior, J. X.; de M. Duarte, C.; Maria, M. C.; Parente, J. P.; Fraga, C. A. M.; Barreiro, E. J. *Synth. Commun.* **2001**, *31*, 117.

(2) Wang, Z.; Hunter, L. *J. Fluorine Chem.* **2012**, *143*, 143.

(3) Lizarme, Y.; Wangsahardja, J.; Marcolin, G. M.; Morris, J. C.; Jones, N. M.; Hunter, L. *Bioorg. Med. Chem.* **2016**, *24*, 1480.

(4) Dale, J. A.; Mosher, H. S. *J. Am. Chem. Soc.* **1973**, *95*, 512.

(5) Mathew, M.; Subramanian, S. *PLoS One* **2014**, *9*, e86804.

(6) Stachel, S. J.; Coburn, C. A.; Steele, T. G.; Jones, K. G.; Loutzenhiser, E. F.; Grego, A. R.; Rajapakse, H. A.; Lai, M.-T.; Crouthamel, M.-C.; Xu, M.; Tugusheva, K.; Linegerger, J. E.; Pietrak, B. L.; Espeseth, A. S.; Shi, X.-P.; Chen-Dodson, E.; Holloway, M. K.; Munshi, S.; Simon, A. J.; Kuo, L.; Vacca, J. P. *J. Med. Chem.* **2004**, *47*, 6447.
